# *Salmonella* spp. in Domestic Ruminants, Evaluation of Antimicrobial Resistance Based on the One Health Approach—A Systematic Review and Meta-Analysis

**DOI:** 10.3390/vetsci11070315

**Published:** 2024-07-14

**Authors:** Juan García-Díez, Dina Moura, Luca Grispoldi, Beniamino Cenci-Goga, Sónia Saraiva, Filipe Silva, Cristina Saraiva, Juan Ausina

**Affiliations:** 1 Veterinary and Animal Research Centre (CECAV), University of Trás-os-Montes e Alto Douro, Quinta de Prados, 5000-801 Vila Real, Portugal; soniasaraiva@utad.pt (S.S.); fsilva@utad.pt (F.S.); crisarai@utad.pt (C.S.); 2 Associate Laboratory for Animal and Veterinary Science (AL4AnimalS), 1300-477 Lisboa, Portugal; 3 Divisão de Intervenção de Alimentação e Veterinária de Vila Real e Douro Sul, Direção de Serviços de Alimentação e Veterinária da Região Norte, Direção Geral de Alimentação e Veterinária, Lugar de Codessais, 5000 Vila Real, Portugal; dina.moura@dgav.pt; 4 Dipartamento di Medicina Veterinaria, Universitá degli Studi di Perugia, 06126 Perugia, Italy; grisluca@outlook.it (L.G.); beniamino.cencigoga@unipg.it (B.C.-G.); 5 Faculty of Veterinary Science, Department of Paraclinical Sciences, University of Pretoria, Onderstepoort 0110, South Africa; 6 Department of Veterinary Sciences, School of Agricultural and Veterinary Sciences, Universidade de Trás-os-Montes e Alto Douro, Quinta de Prados, 5000-801 Vila Real, Portugal; 7 Social Psychology and Methodology Department, Universidad Autónoma de Madrid, Ciudad Universitaria de Cantoblanco, 28049 Madrid, Spain; juan.botella@uam.es

**Keywords:** *Salmonella* spp., antimicrobial resistance, cattle, sheep, goat, one health, food safety, public health

## Abstract

**Simple Summary:**

Salmonella continues to pose a significant threat to public health, standing as the second leading cause of foodborne illnesses in the European Union. In instances of severe infection, the treatment of salmonellosis necessitates the use of antimicrobials, among other therapeutic interventions. The escalating resistance of *Salmonella* spp. to antibiotics in recent years, primarily attributed to inappropriate usage in livestock, has raised considerable concerns among health authorities. The findings indicate that the low prevalence of *Salmonella* spp. isolated from samples of cattle, sheep, and goats in slaughterhouses, coupled with their comparatively low-to-moderate resistance to key antibiotics used in the treatment of human salmonellosis, suggests that the consumption of beef, lamb, and goat meat does not pose a substantial threat to public health in relation to the proliferation of antimicrobial resistance (AMR).

**Abstract:**

*Salmonella* spp. pose a global threat as a leading cause of foodborne illnesses, particularly prevalent in the European Union (EU), where it remains the second cause of foodborne outbreaks. The emergence of antimicrobial resistance (AMR) in *Salmonella* spp. has become a critical concern, complicating treatment strategies and escalating the risk of severe infections. The study focuses on large and small ruminants, identifying a prevalence of *Salmonella* spp. in slaughterhouses and revealing varied AMR rates across antimicrobial families throughout a meta-analysis. Also, comparison with AMR in human medicine was carried out by a systematic review. The results of the present meta-analysis displayed a prevalence of *Salmonella* spp. in large and small ruminants at slaughterhouses of 8.01% (8.31%, cattle; 7.04%, goats; 6.12%, sheep). According to the AMR of *Salmonella* spp., 20, 14, and 13 out of 62 antimicrobials studied were classified as low (<5%), high (>5% but <10%), and very high (>10%), respectively. *Salmonella* spp. did not display AMR against aztreonam, mezlocillin, ertapenem, meropenem, cefoxitin, ceftazidime, levofloxacin, tilmicosin, linezolid, fosfomycin, furazolidone, quinupristin, trimethoprim and spectinomycin. In contrast, a prevalence of 100% of AMR has been described against ofloxacin, lincomycin, and cloxacillin. In the context of the main antibiotics used in the treatment of human salmonellosis, azithromycin was shown to have the highest resistance among *Salmonella* spp. isolates from humans. Regarding cephalosporins, which are also used for the treatment of salmonellosis in humans, the prevalence of *Salmonella* spp. resistance to this class of antibiotics was similar in both human and animal samples. Concerning quinolones, despite a heightened resistance profile in *Salmonella* spp. isolates from ruminant samples, there appears to be no discernible compromise to the efficacy of salmonellosis treatment in humans since lower prevalences of AMR in *Salmonella* spp. isolated from human specimens were observed. Although the resistance of *Salmonella* spp. indicates some degree of concern, most antibiotics are not used in veterinary medicine. Thus, the contribution of cattle, sheep and goats to the rise of antibiotic resistance of *Salmonella* spp. and its potential impact on public health appears to be relatively insignificant, due to their low prevalence in carcasses and organs. Nevertheless, the observed low prevalence of *Salmonella* spp. in ruminants at slaughterhouse and the correspondingly low AMR rates of *Salmonella* spp. to key antibiotics employed in human medicine do not indicate that ruminant livestock poses a substantial public health risk concerning the transmission of AMR. Thus, the results observed in both the meta-analysis and systematic review suggests that AMR is not solely attributed to veterinary antibiotic use but is also influenced by factors such as animal health management (i.e., biosecurity measures, prophylactic schemes) and human medicine.

## 1. Introduction

Salmonellosis is a common foodborne illness caused by various strains of the Salmonella bacterium. It affects the gastrointestinal tract and can lead to a range of symptoms from mild gastroenteritis to severe and sometimes life-threatening conditions [1]. The *Salmonella* genus classified into two species: *S. bongori* and *S. enterica.* Most serotypes classified as *S. enterica* subsp. *enterica* are responsible for the majority of salmonellosis cases in humans and warm-blooded animals [2]. *Salmonella* spp. is typically transmitted to humans through the consumption of contaminated food, especially undercooked or raw eggs, poultry, meat, and unpasteurized dairy products. Direct contact with infected animals or their environment can also lead to transmission [3].

Salmonella infections can range from self-limiting gastroenteritis to severe systemic diseases. Antibiotics are employed in cases where there is evidence of invasive infection or systemic complications. Salmonellosis therapy in humans involves the use of antimicrobial such as quinolones, third generation cephalosporins and azithromycin [4,5]. Most antimicrobials described for treatment of salmonellosis in human medicine are presented in Table 1. Among quinolones, ciprofloxacin and levofloxacin, are commonly used for treating severe salmonellosis. They inhibit bacterial DNA replication by targeting DNA gyrase and topoisomerase IV enzymes. However, the emergence of quinolone-resistant Salmonella strains is a growing concern [6,7]. Third generation cephalosporins like ceftriaxone and cefotaxime, are frequently employed as empirical therapy for severe salmonellosis. These antibiotics inhibit bacterial cell wall synthesis, leading to bacterial death. Third generation cephalosporins are effective against most Salmonella serotypes and are often the treatment of choice in invasive infections [8,9]. Azithromycin, a macrolide antibiotic, is an alternative treatment option for salmonellosis. It acts by binding to the 50S ribosomal subunit, inhibiting protein synthesis. Azithromycin is effective against many Salmonella serotypes, including those where fluoroquinolone-resistance is common [10]. Aminoglycosides are not recommended because, despite demonstration of good in vitro activity, they have poor clinical efficacy [11].

**Table 1:** Antimicrobials referred in the literature to treat salmonellosis in human medicine.

Antimicrobial	Family	References
Amoxicillin	Penicillin	[12,13,14]
Ampicillin	Penicillin	[13,14,15,16,17,18]
Azithromycin	Macrolide	[12,13,14,15,17,18]
Aztreonam	Monobactam	[13,14]
Cefixime	3rd gen. cephalosporin	[13]
Cefoperazone	3rd gen. cephalosporin	[12]
Cefotaxime	3rd gen. cephalosporin	[12,13,14,17]
Ceftazidime	3rd gen. cephalosporin	[14]
Ceftriaxone	3rd gen. cephalosporin	[12,13,14,15,17,18,19,20,21]
Chloramphenicol	Amphenicol	[12,13,14,17,21]
Ciprofloxacin	Quinolone	[12,13,14,17,19,20,21,22,23]
Delafloxacin	Quinolone	[22]
Ertapenem	Carbapenem	[14]
Fleroxacin	Quinolone	[12]
Gatifloxacin	Quinolone	[12,14,22]
Gemifloxacin	Quinolone	[19,22]
Imipenem	Carbapenem	[12,14]
Levofloxacin	Quinolone	[14,19,22]
Meropenem	Carbapenem	[12,14,18]
Moxalactam	Beta-lactam	[14]
Moxifloxacin	Quinolone	[17,19,22]
Nalidixic acid	Quinolone	[17,22]
Norfloxacin	Quinolone	[14,22]
Ofloxacin	Quinolone	[12,14,22]
Pefloxacin	Quinolone	[12,14,22]
Tigecycline	Glycylcycline	[12,14,18]
Trimethoprim-sulfamethoxazole	Dihydropyrimidine-sulfonamide	[12,13,14,15,17,18,24]
Trovafloxacin	Fluoronaphthyridone	[14]
Rifaximin	Rifamycin	[25]

## 2. Salmonellosis in Cattle, Sheep and Goats

In large and small ruminants, salmonellosis can result in a range of clinical manifestations, impacting both individual animals and herd health [26]. Cattle salmonellosis, commonly caused by several serotypes of the *S. enterica* (e.g., *S.* Typhimurium, *S.* Dublin), is frequently associated with clinical disease in cattle and calves [27]. In small ruminants, *S.* Abortusovis and *S*. Brandenburg, are associated with systemic disease and abortion in does and ewe. Furthermore, *S*. Abortusovis is unique among salmonella serovars because it is more often associated with abortion rather than septicemia and enteritis [28,29].

The primary modes of transmission involve the ingestion of contaminated feed, water, or contact with contaminated environments. Stressful conditions, overcrowding, and poor sanitation practices can exacerbate the risk of infection within herds. Infected animals shed the bacteria through feces, further contaminating the surroundings [30].

Livestock affected by salmonella-induced diarrhea requires supportive care and parenteral antimicrobial therapy. The use of antimicrobial agents is controversial and probably does not influence the gastrointestinal infection [26]. Nevertheless, because Salmonella is an invasive organism, the parenteral use of antimicrobial agents may be beneficial in preventing septicemia. Antimicrobial susceptibility patterns are difficult to predict for Salmonella species, so antimicrobial therapy in large and small ruminants (Table 2) should be based on culture and sensitivity results [31]. In veterinary medicine, the most indicated antibiotics for gastrointestinal salmonellosis are quinolones, while it is oxytetracycline for cases of abortions outbreaks, especially in small ruminants [32].

**Table 2:** Antimicrobial substances described in the literature used to treat salmonellosis in large and small ruminants.

Antimicrobial	Antimicrobial Class	References
Amikacin	Aminoglycoside	[33]
Amoxicillin-clavulanic acid	Penicillin	[34]
Ampicillin	Penicillin	[35]
Apramycin	Aminoglycoside	[34]
Ceftiofur	3rd gen. cephalosporin	[33,36,37]
Cephalotin	1st gen. cephalosporin	[33]
Chloramphenicol	Amphenicol	[33]
Enrofloxacin	Quinolone	[27,32,33,34]
Florfenicol	Amphenicol	[35,36,38]
Gentamicin	Aminoglycoside	[33]
Kanamycin	Aminoglycoside	[33]
Neomycin	Aminoglycoside	[35]
Oxytetracycline	Tetracycline	[33,35,39]
Trimethoprim-sulfamethoxazole	Sulfonamide	[27,33,34,35,36,37]

## 3. Epidemiology, Public Health Significance and Antimicrobial Resistance of *Salmonella* spp.

Salmonellosis is still one of the main causes of foodborne outbreak worldwide. Foodborne outbreaks of salmonellosis in humans are usually derived from foods of animal origin such as meat, eggs or milk among others [1]. Most salmonellosis outbreaks reported happened in summer, showing a seasonal pattern [40]. Reports of European Food Safety Authority (EFSA) and European Centre for Disease Prevention and Control (ECDC) reported salmonellosis as the main cause of foodborne outbreak. Thus, from a total of 2201 cases, 590 were confirmed by laboratory analysis and/or epidemiological evidence [41,42].

As previously described, *Salmonella* spp. has the ability to colonize both pet animals and animals of livestock interest. Furthermore, these animals can act as asymptomatic carriers and can be a source of food contamination and, therefore, a risk to public health [43,44]. Other animals such as rodents, insects or reptiles can act as reservoirs and vectors for the transmission of Salmonella. Furthermore, in terms of a possible vector transmission, it has been observed that insects can act as carriers of *Salmonella* spp [45,46].

Food contaminated by *Salmonella* spp. can be of various types [47,48,49] so it is reasonable that any of them that are contaminated may have the potential to cause food poisoning. Classically, meat and eggs have been described as the main vehicles of human salmonellosis [42]; however, new foods (e.g., bison meat, papaya, blueberries, hazelnuts, kale, pepper, pistachios) capable of transmitting *Salmonella* spp. have been described recently [50].

The control of contamination by *Salmonella* spp. must be applied in all stages of the food chain “from farm to fork” where the control measures carried out in each stage are synergistic with other measures applied in the next phase of the food chain to guarantee its safety [51]. Salmonella detection methods in feed, food animals, the environment and foodstuffs have been essential tools to ensure its control throughout the food chain [52]. In the case of meat, contamination generally occurs on the surfaces of the carcass [53] through contact of carcass muscle by fecal material contaminated with *Salmonella* spp. during slaughter and/or cutting operations in the slaughterhouse.

Relative to the AMR of *Salmonella* spp., the EFSA report [54] indicates that a significant number of *Salmonella* spp. are resistant to the action of antibiotics commonly used in both human and veterinary medicine. High rates of *Salmonella* spp. isolated from human (Table 3) displayed an antimicrobial resistance prevalence of 25.3%, 26.8% and 26.2% to ampicillin, sulfonamides and tetracyclines, respectively. Also in human samples, the prevalence of susceptibility of *Salmonella* spp. was, on average, about 60.0%, with the highest proportion in *S. enteritidis* (72.1%), followed by *S. derby* (61.5%), *S. typhimurium* (58.9%) and *S. infantis* (54.5%). The lowest levels of susceptibility were observed in *S. kentucky* (19.4%) and monophasic *S. typhimurium* (4.5%). In human isolates reported from EU, multidrug resistance was still high (22.6%, n = 6867). Similar results have been recently reported (i.e., 2020) by the National Antimicrobial Resistance Monitoring System [55] of the FDA in which most of the salmonella (78%) isolated from humans displayed 100% of susceptibility to all antimicrobials included in the surveillance program. Antibiotic resistance of nontyphoidal salmonella infections increased in the last years achieving 8%, 3% and 1% for ciprofloxacin, ceftriaxone and azithromycin, respectively. Also, 74% of human isolates of *S. typhi* are resistant to ciprofloxacin [55].

In recent years, it has been suggested that one of the main causes of the increase in microbial resistance has been the abusive use of antibiotics in livestock production [56]. However, some recent studies cast doubt on this theory due to the difficulty of understanding transmission dynamics [57,58]. Also, the need of increasing of sales to maximize profits of the pharmaceutical industry may contribute to the persistence of AMR [59]. In addition, the transfer of antimicrobial resistance from meat to humans is a growing public health concern, especially in low- to middle-income countries with insufficient oversight on antimicrobial use.

Thus, the continued use of antibiotics in animal production both for the treatment and prevention of diseases enabling the transmission of resistant bacteria through the food chain is a cause for concern. The transfer of antimicrobial resistance from meat to humans has been related through the consumption of contaminated meat and/or meat products. This is because resistant bacteria present in the gastrointestinal tract of animals can persist in meat even after processing. In this way, it has been observed that horizontal gene transfer (i.e., exchange of resistance genes between bacteria) is a fundamental mechanism that facilitates the spread of antibiotic resistance [60,61]. However, other authors [62] question this way of transmission since the have observed that having livestock does not seem to contribute to the increase in AMR in humans.

Within the One Health context, antimicrobial resistance is continually transmitted between animals, humans and the environment. In this way, the fight against antibiotic resistance only in one of the three scenarios indicated above would be inadequate since each of them is influenced by the others. Animal health is inseparable from human health, so understanding resistance in samples from humans as well as its possible connection with animals is essential to develop and implement control strategies.

Strategies such as the responsible use of antibiotics in livestock production, implementation of electronic veterinary prescription in the EU, implementing biosafety measures to prevent the appearance of diseases and/or their spread, as well as establishing surveillance systems for resistant strains in both animals and humans, are essential. Thus, strict compliance with hygiene, cleaning and disinfection practices in the food production chain is essential to reduce or eliminate the risk of contamination [63,64,65].

In recent years, the media has indicated that antibiotic resistance is primarily due to livestock production, especially intensive farming. With the One Health perspective, this work aims to highlight that the resistance of *Salmonella* spp. is not only to antibiotics used in veterinary medicine but also to antibiotics used exclusively in human medicine. Since the transmission of AMR can occur through meat consumption, the objective of this study is to verify, through a meta-analysis, the prevalence of *Salmonella* spp. in the carcasses and/or organs of cattle, sheep, and goats, as well as their antimicrobial resistance (AMR). Furthermore, the role of *Salmonella* spp. (isolated in slaughterhouses) in the increase in the AMR microbial resistance of *Salmonella* spp. in humans is discussed in the text.

**Table 3:** Antimicrobial resistance of *Salmonella* spp. in humans by antimicrobial substance.

Antimicrobial	%	Country	Reference
Amikacin	3.40	Italy	[66]
	0.01	United States	[67]
	0.00	Central African Republic	[68]
Amoxicillin-clavulanic acid	40.00	Turkey	[69]
	22.50	United States	[70]
	8.10	United States	[71]
	7.69	Morocco	[72]
	5.20	Iran	[73]
	4.30	China	[74]
	3.12	Tunisia	[75]
	3.00	United States	[67]
	3.00	United States	[76]
	0.60	United States	[77]
	0.34	Central African Republic	[68]
	0.00	China	[78]
	0.00	Switzerland	[79]
Amoxicillin	27.80	Thailand	[80]
	6.87	Central African Republic	[68]
	0.00	Morocco	[72]
Ampicillin	100.00	Ethiopia	[81]
	92.16	China	[78]
	72.60	Italy	[82]
	73.00	Taiwan	[83]
	62.50	Tunisia	[75]
	60.00	Turkey	[69]
	57.50	Italy	[66]
	55.00	United States	[70]
	40.62	Switzerland	[79]
	40.30	Thailand	[84]
	39.99	Denmark	[83]
	33.33	India	[85]
	31.30	China	[74]
	29.80	Europe	[54]
	28.40	Spain	[86]
	25.00	Nigeria	[87]
	23.33	Nigeria	[87]
	22.40	Iran	[73]
	18.00	United States	[67]
	10.71	United States	[88]
	9.20	United States	[71]
	7.20	United States	[77]
	3.00	United States	[76]
	0.00	Brazil	[89]
Azithromycin	36.30	Italy	[66]
	1.96	China	[78]
	0.80	Europe	[54]
Aztreonam	8.60	Iran	[73]
Cefalotin	25.00	Switzerland	[79]
	4.00	United States	[67]
	3.00	United States	[76]
	3.60	United States	[77]
	3.20	Spain	[86]
	1.80	Iran	[73]
	0.17	Central African Republic	[68]
	0.00	Brazil	[89]
	0.00	Morocco	[72]
Cefepime	13.73	China	[78]
	7.40	India	[85]
	2.10	China	[74]
Cefixime	6.90	Iran	[73]
Cefoperazone	6.3	Thailand	[84]
Cefotaxime	77.77	India	[85]
	25.00	Nigeria	[87]
	20.00	Nigeria	[87]
	10.3	Taiwan	[83]
	4.10	Italy	[66]
	3.60	China	[74]
	3.40	Iran	[73]
	0.80	Europe	[54]
	0.10	Spain	[86]
	0.10	United States	[82]
	0.00	Turkey	[69]
	0.00	Central African Republic	[68]
	0.00	Denmark	[83]
	0.00	Switzerland	[79]
Cefoxitin	22.50	United States	[70]
	8.30	United States	[71]
	0.70	United States	[77]
	0.00	Central African Republic	[68]
Ceftazidine	17.65	China	[78]
	12.10	Iran	[73]
	8.60	Taiwan	[83]
	3.80	China	[74]
	3.40	Italy	[66]
	3.12	Tunisia	[75]
	0.80	Europe	[54]
	0.00	Morocco	[72]
	0.00	Denmark	[83]
Ceftiofur	22.50	United States	[70]
	8.20	United States	[71]
	6.40	China	[74]
	0.00	United States	[77]
Ceftriaxone	19.61	China	[78]
	11.11	India	[85]
	8.10	United States	[71]
	6.90	Iran	[73]
	5.00	United States	[70]
	3.00	United States	[76]
	1.00	United States	[67]
	0.00	United States	[77]
	0.00	Ethiopia	[81]
Ciprofloxacin	25.00	Nigeria	[87]
	14.81	India	[85]
	14.10	Europe	[54]
	10.10	United States	[71]
	8.90	Italy	[66]
	6.50	Thailand	[84]
	5.00	United States	[70]
	3.92	China	[78]
	3.33	Nigeria	[87]
	2.80	China	[74]
	1.80	Iran	[73]
	1.60	Taiwan	[83]
	1.56	Switzerland	[79]
	0.10	United States	[67]
	0.00	Brazil	[89]
	0.00	Thailand	[80]
	0.00	Ethiopia	[81]
	0.00	Morocco	[72]
	0.00	Spain	[86]
	0.00	United States	[82]
	0.00	Turkey	[69]
	0.00	United States	[77]
	0.00	Central African Republic	[68]
	0.00	Denmark	[83]
Chloramphenicol	48.10	Taiwan	[83]
	42.00	United States	[70]
	38.8	Thailand	[84]
	35.29	China	[78]
	29.90	United States	[82]
	20.40	Thailand	[80]
	17.20	Iran	[73]
	15.70	China	[74]
	14.20	Spain	[86]
	11.11	India	[85]
	10.10	Denmark	[83]
	10.00	United States	[67]
	9.60	Italy	[66]
	9.60	United States	[71]
	6.40	Europe	[54]
	5.85	Central African Republic	[68]
	3.12	Tunisia	[75]
	2.90	United States	[77]
	1.00	United states	[76]
	1.56	Switzerland	[79]
	0.00	Morocco	[72]
	0.00	Ethiopia	[81]
	0.00	Brazil	[89]
Colistin	2.70	Italy	[66]
Doxycycline	77.50	Iran	[73]
Furazolidone	3.12	Tunisia	[75]
Gentamicin	62.96	India	[85]
	33.70	Thailand	[84]
	24.30	Taiwan	[83]
	15.20	China	[74]
	12.50	Nigeria	[87]
	12.50	Tunisia	[75]
	10.00	Nigeria	[87]
	9.60	United States	[71]
	6.90	Iran	[73]
	5.60	Thailand	[80]
	5.00	United States	[70]
	3.00	United States	[76]
	3.00	United States	[82]
	3.00	United States	[67]
	2.60	Spain	[86]
	1.60	Europe	[54]
	1.10	United States	[77]
	0.60	Denmark	[83]
	0.17	Central African Republic	[68]
	0.00	Italy	[66]
	0.00	Morocco	[72]
	0.00	Ethiopia	[81]
	0.00	Brazil	[89]
	0.00	Turkey	[69]
	0.00	Switzerland	[79]
Kanamycin	33.33	Ethiopia	[81]
	22.40	Iran	[73]
	14.20	United States	[71]
	9.37	Tunisia	[75]
	5.00	United State	[67]
	3.12	Switzerland	[79]
	3.10	United States	[77]
	3.00	United States	[70]
	3.00	United States	[76]
	2.10	United States	[82]
	1.60	Spain	[86]
	0.00	Morocco	[72]
Imipenem	0.00	China	[78]
	0.00	Central African Republic	[68]
	0.00	Denmark	[83]
	0.00	Taiwan	[83]
	0.00	Iran	[73]
Levofloxacin	0.00	China	[78]
Piperacin/tazobactam	3.33	Nigeria	[87]
	0.00	China	[78]
Nalidixic acid	74.10	Iran	[73]
	70.31	Switzerland	[79]
	50.90	China	[74]
	35.00	Spain	[86]
	23.33	Nigeria	[87]
	21.60	Taiwan	[83]
	20.00	United States	[70]
	18.75	Tunisia	[75]
	13.10	Europe	[54]
	10.71	United States	[88]
	11.30	United States	[71]
	9.80	United States	[82]
	8.20	Italy	[66]
	6.25	Nigeria	[87]
	4.40	Denmark	[83]
	1.00	United States	[67]
	0.50	United States	[77]
	0.34	Central African Republic	[68]
	0.00	Morocco	[72]
	0.00	Brazil	[89]
Meropenem	10.00	Nigeria	[87]
	0.00	Italy	[66]
	0.00	Europe	[54]
Neomycin	12.50	Tunisia	[75]
Nitrofurantoin	64.70	Brazil	[89]
	33.30	Ethiopia	[81]
	30.00	Nigeria	[87]
	6.25	Nigeria	[87]
Ofloxacin	1.40	China	[74]
Ticarcillin	6.87	Central African Republic	[68]
Tigecycline	0.20	Europe	[54]
Tetracycline	92.60	Thailand	[80]
	85.40	Taiwan	[83]
	85.18	India	[85]
	80.00	United States	[70]
	76.40	United States	[82]
	68.75	Switzerland	[79]
	67.50	Thailand	[84]
	66.66	Ethiopia	[81]
	50.70	Italy	[66]
	46.66	Nigeria	[87]
	43.00	Nigeria	[87]
	40.00	Turkey	[69]
	37.30	Denmark	[83]
	31.20	Europe	[54]
	30.50	China	[74]
	27.30	Spain	[86]
	21.00	United States	[67]
	12.50	Tunisia	[75]
	11.80	Brazil	[89]
	11.53	Morocco	[72]
	10.71	United States	[88]
	9.90	United States	[77]
	5.00	United States	[76]
	3.51	Central African Republic	[68]
Norfloxacin	0.00	Thailand	[80]
	0.00	Brazil	[89]
Trimethoprim	62.96	India	[85]
	59.70	Thailand	[84]
	19.70	China	[74]
	6.20	Italy	[66]
	6.10	Europe	[54]
	5.90	Brazil	[89]
	3.12	Switzerland	[79]
Trim-sulpha	35.29	China	[78]
	31.50	Thailand	[80]
	20.00	United States	[70]
	19.70	China	[74]
	12.30	United States	[82]
	10.40	United States	[71]
	9.60	Europe	[54]
	7.61	Central African Republic	[68]
	7.00	Turkey	[69]
	6.00	Spain	[86]
	2.00	United States	[67]
	3.12	Tunisia	[75]
	0.60	United States	[77]
Tigecycline	2.70	Italy	[66]
Spectinomycin	48.00	Thailand	[84]
Streptomycin	100.00	Thailand	[80]
	100.00	Ethiopia	[81]
	87.00	Thailand	[84]
	81.60	Taiwan	[83]
	70.30	United States	[82]
	62.50	Switzerland	[79]
	42.50	United States	[70]
	40.50	Denmark	[83]
	37.60	China	[74]
	19.50	Spain	[86]
	19.00	United States	[67]
	10.00	United States	[76]
	12.40	United States	[77]
	10.71	United States	[88]
	8.80	United States	[71]
	3.84	Morocco	[72]
	3.12	Tunisia	[75]
	0.00	Brazil	[89]
Sulfamethoxazole	100	Italy	[66]
	100	Brazil	[80]
	96.10	Thailand	[84]
	89.20	Taiwan	[83]
	43.00	Denmark	[83]
	30.10	Europe	[54]
	20.00	United States	[67]
	10.00	United States	[77]
	9.37	Switzerland	[79]
	5.00	United states	[76]
Sulfisoxazole	70.00	United States	[70]
	50.00	United States	[88]
	47.90	China	[74]
	8.40	United States	[71]
Sulfonamide	88.20	Brazil	[89]
	75.40	United States	[82]
	22.30	Spain	[86]
	3.84	Morocco	[72]

## 4. Material and Methods

### 4.1. Search Strategy

**Figure 1:**
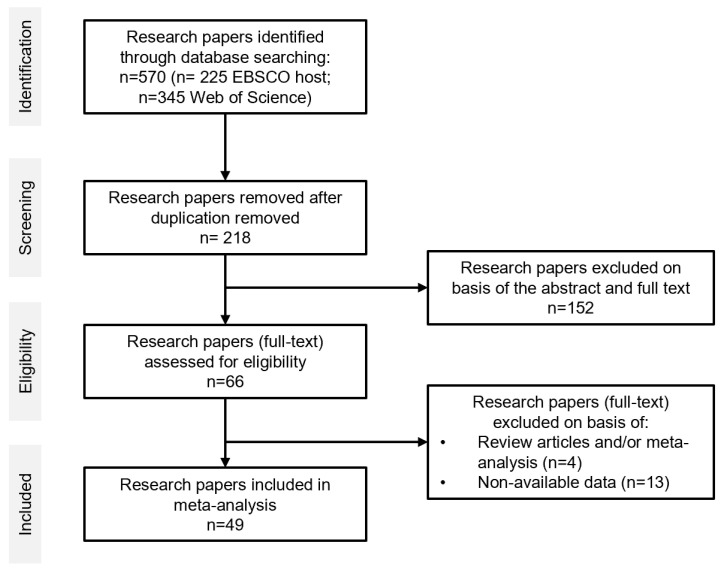
PRISMA flowchart for the selection process of eligible studies.

The systematic review was performed in accordance with the PRISMA (Preferred Reporting Items for Systematic Reviews and Meta-Analysis) guidelines [90,91] (Figure 1). Databases such as Google Scholar, Scopus, and PubMed were utilized to retrieve research papers in both English and Spanish. The article search commenced in September 2022 and concluded in April 2023. Scientific articles should include information on the presence/absence of *Salmonella* spp. isolated from beef, sheep, and goat carcasses and/or organs, as well as indicate the resistance against various antimicrobials.

The search for scientific articles was carried out using boolean terms in the EBSCOhost and directly in the browser bar of the aforementioned databases. The boolean terms used to identify the relevant scientific articles were “antimicrobial” AND “resistance” AND “slaughterhouse” OR “abattoir” OR “slaughter” AND “bovine” OR “sheep” OR “goat” AND “Salmonella”.

### 4.2. Inclusion and Exclusion Criteria

To conduct the meta-analysis, all research articles reporting the AMR of *Salmonella* spp. in slaughtered cattle, goats and/or sheep were selected. All selected publications were evaluated by all authors to determine their suitability for inclusion in the analysis. The inclusion criteria for the articles involved verifying the resistance to any antimicrobial by *Salmonella* spp. isolated from any part of the carcass and/or organ of cattle, goats, and/or sheep during slaughter. Publications related to systematic reviews and meta-analyses were not considered for the study. Articles studying the AMR of *Salmonella* spp. in the slaughterhouse environment, handlers, and/or slaughterhouse wastewater were also excluded. Those scientific papers related to the AMR of *Salmonella* spp. isolated from farms were not considered for the meta-analysis. Differences of AMR of *Salmonella* spp. among countries were not considered since production methods (intensive or extensive), type of production (milk, meat, or mixed), types of antibiotics used, or differences between serotypes have not been evaluated.

### 4.3. Review of the Scientific Literature and Data Analysis

The search for scientific articles was carried out from September 2022 to April 2023 (8-month period). Potentially eligible studies were selected in two steps; the first step was based on the title and abstract screening that must contain specific words as previously indicated in the heading. Irrelevant references were removed. The second step was based on the full-text reading of potentially relevant studies. For each reference, the following variables were systematically extracted and entered into a summary table: (1) author, (2) year of publication, (3) specie, (4) country of publication, (5) total samples, (6) total *Salmonella* spp. positive samples, (7) location of samples, (8) sampling location, (9) name of the antimicrobial substance tested and (10) number of *Salmonella* spp. samples resistant to each specific antimicrobial. All manuscript included in the meta-analysis, including data regarding *Salmonella* spp. prevalence and antimicrobial resistance evaluation, are presented in Supplementary Materials (Supplementary Materials S1 and S2). To improve the homogeneity of the studies included in the meta-analysis, the outcomes used were obtained similarly among them. Thus, isolation and identification of *Salmonella* spp., was carried out by classical microbiological and molecular techniques (e.g., PCR), respectively, while antimicrobial susceptibility testing was carried out in most studies by CLSI or EUCAST protocols. The assessing of the risk of bias in the included studies was based on a compliance chart of the points 1 to 9 previously described. All studies were carefully screened. If an article did not provide clear information at any point, then the scientific study was rejected for inclusion in the meta-analysis.

### 4.4. Meta-Analysis and Statistical Analysis

The effect size index for this meta-analysis was the prevalence of resistance of *Salmonella* spp. against specific antimicrobials. The effect size index is the prevalence itself, which is the magnitude that is intended to be synthesized from the estimates provided by the primary studies. The numerator value always refers to the total number of *Salmonella* spp. resistant to a specific antimicrobial, and the denominator refers to the total number of *Salmonella* spp. samples tested against the same antimicrobial used in the numerator. Thus, a value less than 0.5 indicates 100% of sensitivity of *Salmonella* spp. to a specific antimicrobial, while a value of 1 indicates 100% of resistance of *Salmonella* spp. to the antimicrobial used in the numerator.

For the statistical treatment, prevalence regarding antimicrobial resistance of *Salmonella* spp. extracted from each study was subjected to a Logit transformation to have a more symmetrical distribution that asymptotically approaches the normal distribution. Then, reported results of the prevalence of the antimicrobial resistance of *Salmonella* spp. and those indicated in the forest plot were transformed back to prevalence values [92].

Heterogeneity between studies was evaluated using the Q statistic as a test of heterogeneity, and the I^2^ statistic [93]. For the pooled estimate, the values were weighted by the inverse of their variances. Random effects models were assumed instead of the fixed-effect model [94]. Random effects models are generally preferred because they are more conservative and allow generalizing conclusions beyond the specific set of studies analyzed [95]. The specific variance (τ^2^) was estimated using the restricted maximum likelihood method. In order to test for a potential moderating role of the method employed when testing the antimicrobial resistance, we have adjusted a model that included the method (Disk Diffusion versus Broth Microdilution). The Q_between_ statistic allows testing for any role of a categorical moderator [94]. This test was not possible for a number of antimicrobials as the total number of studies or the number of studies in one of the methods was too small. All statistical analyses were performed through the metafor R package^®^ (v. 4.4.0) [96].

Based on the prevalence of the AMR of *Salmonella* spp. isolated from cattle, goat and sheep at slaughterhouses against the different antimicrobials, they are classified into three categories: low (≤5), high (>5–10≥) and very (high > 10).

## 5. Results

### 5.1. Salmonella spp. Prevalence in Slaughtered Large and Small Ruminants

Based on the 49 reviewed studies, the prevalence of *Salmonella* spp. domestic ruminants at slaughterhouses was 8.01% (Table 4). The prevalence of *Salmonella* spp. in cattle, goats, and sheep was 8.31%, 7.04%, and 6.12%, respectively (Table 4). On average, 708 animals were sampled at the slaughterhouses. Carcass, fecal content, and lymph nodes were analyzed in 71.42%, 46.93%, and 28.57% of the studies reviewed, respectively. The meta-analysis showed prevalence results of *Salmonella* spp. (Table 5) ranging from 8.31% to 7.04%, depending on the sampling location. However, due to insufficient studies, no prevalence results were obtained from the gallbladder, kidneys, lungs, rumen, and spleen. Forest plots and funnel plots of overall prevalence of *Salmonella* spp. at slaughterhouse, by specie and by sample location is presented in Supplementary Materials (Supplementary Materials S3–S6).

The studies included in the meta-analysis were conducted in 21 different countries worldwide, with nearly 50% carried out in Africa. The publication dates of the studies reviewed ranged from 2002 to 2022 (see Supplementary Material S1). No statistical differences were observed by country or year of publication.

### 5.2. Antimicrobial Resistance of Salmonella spp. from Slaughtered Domestic Ruminants

The antimicrobial resistance was assessed for 62 different antimicrobials. Twenty-two of them were not assessed by meta-analysis due to a lack of enough data (only 1 sample) and/or the *Salmonella* spp. displayed 100% sensitivity to the antimicrobial analyzed. To enhance reader comprehension, a full list of the antibiotics evaluated, classified by families, is presented in Table 6. Also, antimicrobial resistance of *Salmonella* spp. by antimicrobial substance of each manuscript included in the meta-analysis is presented in Supplementary Materials (Supplementary Materials S7 and S8).

The average antimicrobial resistance (AMR) by antimicrobial families was as follows: glycopeptides (96.77%), tetracyclines (34.89%), sulfonamides (22.55%), cephalosporins (18.97%), aminoglycosides (15.48%), penicillins (18.26% without cloxacillin), nitrofurans (13.74%), chloramphenicol (8.02%), quinolones (5.47% without orbifloxacin), macrolides (1.60%), lincosamides (1.72% without lincomycin), carbapenems (1.01%), oxazolidinones (0.00%), and phosphonic antimicrobials (0.00%). According to the AMR displayed by *Salmonella* spp., 27 antimicrobials (43.54%) were classified as low, 16 of then (20.80%) as high and 19 of then (30.64%) as very high (Table 7).

By individual antimicrobial, the classification of *Salmonella* spp. AMR is presented in Table 8. *Salmonella* spp. did not display AMR against aztreonam, mezlocillin, ertapenem, meropenem, cefoxitin, ceftazidime, levofloxacin, tilmicosin, linezolid, fosfomycin, furazolidone, quinupristin, trimethoprim and spectinomycin. In contrast, a prevalence of AMR of 100% has been described against ofloxacin, lincomycin, and cloxacillin. The antimicrobial netilmicin was not considered for classification since its prevalence in two studies was 0% and 100%.

According to the AMR of *Salmonella* spp., 27, 16, and 19 antimicrobials were classified as low, high, and very high, respectively. By antimicrobial families, *Salmonella* spp. presented very high AMR against antimicrobials belonging to cephalosporins, sulfonamides, and penicillins. However, absence of resistance of *Salmonella* spp. was observed against antimicrobials belonging to carbapenems and most antimicrobial macrolides, beta-lactams, and those included in the group “others”.

Regarding the methodology to determine the antimicrobial resistance, studies included in the meta-analysis used two techniques: disk diffusion assay and broth microdilution. The influence of the methodology of antimicrobial testing is presented in Table 9 and was not statistically significant for most antimicrobial substances studied. From 62 antimicrobials analyzed, ciprofloxacin (*p* < 0.01), gentamicin (*p* < 0.01), kanamycin (*p* < 0.05), trimethoprim-sulfamethoxazole (*p* < 0.05) and streptomycin (*p* < 0.05), *Salmonella* spp. displayed higher resistance prevalence when antimicrobial testing is based on the disk diffusion assay.

## 6. Discussion

In Europe, salmonellosis is the second most reported foodborne gastrointestinal infection in humans after campylobacteriosis and is a major cause of foodborne outbreaks. Poultry and pig meat are the main sources of salmonellosis according to animal species [42]. The reported prevalence of *Salmonella* spp. by EFSA was 0.003%, 0.03%, 0.08%, and 0.05% in chickens, turkeys, pigs, and cattle, respectively [42]. Since AMR is considered a significant health issue [97], the European Antimicrobial Resistance Surveillance Network (EARS-net) coordinated by the European Center for Disease Prevention and Control (ECDC) carries out the surveillance of bacterial pathogens commonly causing infections in humans. EARS-net integrates surveillance data on routine clinical antimicrobial susceptibility from clinical laboratories of member-states regarding *Escherichia coli*, *Klebsiella pneumoniae*, *Pseudomonas aeruginosa*, *Acinetobacter species*, *Streptococcus pneumoniae*, *Staphylococcus aureus*, *Enterococcus faecalis*, and *Enterococcus faecium* [98]. However, the surveillance of AMR of *Salmonella* spp. is carried out by EFSA and not by the ECDC [54]. Monitoring of *Salmonella* spp. is compulsory for broilers, fattening chickens, and pigs, and AMR characterization is carried out on these samples.

Regarding cattle, the EFSA report only presented data from fecal samples in slaughtered calves less than 1 year old from selected member-states [54]. The performance of the present meta-analysis on antimicrobial resistance has the advantage that the methodologies used to determine resistance/susceptibility are similar in all studies, whether through the Clinical and Laboratory Standards Institute (CLSI) [99] or the European Committee on Antimicrobial Susceptibility Testing (EUCAST) protocols [100]. However, some difficulties were encountered in evaluating the AMR of *Salmonella* spp. from cattle, goats, and sheep and their potential impact as a threat to public health. Among them, the scarce existing literature stands out, mainly carried out on farms, using fecal samples, and not in slaughterhouses. Furthermore, there is a high variety in the sample size as well as in the evaluated antibiotics.

Regarding small ruminants, the scarce research available could also be associated with the lower economic importance of sheep and goat production, which leads to veterinary pharmaceutics not developing and approving new antimicrobials. Although the variety of veterinary antimicrobial drugs for sheep and goat practice is narrow, the prevalence of AMR observed is still high, probably due to the extra-label use of antimicrobial drugs approved for cattle [101]. However, antimicrobial resistance in sheep needs to be further studied, as other research suggests not only a low prevalence but also limited resistance to antibiotics such as amoxicillin–clavulanic acid, ampicillin, azithromycin, cefoxitin, ceftiofur, ceftriaxone, chloramphenicol, ciprofloxacin, gentamicin, kanamycin, nalidixic acid, sulfisoxazole, or trimethoprim–sulfamethoxazole [102,103,104].

Traditionally, antimicrobial resistance has been associated with the abusive and/or inappropriate use of antibiotics, which leads to treatment failure and, ultimately, the death of animals. However, the effectiveness of antibiotic treatments can be influenced by external factors such as the season [105], the type of livestock production (extensive versus intensive, with low prevalence of AMR in the former) [106], the existence of pre-existing diseases. in animals [107], stress, the period of time elapsed from the onset of symptoms to the start of treatment, the immunological status of the animal or the impossibility of achieving therapeutic concentrations of the antibiotic at the site of infection. In addition, other factors associated with the management of animals, such as adequate nutrition, absence of vaccine prophylaxis programs or the presence of concomitant diseases [108], can also affect the effectiveness of antibiotic treatment [109]. Moreover, the sampling location on the at farm appears to influence the AMR of *Salmonella* spp. [110].

The prevalence of *Salmonella* spp. in cattle, sheep and goats, according to the result of the meta-analysis, it was approximately 8%, similar to other published results [111]. Although the latest EFSA report regarding the AMR of *Salmonella* spp. [54] indicates lower values of 0.81%, 2.1% and 1.2% for cattle, goats and sheep, respectively, on the contrary, other works have indicated higher prevalences of *Salmonella* spp. in sheep 43% (tonsils), 32.2% and 2.8% [102,112,113]. This variation in the prevalence of *Salmonella* spp. in sheep may be related to *S. enterica* subsp. *diarizonae*, which is endemic in sheep in some countries. It is host-adapted to sheep and does not cause infections in humans [114,115].

In addition, prevalence values of *Salmonella* spp. of 9.3% and 2% (tonsils) have been published in goats [102,113]. Although it is difficult to explain these differences in prevalence, factors such as hygiene at slaughter, implementation of Hazard Analysis and Critical Control Point (HACCP) plans, cleaning of animals before slaughter, the hygiene of handlers and the sampling location may influence these results. Furthermore, factors such as type of production (meat or dairy), age or health status (i.e., healthy or sick) of the animals also appear to influence *Salmonella* spp. concentrations in carcasses [116].

Relative to the AMR of *Salmonella* spp. in calves, the EFSA report [54] only shows results for 14 antibiotics. According to this report, *Salmonella* spp. is completely susceptible to gentamicin, cefoxitin, ceftazidime, meropenem and azithromycin. In our study, the results were the same for meropenem and similar for cefotaxime and azithromycin, with a prevalence of AMR less than 3% [54]. We also observed a similar AMR for ampicillin, sulfamethoxazole, tetracycline, and tigecycline.

Aminoglycosides, which include the antibiotics gentamicin, amikacin, tobramycin, neomycin, kanamycin or netilmicin among others, are broad-spectrum bactericidal antibiotics whose mechanism of action consists of the interruption of protein synthesis by irreversibly binding to the 16S ribosomal RNA receptor in the 30S subunit of the bacterial ribosome [117]. Relative to gentamicin, the results of the meta-analysis show a prevalence of AMR of *Salmonella* spp. eight times higher than that indicated in the EFSA report [54]. Furthermore, other studies have indicated higher resistance values from 10% [118,119,120,121,122,123,124,125] to 30% [126,127,128,129] for gentamicin in cattle.

While other studies [129] indicate that the resistance of *Salmonella* spp. in goat and sheep carcasses to gentamicin is almost complete, other studies [102] indicated complete total susceptibility in tonsil samples from sheep at the slaughterhouse. Gentamicin is used in cattle to treat septicemia and infections of the gastrointestinal tract, urogenital tract, and skin caused by Gram-negative bacteria. Traditionally, gentamicin has been used for the treatment of neonatal diarrhea in calves. The difference in resistance observed between the EFSA report and our meta-analysis can be explained by the fact that gentamicin can cause nephrotoxic effects in calves since their renal function is not fully mature. Due to the pharmacokinetics of gentamicin in calves, several administrations are necessary to achieve bactericidal concentrations, which, together with the dehydration caused by diarrhea, increases the risk of renal failure. Currently, there are other safer and more effective antibiotic treatments, so the use of gentamicin in calves is rare, which may explain the absence of resistance of *Salmonella* spp. to this antibiotic [130].

The resistance of *Salmonella* spp. against amikacin has been associated with the presence of the *aacA4* gene [108]. The low prevalence of resistance observed in slaughterhouses [124,127,131] is similar to studies carried out on farms [105,132,133], although prevalences of resistance of *Salmonella* spp. to amikacin of up to 16% have been reported [134]. The low emergence of resistance can be attributed to the type of commercial presentation of amikacin, only available in the EU for the treatment of mastitis in cattle.

The resistance of *Salmonella* spp. to kanamycin, according to the results of the meta-analysis (approximately 25%), is similar to other investigations [105], where resistance has been associated with the presence of O-adenyltransferase genes, such as the *aadb* gene [116]. The fact that kanamycin is only marketed as an antimastitis agent in the EU, it contributes to preventing the increase in its resistance [135]. Although, in some EU countries, injectable kanamycin is licensed for use in horses.

The information available on the resistance of *Salmonella* spp. to neomycin and streptomycin is limited. Published works indicate that the prevalence of resistance to these antibiotics is high, mainly in dairy cattle [122,123,124,125,126,127,128,129,130,131,132,133,134,135,136].

The prevalence of resistance of *Salmonella* spp. to tobramycin is moderate [135], although its use as a veterinary medicine drug in the EU is prohibited. This resistance to tobramycin may be explained by the existence of genes encoding resistance to gentamicin, which display co-resistance to tobramycin [137].

Since data published by EFSA on antimicrobial resistance indicate high resistance to first-line antibiotics for the treatment of diarrhea in calves, the use of aminoglycosides may be a therapeutic option in the treatment of neonatal diarrhea when Enterobacteriaceae remain susceptible [138].

Resistance to the antibiotics ampicillin, sulfonamides, and tetracyclines presented very high prevalence in samples of *Salmonella* spp. isolated from humans in 2019–2020 [54], while the prevalence of resistance of *Salmonella* spp. isolated from animals was moderate. The resistance of *Salmonella* spp. to these antibiotics was higher both in the EFSA report and in the results of our meta-analysis (22% and 31.8%, respectively). Other studies [139,140] have also indicated high resistance of *Salmonella* spp. to ampicillin (17.28%) in domestic ruminants. Furthermore, in other studies [102,104] resistance values of 95% and 100%, respectively, were observed in *S. enterica* subsp. *diarizonae*, isolated both on farms and in slaughterhouses, against sulfamethoxazole in sheep.

Ampicillin is a broad-spectrum penicillin antibiotic commonly used in ruminant veterinary clinics for the treatment of respiratory infections, mastitis, and gastrointestinal infections, including salmonellosis and *E. coli* infections (including diarrhea in calves) [141]. The observed high resistance may be associated with the presence of genes encoding extended-spectrum β-lactamases (ESBLs) that confer resistance to a wide range of β-lactam antibiotics, including ampicillin [125,142]. Furthermore, the high resistance of *Salmonella* spp. to this antibiotic may be associated with its widespread use, since penicillin antibiotics represent a third of all antibiotics marketed in Europe, while their consumption in other continents, such as Africa, represents less than 1% [143]. The AMR of *Salmonella* spp. to ampicillin of 40%, observed in our meta-analysis, suggests a high prevalence of ESBL [144]. Thus, 100% resistance of *Salmonella* spp. to ampicillin has been described in dairy cows [81]. However, no resistance was observed in sheep [102].

Amoxicillin is a broad-spectrum semisynthetic penicillin with bactericidal properties similar to those of ampicillin. Clavulanic acid has the function of preventing the inactivation of amoxicillin by irreversibly binding to beta-lactamases, protecting it from enzymatic inactivation [145].

The resistance of *Salmonella* spp. to amoxicillin/clavulanic acid is significant. Some studies indicate resistance values lower than 10% [102,121,123,146], while in others, *Salmonella* spp. has a resistance greater than 50% [147,148,149]. The resistance of *Salmonella* spp. to amoxicillin-clavulanic acid seems similar in both farms and slaughterhouses [132,133,134,150,151]. This difference of resistance seems to be associated with the health status of the animals. In healthy cattle, resistance of *Salmonella* spp. is less than 10% while in sick animals, the resistance of *Salmonella* spp. to amoxicillin-clavulanic acid exceeds 50% [108].

Although the objective of clavulanic acid is to inhibit beta-lactamases, the high resistance values observed in the meta-analysis are worrying. This resistance seems to be associated with the production of extended-spectrum beta-lactamases (ESBL) not inhibited by clavulanic acid, such as *PSE-1*, *OXA-1* and *TEM-1* being encoded by *bla* resistance genes. [152]. Furthermore, the presence of antibiotic efflux pumps in *Salmonella* spp. also seems to contribute to the high resistance against amoxicillin-clavulanic acid [153].

Information on the resistance of *Salmonella* spp. to the antibiotics aztreonam, mecillinam and ticarcillin is scarce, probably due to their non-use in veterinary medicine. However, a resistance of *Salmonella* spp. up to 50% for mezlocillin has been described in farms [142]. With the aim of protecting public health, Commission Implementing Regulation 2022/1255 has prohibited the veterinary use of these antibiotics, being exclusive for the treatment of specific infections in human medicine [97]. Carbapenems are broad-spectrum β-lactam antibiotics with bactericidal properties, mainly used in infections caused by Enterobacteriaceae with *AmpC* and *ESBL* activity [154]. Although the resistance of *Salmonella* spp. observed in the meta-analysis is very low, there seems to be some concern about the increase in resistance to these antibiotics by Enterobacteriaceae (including *Salmonella* spp.) [155]. This resistance is mediated by carbapenemase genes, such as *blaKPC*, *blaVIM, blaIMP*, *blaNDM* and *blaOXA-48* [156], posing a major public health problem. The prohibition of the use of this type of antibiotics in veterinary medicine is due to the fact that these antibiotics are considered the last line of treatment in human medicine [97].

Amphenicols are broad-spectrum bacteriostatic antibiotics. They diffuse through the bacterial cell membrane, bind to the 50S subunit of the 70S ribosome, inhibit peptidyl transferase, and interfere with the successful binding of complete transfer RNA to the ribosome, consequently disrupting peptide formation and protein synthesis.

The resistance of *Salmonella* spp. to chloramphenicol according to the results of the meta-analysis (12.23%) was three times higher than the values indicated in the EFSA report [54]. This resistance has been associated with the presence of chloramphenicol acetyltransferase genes such as *catA* [116,157]. Resistance values of *Salmonella* spp. to chloramphenicol similar to the results of the EFSA report, between 1.6% and 9.0%, have been described in the literature [132,133,134,150]. However, when *Salmonella* spp. is isolated from sick animals, resistance ranges between 50% and 90% [108].

Florfenicol is a fluorinated molecule derived from chloramphenicol used mainly for the treatment of respiratory diseases in cattle and sheep. The resistance of *Salmonella* spp. to florfenicol, both on farms and in slaughterhouses, is about 35% [137,158,159,160]. Resistance to florfenicol has been associated with the presence of the *FloR* gene [137]. Although the presence of the *FloR* gene does not seem to be the only factor that contributes to resistance, it has been observed [161] that 11% of *Salmonella* spp. isolated from cadavers presented resistance to florfenicol in the absence of *FloR* genes.

Sulfonamides are a class of antibiotics widely used in veterinary medicine, including ruminants. Its mechanism of action is based on the inhibition of the bacterial enzyme responsible for producing folic acid, which is essential for the growth and survival of bacteria. Sulfonamides are generally used for the treatment of respiratory, urinary and/or gastrointestinal infections. The high resistance of *Salmonella* spp. to sulfonamides observed in the meta-analysis is in accordance as previously reported [54,162]. However, combinations with trimethoprim have shown greater sensitivity of *Salmonella* spp. [163]. The resistance of *Salmonella* spp. to sulfonamides is associated with the presence of the genes *sul1*, *sul2* and *sul3* [164], which are located on plasmids [165]. Furthermore, it appears that the resistance of *Salmonella* spp. to sulfonamides varies depending on the season of the year [166].

Tetracyclines are one of the most used drugs in veterinary medicine. They have a broad spectrum acting against Gram-positive and Gram-negative bacteria, both aerobic and anaerobic. Its mechanism of action is based on the inhibition of bacterial protein synthesis through its binding to the 30S ribosomal subunit of bacteria. Resistance to tetracyclines is high, not only in the slaughterhouse but also on the farm [105,107,160,167,168], although resistance of *Salmonella* spp., isolated from sheep, has not been published [102,104]. The efficacy of tetracyclines has been decreasing in previous decades due to the widespread existence of resistance genes, probably associated with the prolonged and extensive use of these antimicrobials in humans and as growth promoters in animals [169]. Resistance to tetracyclines has been associated with the presence of *tet* genes. Thus, it was observed that in 98% of the cases in which *Salmonella* spp. presented resistance to tetracyclines, almost all of the isolates had the *tetA* gene [160]. However, other authors [161] found that 66% of *Salmonella* spp. isolated from carcasses were sensitive although they carried *tetG* genes. Thus, the presence of this gene does not seem to be an essential factor for the development of resistance. Doxycycline is a second-generation tetracycline family antibiotic. It is mainly used for the treatment of bovine respiratory syndrome in fattening calves. The resistance of *Salmonella* spp. to doxycycline is low both in the slaughterhouse and in primary production [137]. However, there are studies that show an increase in resistance of up to 35% in primary production [150,170].

Tigecycline is a broad-spectrum antibiotic used in serious infections. The prohibition of its use in veterinary medicine [99] may explain the low resistance of *Salmonella* spp. to this antibiotic [54,171]. The use of tigecycline aims to avoid resistance to tetracycline, although some studies have reported the existence of resistance genes to this antibiotic (e.g., *tet(a)*, *tet(K)*, *tet(M)*, *tet(x)*) representing an emerging concern in public health [172]. However, the studies on the resistance of *Salmonella* spp. to this antimicrobial must be carefully analyzed since there are no specific clinical breakpoints for *Salmonella* spp.

Macrolides are a family of antibiotics widely used in veterinary medicine against Gram-negative bacteria, whose antimicrobial action is based on the inhibition of protein synthesis. The resistance of *Salmonella* spp. to azithromycin (reference antibiotic for the treatment of salmonellosis in human medicine and not marketed for use in livestock) was moderate. Variable levels of azithromycin resistance in *Salmonella* spp. have been observed in both cattle and sheep [54,127,131], being associated with the presence of the genes *mph(A)*, *erm(42)*, *erm(B)* and possibly a greater expression of efflux pumps due to *ramAp* genes or defective *ramR* [173].

Relative to colistin, increased resistance by *Salmonella* spp. may be associated with its greater use in swine production for the treatment of intestinal problems as in medicated feed [174]. In cattle and small ruminants, colistin is also authorized in the EU for the treatment of diarrhea caused by *E. coli* or *Salmonella* spp., although for these species parenteral treatment with quinolones or sulfonamides is much more common, which may explain the low resistance [175,176]. Furthermore, colistin resistance genes are mainly found on plasmids, so their horizontal transmission to other bacteria is currently a public health concern.

Erythromycin and tilmicosin are antibiotics authorized in the EU for use in cattle and small ruminants for the treatment of respiratory diseases caused by *Mannheimia haemolytica* and *Pasteurella multocida*, as well as for the treatment of interdigital dermatitis caused by *D. nodosus*. Although it is difficult to explain the low resistance observed in the meta-analysis, it can be attributed to the low number of references included in the meta-analysis. In addition, a decrease in its use is in favor of other antibiotics such as ceftiofur (with a broader spectrum of action although with higher levels of resistance) for the treatment of claw and respiratory problems with a shorter withdrawal period. However, some studies have detected a high resistance of *Salmonella* spp. to erythromycin and tilmicosin both in cattle farms and in slaughtered sheep [102,177,178]. Since resistance against these seem to be high according to the literature, the results should be carefully interpreted and monitoring measures should be implemented to verify their potential impact on public health.

Lincosamides act against Gram-positive bacteria by inhibiting protein synthesis.

Clindamycin is mainly used in veterinary medicine for companion animals, which may explain the low resistance observed in the meta-analysis [122]. However, resistance values greater than 30% have been described in *Salmonella* spp. [177,179].

In the case of lincomycin, the only publication found indicated resistance of *Salmonella* spp. of 100%. However, this result should be interpreted cautiously since lincomycin is generally used in swine production in the form of medicated feed. Its resistance has been associated with the presence of the *Cfr* gene that encodes unusual multidrug resistance, including resistance to lincomycin. This gene has been found in several species, such as staphylococci, enterococci and *E. coli* from animals intended for human consumption [74,180]. Since this gene is located on the plasmid, its transfer to other bacteria, including *Salmonella* spp., may pose a public health risk [74].

Enterobacteriaceae (i.e., *Salmonella* spp.) are intrinsically resistant to macrolide and lincosamide antimicrobials (with the exception of azithromycin) [181]. Additionally, in vitro susceptibility testing for macrolide and lincosamide antimicrobials is problematic since there are no clinical breakpoints for Salmonella concerning lincosamides or erythromycin [182]. Thus, reports on the low prevalence [122,175,176] of macrolide and lincosamide resistance must be carefully interpreted.

Cephalosporins are bactericidal antibiotics against Gram-positive and Gram-negative bacteria which interfere with peptidoglycan synthesis as well as inactivate endogenous autolysin inhibitors. Autolysin is responsible for breaking down bacterial cell walls, causing bacterial death by lysis [183]. Cephalosporins are antibiotics widely used in veterinary medicine for the treatment of bovine respiratory syndrome, metritis and pododermatitis. However, sales of cephalosporins for use in livestock represent less than 4% [184]. Currently, the use of 1st and 2nd generation cephalosporins should be used with caution, while 3rd and 4th generation cephalosporins should be used only if no alternative antibiotic therapy exists in categories C and/or D [185]. Relative to 1st generation cephalosporins (cephalothin and cefazolin), the resistance of *Salmonella* spp. is variable. Some studies indicate a high sensitivity of *Salmonella* spp. to cephalothin both in cattle [162,186] and in small ruminants [187]. However, more recent studies have reported high resistance of *Salmonella* spp. isolated in slaughterhouses [122]. Regarding cefazolin, the low resistance (e.g., less than 10%) of *Salmonella* spp. observed in slaughterhouses are in accordance with what has been published elsewhere [188].

In general, the resistance of *Salmonella* spp. to 2nd generation cephalosporins is low. In the case of cefoxitin, *Salmonella* spp. presents variable resistance values, from 12% [105] or 40% [189] to 100% [190] in dairy farms. Furthermore, the resistance of *Salmonella* spp. to cefoxitin in calves with diarrhea is 8% [141]. For cefaclor, no other bibliographic references have been found; however, given that the genes for resistance to cephalosporins are located in the plasmids, and considering the high resistance found in other enterobacteria, the high resistance observed may be due to the acquisition of resistance genes [191].

The resistance of *Salmonella* spp. to 3rd generation cephalosporins is also low. Resistance to ceftriaxone varies between 10% and 30% [105,107,122], although more recent studies indicate lower resistance values around 4% [190,192].

Regarding ceftazidime, the absence of resistance observed in the meta-analysis is in accordance as reported in the literature [141], although resistance values of 25% have been reported [136]. In the case of cefixime, higher resistance values of *Salmonella* spp. on livestock farms have been published [189,193].

With respect to ceftiofur, a 3rd generation cephalosporin authorized only for veterinary use, the resistance of *Salmonella* spp. observed in slaughterhouses is low, and probably associated with a low prevalence of the *blacmy-2* gene [110]. The resistance of *Salmonella* spp. to ceftiofur has progressively decreased in recent years [105] to values of around 20% in dairy farms. Although, in cattle farms with cases of salmonellosis, the resistance of *Salmonella* spp. to ceftiofur reaches 50% [194]. Although some studies have indicated that ceftiofur use contributes to the increase in antimicrobial resistance [195] through horizontal transfer of *blaCMY-2* genes, other authors suggest that this increase is also influenced by environmental factors [196]. Furthermore, treatments with ceftiofur appear to decrease the prevalence of *Salmonella* spp. in cattle; however, they exert selective pressure for more resistant strains [194] with greater persistence over time [196].

Resistance to cephalosporins is mediated by class A enzymes called ESBLs, which include three families encoded by *TEM*, *SHV* and *CTX-M* type genes in Enterobacteriaceae. These enzymes confer resistance to 1st, 2nd and 3rd generation cephalosporins. In addition, some Gram-negative bacteria have genes encoding class C enzymes (*cAmpC* and *pAmpC*) located on plasmids, which can be easily transmitted horizontally between Enterobacteriaceae, including *Salmonella* spp. This class of enzymes is encoded by genes such as *CMY*, *FOX*, *LAT*, *MIR*, *ACT*, *DHA*, *ACC*, and *MOX*, which, unlike class A enzymes, confer resistance to cephalosporins, with the exception of 4th generation cephalosporins [197,198]. Thus, increased resistance to cephalosporins, especially 3rd generation cephalosporins, poses a risk to public health [199].

Fosfomycin is a broad-spectrum antibiotic that, due to increasing antibiotic resistance, has been suggested for use in veterinary medicine mainly for infections resistant to 3rd generation cephalosporins [200]. However, its current use in veterinary medicine is completely prohibited [97]. Studies on the resistance of *Salmonella* spp. to fosfomycin are scarce. The absence of resistance observed in the meta-analysis is in accordance with the results obtained in dairy cattle farms [167,201]. Furthermore, it has been described that 27% of *Salmonella* spp. isolated from carcasses and organs carried the *fosA7.7* resistance gene to fosfomycin, which represents a risk to public health since this antibiotic is considered critically important for the treatment of human infections [201].

Nitrofuran antibiotics act by blocking protein synthesis, breaking DNA chains and inhibiting the activity of acetyl-coenzyme A. The results of the meta-analysis show that the resistance of *Salmonella* spp. to these antibiotics is greater than 20%. The result obtained is much higher than the resistance rate of less than 5% to furazolidone and nitrofurantoin in *Salmonella* spp. isolated in farms [136,202,203,204]. The higher rate of resistance observed in slaughterhouses is difficult to explain since both antibiotics are not marketed for veterinary use. Some authors [205] have indicated that the presence of the plasmid-mediated *OqxAB* gene in *E. coli* contributes to resistance to nitrofurantoin. Because this gene is located on plasmids, the acquisition of resistance by *Salmonella* spp. horizontally could explain the greater resistance observed in the slaughterhouse.

Spectinomycin, a bacteriostatic antibiotic active against certain Gram-negative aerobic bacteria, Gram-positive cocci and *Mycoplasma* spp., is used in ruminant medicine for both the treatment of respiratory and digestive problems. Although the results of the meta-analysis indicate 100% susceptibility to *Salmonella* spp., other authors reported high resistance rates in both adult cattle [177,206] and calves [207,208].

Trimethoprim shows antimicrobial activity against several species of Gram-negative bacteria by inhibition of dihydrofolate reductase, interfering in the synthesis of bacterial nucleic acids and proteins. The resistance of *Salmonella* spp. in the slaughterhouse observed in the meta-analysis seems to be low although these results should be interpreted with caution since resistance rates between 20% and 50% have been reported in cattle and small ruminants, respectively [209,210]. Furthermore, the available studies on the resistance of *Salmonella* spp. to trimethoprim is scarce since it is marketed in combination with a sulfonamide.

Quinolones are broad-spectrum bactericidal antibiotics that act by inhibiting the synthesis of the DNA by altering the enzyme DNA gyrase [211]. In livestock medicine, the most common quinolones are enrofloxacin and marbofloxacin that are used for the treatment of neonatal diarrhea in calves, mastitis and respiratory problems. Although sales of quinolones represent only 3% of all antibiotics, an increase in resistance associated with mutations of the *gyrA*, *gyrB*, *parC* and *parE* genes has been verified [212]. As a result, its use is considered restricted to specific cases [185].

Relative to ciprofloxacin, the resistance of *Salmonella* spp. ranges between 0.5% and 2%, slightly lower than results observed in the meta-analysis [108,110,160,213,214,215,216]. Furthermore, no resistance has been reported in sheep [102,104]. The resistance of *Salmonella* spp. to nalidixic acid is higher than ciprofloxacin in dairy farm samples, varying from complete susceptibility to 40% of resistance [102,104,108,201,214,217]. This increase in resistance may be due to the fact that mutations in topoisomerases that confer resistance to nalidixic acid are more common than mutations that confer resistance to ciprofloxacin [218]. The resistance of *Salmonella* spp. to ofloxacin found in the slaughterhouse is 100%, although other authors have reported similar resistance values to ciprofloxacin, ranging from 0% to 11% [215,219]. Therefore, the results of the meta-analysis should be considered with caution due to the scarce literature on antimicrobial resistance of *Salmonella* spp. to ofloxacin. Although ofloxacin is marketed for veterinary medicine, it is not approved for use in ruminants. Studies on the resistance of *Salmonella* spp. against enrofloxacin and norfloxacin are very rare, showing resistance values similar to those observed in the meta-analysis for both cattle and sheep [220,221].

From the perspective of One Health, and according to the data obtained in the systematic review, it was observed that studies about the resistance of *Salmonella* spp. to different antibiotics are much more extensive in samples of *Salmonella* spp. isolated from animals than from human samples. In our meta-analysis, information about the resistance of *Salmonella* spp. was obtained for a total of 58 antibiotics. In contrast, the systematic review displayed information about the resistance of *Salmonella* spp. from human samples for a total of 26 antibiotics.

Additionally, when comparing the antibiotics indicated in the systematic review for the treatment of salmonellosis in humans and animals, only three antibiotics (ampicillin, chloramphenicol, and trimethoprim-sulfamethoxazole) are recommended for salmonellosis treatment. In the case of ampicillin and chloramphenicol, both antibiotics show high resistance values in isolates of *Salmonella* spp. from both ruminants and humans, while in the case of trimethoprim-sulfamethoxazole, resistance is twice as high in human isolates.

If we engage in a more detailed discussion of the antibiotics in Table 10, most antibiotics licensed for use in veterinary medicine by the European Medicine Agency (not only for salmonellosis treatment) show similar AMR values for both *Salmonella* spp. isolated from animals and humans (gentamicin, neomycin, streptomycin, ceftiofur, ampicillin). Only kanamycin and colistin exhibit values three and two times higher, respectively, in *Salmonella* spp. isolated from animals than in human isolates.

Regarding the specific antimicrobials used to treat salmonellosis in humans, the AMR of azithromycin against *Salmonella* spp. (not licensed for veterinary use) is 4.5 times higher in human isolates. Concerning quinolones, most antimicrobial substances analyzed in the meta-analysis exhibited higher AMR values against *Salmonella* spp. isolated from ruminants than those isolated from humans. However, none of them (with the exception of enrofloxacin, exclusively used in veterinary medicine) are used, licensed, or commercialized for ruminant medicine or other food-producing animals. Also, the resistance pattern of *Salmonella* spp. against quinolones are in accordance with [222].

With regards to cephalosporins, most antimicrobials displayed higher AMR values against *Salmonella* spp. isolated from human samples, similarly as seen in the case of quinolones. In addition, for those antimicrobials that presented higher AMR values against *Salmonella* spp. from ruminant isolates, the antimicrobial substances are not approved for veterinary use. Furthermore, ceftiofur (an exclusively veterinary use-only 3rd gen. cephalosporin) showed slightly higher AMR resistance against *Salmonella* spp. in human-isolated samples than in animal-isolated samples.

Although several publications suggest that the increase in AMR is related to the misuse and/or abusive use of antimicrobials in animal production [61], the results of the meta-analysis, as well as data obtained from the systematic review, suggest that the AMR transmission dynamics from ruminants to humans are not entirely clear. This is because resistance of *Salmonella* spp., for both animal and human isolates, to most antimicrobial substances studied, displayed similar values or even higher resistance in isolates from human samples for common veterinary antibiotics used in ruminant medicine, such as ceftiofur, trimethoprim-sulfa, or oxytetracycline [223]. Additionally, the higher resistance of *Salmonella* spp. (both from human and/or ruminant samples) against antimicrobials described in the meta-analysis, used exclusively in human medicine, suggests that the global AMR concern could be related to the misuse and/or abusive use of antimicrobials in human medicine and not exclusively to their misuse and/or abusive use in animal production. Also, the reduction of AMR in humans and livestock must be part of a One Health approach [223,224,225]. Due to the lack of studies about the role of large and small ruminants in the antimicrobial resistance of *Salmonella* spp., regular surveillance of antimicrobial resistance through global targeted studies and systematic meta-analyses may improve knowledge on this topic [226].

## 7. Conclusions

The prevalence of *Salmonella* spp. in large and small ruminants in slaughterhouses according to the meta-analysis is 8.01% (8.31% in cattle; 7.04% in goats; 6.12% in sheep). The prevalence of resistance of *Salmonella* spp., depending on the family of antibiotics, is as follows: 96.77%—glycopeptides, 34.89%—tetracyclines, 22.55%—sulfonamides, 18.97%—cephalosporins, 15.48%—aminoglycosides, 14.02%—penicillins, 13.74%—nitrofurans, 8.02%—chloramphenicol, 5.47%—quinolones, 1.60%—macrolides, 1.72%—lincosamides, and 1.01%—carbapenems.

According to the resistance rates of *Salmonella* spp., 20, 14 and 13 antibiotics were classified as low (≤5%), high (>5% ≤10%) and very high (>10%), respectively. Although the resistance of *Salmonella* spp. indicates some degree of concern, most antibiotics are not used in veterinary medicine. This highlights that the increasing rates of antibiotic resistance in *Salmonella* spp. are not only associated with the use of antibiotics in veterinary practice but also with other factors such as the immunological status of the animal, the presence or absence of concomitant diseases, farm management, environmental factors and/or the irrational use of antibiotics in veterinary practice by humans. Thus, further research about AMR of *Salmonella* spp. considering these aspects (i.e., immunological status, presence of specific diseases) may be necessary.

From the One Health perspective, both the results of the meta-analysis and the systematic review indicate that the resistance of *Salmonella* spp., whether isolated from ruminant or human samples, to different antibiotics is, in most cases, similar. The contribution of cattle, sheep and goats to the rise of antibiotic resistance of *Salmonella* spp. and its potential impact on public health appears to be relatively insignificant, due to their low prevalence in carcasses and organs.

However, it is important to highlight that the available research literature on antimicrobial resistance of *Salmonella* spp. isolated from animals to different antibiotics is very limited, mainly in sheep and goats, and mostly come from to low- to middle-income countries with insufficient oversight on antimicrobial use.

## Figures and Tables

**Table 4:** Prevalence of *Salmonella* spp. in slaughtered cattle and small ruminant.

	K	Prev. (%)	Tau^2^	I^2^ (%)	Q	C.I. (95%)
Overall	49	8.01	1.962	98.69	2651.969	5.29–11.52
Cattle	39	8.31	1.85	98.68	2144.36	5.54–12.29
Goats	5	7.04	2.39	96.19	42.318	1.83–23.61
Sheep	5	8.31	4.19	97.64	185.142	1.14–41.31

Prev.: prevalence, C.I.: confidence interval.

**Table 5:** Prevalence of *Salmonella* spp. in slaughtered large and small ruminants by sampling location.

	Stud. ^a^	Stud ^b^	Prev. ^c^	Tau^2^	I^2^ (%)	Q	C.I. (95%)
Carcass	35	33	4.60	2.43	98.38	2267.348	2.71–7.66
Feces	23	22	3.73	3.77	99.24	1772.599	1.63–8.06
Int. mucosa	4	3	8.37	0.46	96.83	58.266	4.00–16.63
Kidney	3	0	-	-	-	-	-
Liver	8	4	1.65	3.20	91.97	53.892	0.26–9.55
Lungs	2	0	-	-	-	-	-
LN	14	-	3.95	4.57	98.72	466.521	1.24–11.83
Rumen	3	0	-	-	-	-	-
Spleen	5	0	-	-	-	-	-

^a^ Total (%) studies reviewed. ^b^ Total studies considered for meta-analysis. ^c^ Prevalence of *Salmonella* spp. in slaughtered large and small ruminants from studies included in the meta-analysis.

**Table 6:** Classification of antimicrobials studied by families.

Aminoglycosides		Amphenicols		Carbapenems		Cephalosporins		Quinolones	
	Amikacin		Florfenicol		Ertapenem		Cefaclor		Ciprofloxacin
	Gentamicin		Chloramphenicol		Imipenem		Cephalothin		Enrofloxacin
	Kanamycin				Meropenem		Cefazolin		Levofloxacin
	Neomycin						Cefixime		Nalidixic acid
	Netilmicin						Cefotaxime		Norfloxacin
	Tobramycin						Cefoxitin		Ofloxacin
	Streptomycin						Ceftiofur		
							Ceftriaxone		
							Ceftazidime		
							Cefuroxime		
Lincosamides		Macrolides		Oxazolidinona		Penicillins		Phosphonic antm.	
	Clindamycin		Azithromycin		Linezolid		Amoxicillin		Fosfomycin
	Lincomycin		Erythromycin				Amox-clav		
			Tilmicosin				Ampicillin		
							Aztreonam		
							Cloxacillin		
							Mecillinam		
							Mezlocillin		
							Ticarcillin		
							Piperacillin		
Sulfonamides		Tetracyclines		Nitrofurans		Others			
	Trim-sulpha		Doxycycline		Furazolidone		Colistin		
	Sulfisoxazole		Tetracycline		Nitrofurantoin		Polymyxin		
	Sulphamtx						Quinupristin		
	Sulfonamide						Trimethoprim		
							Tigecycline		
							Spectinomycin		

Amox-clav: amoxicillin-clavulanic acid; sulphamtx: sulphametoxazole; antm.: antimicrobial.

**Table 7:** Classification * of antimicrobials (%) according to the resistance of *Salmonella* spp. analyzed by meta-analysis as low (≤5), high (>5–10≥) and very (high > 10).

Low		High		Very High	
*Amphenicols*		*Amynoglycosides*		*Aminoglycosides*	
	Florfenicol		Amikacin		Kanamycin
*Carbapenems*			Gentamicin		Streptomycin
	Ertapenem		Neomycin	*Cephalosporins*	
	Imipenem		Tobramycin		Cefixime
	Meropenem	*Cephalosporins*			Cephalothin
*Cephalosporins*			Cefoxitin	*Amphenicols*	
	Cefazolin		Ceftriaxone		Chloramphenicol
	Cefotaxime		Ceftiofur	Quinolones	
	Ceftazidime	*Quinolones*			Ofloxacin
*Lincosamides * ^A^			Ciprofloxacin	*Penicillin*	
	-		Enrofloxacin		Amox-Clav
*Macrolides * ^A^			Nalidixic acid		Amoxicillin
	Azithromycin		Norfloxacin		Ampicillin
*Oxazolidinone*		*Penicillins*		*Sulfonamides*	
	Linezolid		Piperacillin		Trim-sulpha
Penicillins		*Other*			Sulfisoxazole
	Mellicinam		Colistin	*Tetracyclines*	
	Mezlocillin		Polymyxin		Tetracycline
	Ticarcillin			*Nitrofuran*	
*Tetracyclines*					Nitrofurantoin
	Doxycycline				
*Nitrofurans*					
	Furazolidone				
*Others*					
	Trimethoprim				
	Tigecycline				
	Spectinomycin				

Amox-Clav: amoxicillin and clavulanic acid; Trim-sulpha: trimethoprim-sulfamethoxazole. * The following antimicrobials: aztreonam, cefaclor, cefuroxime, cloxacillin, fosfomycin, levofloxa-cin, lincomycin, quinupristin, sulfamethoxazole and sulfonamide, with only one reference in the metanalysis, were not included in the classification. ^A^ Lincosamides (e.g., clindamycin) and macrolides (e.g., erythromycin, tilmicosin) antimicrobials, with the exception of azithromycin, are not presented since they are considered intrinsically resistant to *Salmonella* spp.

**Table 8:** Prevalence (%) of antimicrobial resistance of *Salmonella* spp. from domestic ruminants at slaughterhouse.

Parte Superior do Formulário								
Antimicrobial Family		N	Prev. (%)	Tau^2^	I^2^ (%)	Q	C.I. (95%)	*p*
*Aminoglycosides*								
	Amikacin	13	5.06	1.1986	56.69	28.997	2.19–11.36	*p * < 0.01
	Gentamicin	41	8.62	2.1485	84.03	138.962	5.17–14.12	*p * < 0.001
	Kanamycin	22	23.86	2.8203	93.92	195.623	12.47–40.68	*p * < 0.001
	Neomycin	7	7.24	4.1856	81.87	34.289	1.37–30.34	*p * < 0.001
	Tobramycin	5	7.24	2.446	64.03	11.480	0.61–16.56	*p * < 0.05
	Streptomycin	34	40.87	3.1341	95.95	491.214	26.38–57.15	*p * < 0.001
*Amphenicols*								
	Florfenicol	10	3.80	2.901	75.22	40.454	1.03–12.92	*p * < 0.001
	Chloramphenicol	39	12.23	2.6235	91.43	270.663	7.10–20.26	*p * < 0.001
*Carbapenems*								
	Ertapenem	4	0	-	-	-	-	-
	Imipenem	7	1.01	0	0	1.409	0.35–2.85	ns
	Meropenem	3	0	-	-	-	-	-
*Cephalosporins*								
	Cefaclor	1	61.53	-	-	-	-	-
	Cephalothin	5	22.79	1.9548	96.26	112.840	7.58–51.67	*p * < 0.001
	Cefazolin	5	2.10	1.7956	61.15	12.680	0.45–9.34	*p * < 0.01
	Cefixime	4	75.21	2.2453	66.37	10.676	32.47–95.06	*p * < 0.05
	Cefotaxime	14	2.73	1.8477	61.49	50.123	1.03–7.05	*p * < 0.001
	Cefoxitin	21	5.84	3.8066	86.98	118.054	2.31–13.99	*p * < 0.001
	Ceftiofur	3	7.24	7.3732	95.32	27.839	65.18	*p * < 0.001
	Ceftriaxone	22	6.47	3.8475	91.74	127.765	2.61–15.21	*p * < 0.001
	Ceftazidime	7	0.00	-	-	-	-	-
	Cefuroxime	1	5.88	-	-	-	-	-
*Quinolones*								
	Ciprofloxacin	36	5.89	2.6675	85.68	156.243	3.15–10.70	*p * < 0.001
	Enrofloxacin	13	9.97	3.7761	85.55	47.663	3.06–27.84	*p * < 0.001
	Levofloxacin	1	0.00					
	Nalidixic acid	28	8.62	3.4502	92.70	507.717	4.15–16.98	*p * < 0.001
	Norfloxacin	12	6.53	1.5325	74.29	35.969	2.76–14.77	*p * < 0.001
	Ofloxacin	6	100	-	-	-	-	-
*Lincosamides*								
	Clindamycin	8	1.72	3.6211	75.87	54.922	0.35–8.01	*p * < 0.001
	Lincomycin	1	100	-	-	-	-	-
*Macrolides*								
	Azithromycin	11	2.73	3.2313	80.50	55.446	0.77–9.18	*p * < 0.001
	Erythromycin	5	2.08	8.2394	85.18	41.647	0.13–25.12	*p * < 0.001
	Tilmicosin	3	0	-	-	-	-	-
*Oxazolidinone*								
	Linezolid	4	0.00	-	-	-	-	-
*Penicillins*								
	Amoxicillin	11	14.55	5.6126	90.69	66.508	3.52–44.32	*p * < 0.001
	Amox-clav	27	16.11	4.6991	96.03	254.694	7.28–31.90	*p * < 0.001
	Ampicillin	42	30.49	2.8935	94.27	456.560	19.76–43.83	*p * < 0.001
	Aztreonam	1	0	-	-	-	-	-
	Cloxacillin	1	100	-	-	-	-	-
	Mecillinam	4	3.13	0.1372	9.86	2.749	1.17–8.02	ns
	Mezlocillin	3	0	-	-	-	-	-
	Penicillin	8	4.74	9.2684	81.51	37.311	0.47–34.02	*p * < 0.001
	Piperacillin	7	6.23	1.885	68.84	17.462	1.71–20.10	*p * < 0.01
	Ticarcillin	4	2.23	3.1391	74.31	15.818	0.28–15.43	*p * < 0.001
*Phosphonic antm*.								
	Fosfomycin	1	0	-	-	-	-	-
*Sulfonamides*								
	Trim-sulpha	29	13.70	2.8992	96.18	312.112	7.55–23.59	*p * < 0.001
	Sulfisoxazole	14	16.90	2.1318	93.49	94.230	7.95–32.30	*p * < 0.001
	Sulfamethoxazole	1	41.94	-	-	-	-	-
	Sulfonamide	1	17.67	-	-	-	-	-
*Tetracyclines*								
	Doxycycline	4	2.46	3.7076	72.86	13.848	0.26–19.21	*p * < 0.01
	Tetracycline	42	32.43	2.2847	94.60	400.293	22.25–4457	*p * < 0.001
*Nitrofurans*								
	Furazolidone	3	0	-	-	-	-	-
	Nitrofurantoin	13	27.48	4.3785	94.3	85.483	9.93–56.53	*p * < 0.001
*Others*								
	Colistin	11	5.95	6.5904	81.16	58.626	1.12–26.17	*p * < 0.001
	Polymyxin	3	8.86	9.0049	80.76	10.025	0.22–81.04	*p * < 0.01
	Quinupristin	1	0	-	-	-	-	-
	Trimethoprim	8	0	-	-	-	-	-
	Tigecycline	5	2.00	0.4398	19.85	4.364	0.53–6.98	ns
	Spectinomycin	4	0	-	-	-	-	-

antm.: antimicrobial; C.I.: confidence interval; Prev.: prevalence.

**Table 9:** Influence of the methodology (disk diffusion vs. broth microdilution) on the antimicrobial resistance of *Salmonella* spp. for each antimicrobial substance.

Antimicrobial Substance	Q_between_	*p*
Amikacin	0.2224	0.637
Amoxicillin	3.7010	0.054 ^A^
Amoxicillin–clavulanic acid	0.4758	0.4903
Ampicillin	1.5730	0.209
Azithromycin	0.5495	0.459
Aztreonam	-	- ^B^
Ciprofloxacin	6.6788	0.009 ^C^
Cefaclor	-	- ^B^
Cefepime	0.0389	0.8437
Cephalothin	0.5218	0.470
Cefazolin	0.5143	0.473
Cefixime	-	- ^D^
Cefotaxime	0.9410	0.332
Cefoxitin	2.4960	0.114
Ceftiofur	0.1048	0.746
Ceftriaxone	1.1128	0.291
Ceftazidime	0.1398	0.708
Cefuroxime	-	- ^B^
Clindamycin	0.2772	0.598
Colistin	1.0607	0.301
Chloramphenicol	1.5414	0.214
Cloxacillin	-	- ^B^
Dalfopristin	-	- ^B^
Doxycycline	-	- ^D^
Ertapenem	-	- ^D^
Erythromycin	-	- ^D^
Enrofloxacin	0.2229	0.636
Florfenicol	1.2040	0.272
Fosfomycin	-	- ^B^
Furazolidone	-	- ^D^
Gentamicin	9.2505	0.002 ^C^
Imipenem	0.5650	0.452
Kanamycin	3.8575	0.049 ^C^
Levofloxacin	-	- ^B^
Lincomycin	-	- ^B^
Linezolid	-	- ^D^
Mecillinam	-	- ^D^
Meropenem	-	- ^E^
Mezlocillin	-	- ^D^
Nalidixic acid	4.6581	0.030
Norfloxacin	0.1121	0.737
Neomycin	-	- ^D^
Netilmicin	-	- ^D^
Nitrofurantoin	0.0003	0.986
Ofloxacin	-	- ^B^
Penicillin	-	- ^D^
Piperacillin	0.2045	0.651
Polymyxin	0.3233	0.569
Quinupristin	-	- ^B^
Trimethoprim-sulfamethoxazole	4.5165	0.033 ^C^
Trimethoprim	0.0167	0.897
Tigecycline	0.1502	0.698
Ticarcillin	0.3210	0.571
Tobramycin	-	- ^D^
Tetracycline	1.4475	0.228
Tilmicosin	-	- ^D^
Spectinomycin	-	- ^D^
Streptomycin	4.8390	0.027 ^C^
Sulfixoxazole	0.0457	0.830
Sulfafurazole	-	- ^B^
Sulfamethoxazole	-	- ^B^
Sulfonamide	-	- ^B^

^A^: in the case of amoxicillin, *p*-value was 0.054. Although the *p*-value is not smaller than 0.05, this result can be considered as marginally significant, suggesting that the resistance of *Salmonella* spp. is probably higher in antimicrobial testing based on disk diffusion assay. ^B^: for aztreonam, cefaclor, cefuroxime, cloxacillin, dalfopristin, fosfomycin, levofloxacin, lincomycin, ofloxacin, quinupristin and sulfafurazole, no comparison among methods was possible since only one study was included in the meta-analysis. ^C^: in the case of ciprofloxacin (*p* = 0.0098), gentamicin (*p* = 0.0024), kanamycin (*p* = 0.0495), trimethoprim-sulfamethoxazole (*p* = 0.0336) and streptomycin (*p* = 0.0278), resistance of *Salmonella* spp. was higher when antimicrobial testing is based on the disk diffusion assay. ^D^: for cefixime, doxycycline, ertapenem, furazolidone, linezolid, mecillinam, mezlocillin, penicillin, tobramycin and tilmicosin, no comparison was possible since all studies included in the meta-analysis used the disk diffusion assay. ^E^: for meropenem, no comparison was possible since all studies included in the meta-analysis use the broth microdilution assay.

**Table 10:** Comparison of the antimicrobial resistance (AMR) of *Salmonella* spp. isolated from ruminants based on the results of the meta-analysis *(a)* and AMR of *Salmonella* spp. isolated from humans based on the results of the systematic review *(b)*. Results indicate the prevalence (%) of the AMR.

Antimicrobial Family		*(a)*	*(b)*	Observations
*Aminoglycosides*				
	Amikacin ^1,2,^*	5.06	1.13	Commercialized exclusively for horses
	Gentamicin ^1,2,^*	8.62	8.82	-
	Kanamycin ^1,2,^*	23.86	8.35	-
	Neomycin ^1,2,^*	7.24	12.5	-
	Netilmicin ^2^	-	-	-
	Tobramycin ^1^	7.24	-	Commercialized exclusively for cat and dogs
	Streptomycin ^1,2,^*	40.87	39.40	-
*Amphenicols *				
	Florfenicol ^1,^*	3.80	-	-
	Chloramphenicol ^1,2,^*	12.23	14.47	Not authorized in the EU for large and small ruminants
*Carbapenems *				
	Ertapenem ^2^	0.00	-	-
	Imipenem ^2^	1.01	0	-
	Meropenem ^2^	0.00	3.33	-
*Cephalosporins *				
	Cefaclor ^2^	61.53	-	-
	Cephalothin ^2,^*	22.79	4.53	-
	Cefazolin ^1,2^	2.10	-	-
	Cefixime ^2^	75.21	6.90	-
	Cefotaxime ^2^	2.73	5.84	-
	Cefoxitin ^2^		7.87	-
	Ceftiofur ^1,^*	7.24	9.27	-
	Ceftriaxone ^2^	6.47	6.08	-
	Ceftazidime ^2^	0.00	5.49	-
	Cefuroxime ^2^	5.88	-	-
	Cefepime ^2^	-	7.74	-
	Cefoperazone ^1,2^	-	6.3	-
*Quinolones *				
	Ciprofloxacin ^2^	5.89	4.14	-
	Enrofloxacin ^1,^*	9.97	-	-
	Levofloxacin ^2^	0.00	0.00	-
	Nalidixic acid ^2^	8.62	18.97	-
	Norfloxacin ^1,2^	6.53	0.00	Not authorized in the EU for large and small ruminants
	Ofloxacin ^1,2^	100	1.40	Not authorized in the EU for large and small ruminants
*Lincosamides *				
	Clindamycin ^1,2^	1.72	-	Commercialized exclusively for cat and dogs
	Lincomycin ^1,2^	100	-	-
*Macrolides *				
	Azithromycin ^2^	2.73	13.02	-
	Erythromycin ^1,2^	2.08	-	-
	Tilmicosin ^1,2^	0.00	-	-
*Oxazolidinone *				
	Linezolid ^2^	0.00	-	
*Penicillins *				
	Amoxicillin ^1,2^	14.55	-	-
	Amox-clav ^1,2,^*	16.11	-	-
	Ampicillin ^1,2,^*	30.49	38.97	-
	Aztreonam ^2^	0.00	8.60	-
	Mecillinam ^2^	3.13	-	-
	Cloxacillin ^1,2^	100	-	-
	Mezlocillin ^2^	0.00	-	-
	Piperacillin ^2^	6.23	-	-
	Ticarcillin ^2^	2.23	6.87	-
*Phosphonic antm. *				
	Fosfomycin ^2^	0	-	-
*Sulfonamides *				
	Tri-sulfa ^1,2,^*	13.70	14.19	-
	Sulfisoxazole ^2^	16.9	44.07	-
	Sulphamtx ^2^	41.94	-	-
	Sulfonamide ^2^	17.67	47.43	-
*Tetracyclines *				
	Doxycycline ^1,2^	2.46	-	-
	Tetracycline ^1,2,^*	32.43	42.29	-
*Nitrofurans *				
	Furazolidone ^1,2^	0.00	3.12	Not authorized in the EU for large and small ruminants
	Nitrofurantoin ^2^	27.48	33.56	-
*Others *				
	Colistin ^1,2^	5.95	2.70	-
	Polymyxin ^1,2^	8.86	-	Only authorized in the EU for sheep
	Quinupristin ^2^	0.00	-	-
	Trimethoprim ^1,2^	0.00	23.38	-
	Tigecycline ^2^	2.00	-	-
	Spectinomycin ^1,2^	0.00	48.00	-

* Described in the literature for the treatment of salmonellosis in large and small ruminants. ^1^ Antimicrobial licensed in the EU for veterinary use. ^2^ Antimicrobial licensed in the EU for human medicine use.

## Data Availability

Data are contained within the article and Supplementary Materials.

## Supplementary Materials

The following supporting information can be downloaded at: https://www.mdpi.com/article/10.3390/vetsci11070315/s1, Supplementary Material S1: Research papers included in the meta-analysis; Supplementary Material S2: PRISMA check list 2020; Supplementary Material S3: Data about *Salmonella* spp. prevalence from studies analyzed for meta-analysis; Supplementary Material S4. Forest plot *a)* and funnel plot *b)* of *Salmonella* spp. prevalence in slaughtered large and small ruminants; Supplementary Material S5. Forest and funnel plots of prevalence of *Salmonella* spp. by specie (a: cattle, b: goat, c: sheep); Supplementary Material S6. Forest and funnel plots of prevalence of *Salmonella* spp. by sample location. (a): carcass, (b): feces, (c): intestinal mucosa, (d): liver, (e): lymph nodes; Supplementary Material S7. Forest plots *(a)* and funnel plots *(b)* of antimicrobial resistance of *Salmonella* spp. by antimicrobial; Supplementary Material S8. Prevalence of antimicrobial resistance os *Salmonella* spp. by antimicrobial substance.
