# Supplementary material for: Salmonella spp. in Domestic Ruminants, Evaluation of Antimicrobial Resistance Based on the One Health Approach—A Systematic Review and Meta-Analysis
Source: Vet Sci. 2024 Jul 14;11(7):315. doi: 10.3390/vetsci11070315 (PMC11281391; doi:10.3390/vetsci11070315)
Supplement: Supplementary file 1 [file vetsci-11-00315-s001.zip › vetsci-3059985-supplementary.pdf]

Supplementary Material S1. Research papers included in the meta-analysis.

1. Abraham, R.; Sahibzada, S.; Jordan, D.; O'Dea, M.; Hampson, D.J.; McMillan, K., … Abraham, S. Antimicrobial resistance and genomic relationships of Salmonella enterica from Australian cattle. *Int J. Food Microbiol*. **2022**, *371*, 109672. https://doi.org/10.1016/j.ijfoodmicro.2022.109672
2. Alemu, A.; Regassa, F.; Kebede, N.; Ambachew, R.; Girma, M.; Asefa, Z.; Tsegaye, W. Magnitude and antimicrobial susceptibility profile of salmonella recovered from export abattoirs located in East Shewa, Ethiopia. *Infect. Drug Resis*. **2022**, 1353-1365. https://doi.org/10.2147/IDR.S348773
3. Alemu, S.; Zewde, B.M. Prevalence and antimicrobial resistance profiles of Salmonella enterica serovars isolated from slaughtered cattle in Bahir Dar, Ethiopia. *Trop. An. Health Produc*. **2012**, *44*, 595-600. https://doi.org/10.1007/s11250-011-9941-y
4. Al-Zubaid, AA.; Yousif, A.A. Prevalence and antimicrobial susceptibility of salmonella species isolate from slaughtered cows in Iraq. *Iraqi J. of Vet. Med*, **2013**, *37*(1), 96-101.
5. Amera, G.M.; Yirdaw, M.M.; Kibret, M. Antimicrobial resistance profile of salmonella species isolated from slaughtered cattle carcass and slaughterhouse environment in Dessie municipality abattoir, Ethiopia. *Abyssinia J. Sci. Tech*. **2017**, *2*(2), 30-37.
6. Atlaw, N.A.; Keelara, S.; Correa, M.; Foster, D.; Gebreyes, W.; Aidara-Kane, A.; … Fedorka-Cray, P.J. Evidence of sheep and abattoir environment as important reservoirs of multidrug resistant Salmonella and extended-spectrum beta-lactamase *Escherichia coli*. *Int. J. Food. Microbiol*. **2022**, *363*, 109516. https://doi.org/10.1016/j.ijfoodmicro.2021.109516
7. Bacon, R.T.; Sofos, J.N.; Belk, K.E.; Hyatt, D.R.; Smith, G.C. Prevalence and antibiotic susceptibility of Salmonella isolated from beef animal hides and carcasses. *J. Food Protect*. **2002**, *65*(2), 284-290. https://doi.org/10.4315/0362-028X-65.2.284
8. Barlow, R.S.; McMillan, K. E.; Duffy, L.L.; Fegan, N.; Jordan, D.; Mellor, G.E. Prevalence and antimicrobial resistance of Salmonella and *Escherichia coli* from Australian cattle populations at slaughter. *J. Food Protect*. **2015**, *78*(5), 912-920. https://doi.org/10.4315/0362-028X.JFP-14-476
9. Bier, D.; Kich, J.D.; Duarte, S.C.; Silva, M.R.; Valsoni, L.M.; Ramos, C.A.; … Araújo, F.R. Survey of *Salmonella* spp. in beef meat for export at slaughterhouses in Brazil. *Pes. Vet. Brasil*. **2018**, *38*, 2037-2043. https://doi.org/10.1590/1678-5150-PVB-5867
10. Cetin, E.; Serbetcioglu, T.; Temelli, S.; Eyigor, A. Nontyphoid Salmonella carriage, serovar profile and antimicrobial resistance phenotypes in slaughter cattle. *J. Food Safety*, **2019**, *39*(2), e12603. https://doi.org/10.1111/jfs.12603
11. Cetin, E.; Temelli, S.; Eyigor, A. Nontyphoid Salmonella prevalence, serovar distribution and antimicrobial resistance in slaughter sheep. *Food Sci. An. Res*. **2020**, *40*(1), 21. https://doi.org/10.5851%2Fkosfa.2019.e75
12. Edrington, T.S.; Long, M.; Ross, T.T.; Thomas, J.D.; Callaway, T.R.; Anderson, R.C.; … Nisbet, D.J. Prevalence and antimicrobial resistance profiles of *Escherichia coli* O157: H7 and Salmonella isolated from feedlot lambs. *J. Food Protec*. **2009**, *72*(8), 1713-1717. https://doi.org/10.4315/0362-028X-72.8.1713
13. Ekli, R.; Adzitey, F.; Huda, N. Prevalence of resistant Salmonella spp. isolated from raw meat and liver of cattle in the Wa Municipality of Ghana. In *IOP Conference Series: Earth and Environmental Science,* **2019***,* Vol. 287, No. 1, p. 012006). IOP Publishing. https://doir.org/10.1088/1755-1315/287/1/012006
14. Ferede, B.; Desissa, F.; Feleke, A.; Tadesse, G.; Moje, N. Prevalence and antimicrobial susceptibility of Salmonella isolates from apparently healthy slaughtered goats at Dire Dawa municipal abattoir, Eastern Ethiopia. *J. Microbiol. Antimicrob*. **2015**, *7*(1), 1-5. https://doi.org/10.5897/JMA2014.0331
15. Gabana, A.D.A.; Núncio, A.S.P.; Lopes, B.C.; de Oliveira, J.A.; da Silva Monteiro, L.; de Menezes Coppola, M.; … Mayer, F.Q. Different Multidrug-Resistant Salmonella spp. Serovars Isolated from Slaughter Calves in Southern Brazil. *Current Microbiol*. **2023***,* *80*(1), 11. https://doi.org/10.1007/s00284-022-03136-5
16. Geresu, M.A.; Desta, W.Z. Carriage, risk factors, and antimicrobial resistance patterns of Salmonella isolates from raw beef in Jimma, Southwestern Ethiopia. *Infect. Drug Resis*. **2021**, 2349-2360. https://doi.org/10.2147/IDR.S313485
17. Gragg, S.E.; Loneragan, G.H.; Nightingale, K.K.; Brichta-Harhay, D.M.; Ruiz, H.; Elder, J.R.; … Brashears, M.M. Substantial within-animal diversity of Salmonella isolates from lymph nodes, feces, and hides of cattle at slaughter. *App. Environ. Microbiol*. **2013**, *79*(15), 4744-4750. https://doi.org/10.1128/AEM.01020-13

18. Gutema, F.D.; Abdi, R.D.; Agga, G.E.; Firew, S.; Rasschaert, G.; Mattheus, W.; ... De Zutter, L. Assessment of beef carcass contamination with Salmonella and *E. coli* O 157 in slaughterhouses in Bishoftu, Ethiopia. *Int. J. Food Cont.* **2021**, *8*, 1-9. https://doi.org/10.1186/s40550-021-00082-1
19. Iglesias, M.A.; Kroning, I.S.; Decol, L.T.; de Melo Franco, B.D.G.; da Silva, W.P. Occurrence and phenotypic and molecular characterization of *Listeria monocytogenes* and *Salmonella* spp. in slaughterhouses in southern Brazil. *Food Res. Int.* **2017**, *100*, 96-101. https://doi.org/10.1016/j.foodres.2017.06.023
20. [(1)]Jajere, S.M.; Adamu, N.B.; Atsanda, N.N.; Onyilokwu, S.A.; Gashua, M.M.; Hambali, I.U.; Mustapha, F.B. Prevalence and antimicrobial resistance profiles of Salmonella isolates in apparently healthy slaughtered food animals at Maiduguri central abattoir, Nigeria. *As. Pac. J. Trop. Dis.* **2015**, *5*(12), 996-1000. https://doi.org/10.1016/S2222-1808(15)60971-9
21. Jitjak, T. Prevalence and antimicrobial resistance of Salmonella spp. isolated from fattening beef cattle at the slaughterhouse in Sakon Nakhon Province. *SNRU J. Sci. Tech.* **2016**, *8*(3), 338-343.
22. Kebede, A.; Kemal, J.; Alemayehu, H.; Habte Mariam, S. Isolation, identification, and antibiotic susceptibility testing of Salmonella from slaughtered bovines and ovines in Addis Ababa Abattoir Enterprise, Ethiopia: a cross-sectional study. *Int. J. Bacteriol.* **2016**. https://doi.org/10.1155/2016/3714785
23. Ketema, L.; Ketema, Z.; Kiflu, B.; Alemayehu, H.; Terefe, Y.; Ibrahim, M.; Eguale, T. Prevalence and antimicrobial susceptibility profile of Salmonella serovars isolated from slaughtered cattle in Addis Ababa, Ethiopia. *BioMed Res. Int.* **2018**. https://doi.org/10.1155/2018/9794869
24. Kore, K.; Asrade, B.; Demissie, K.; Aragaw, K. Characterization of Salmonella isolated from apparently healthy slaughtered cattle and retail beef in Hawassa, southern Ethiopia. *Prev. Vet. Med.* **2017**, *147*, 11-16. https://doi.org/10.1016/j.prevetmed.2017.08.018
25. Lynne, A.M.; Kaldhone, P.; David, D.; White, D.G.; Foley, S.L. Characterization of antimicrobial resistance in Salmonella enterica serotype Heidelberg isolated from food animals. *Foodborne Path. Dis.* **2009**, *6*(2), 207-215. https://doi.org/10.1089/fpd.2008.0172
26. Madoroba, E.; Gelaw, A.K.; Kapeta, D. Salmonella contamination, serovars and antimicrobial resistance profiles of cattle slaughtered in South Africa. *Onderstepoort J. Vet. Res.* **2016**, *83*(1), 1-8. https://hdl.handle.net/10520/EJC189737
27. Mathole, M.A.; Muchadeyi, F.C.; Mdladla, K.; Malatji, D.P.; Dzomba, E.F.; Madoroba, E. Presence, distribution, serotypes and antimicrobial resistance profiles of Salmonella among pigs, chickens and goats in South Africa. *Food Control*, **2017**, *72*, 219-224. https://doi.org/10.1016/j.foodcont.2016.05.006
28. McEvoy, J.M.; Doherty, A.M.; Sheridan, J.J.; Blair, I.S., McDowell, D.A. The prevalence of Salmonella spp. in bovine faecal, rumen and carcass samples at a commercial abattoir. *J. App. Microbiol.* **2003**, *94*(4), 693-700. https://doi.org/10.1046/j.1365-2672.2003.01898.x
29. Mustefa, B.A.; Gebremedhin, E.Z. Carriage and antimicrobial resistance of non-typhoidal Salmonella in cattle slaughtered in Ambo municipality abattoir, West Shewa zone, Oromia, Ethiopia-a point prevalence survey. *Ethio. Vet. J.* **2018**, *22*(2), 94-109. https://doi.org/10.4314/evj.v22i2.8
30. Nouichi, S.; Quatouat, R.; Can, H.; Mezali, L.; Belkader, C.; Ouar Korichi, M.; ... Hamdi, T. Prevalence and antimicrobial resistance of Salmonella isolated from bovine and ovine samples in slaughterhouses of Algiers, Algeria. *J. Hell. Vet. Med. Soc.* **2018**, *69(1)*, 863-872. http://dx.doi.org/10.12681/jhvms.16441
31. Obaidat, M.M. Prevalence and antimicrobial resistance of *Listeria monocytogenes*, *Salmonella enterica* and *Escherichia coli* O157: H7 in imported beef cattle in Jordan. *Comp. Immunol. Microbiol. Infect. Dis.* **2020**, *70*, 101447. https://doi.org/10.1016/j.cimid.2020.101447https://doi.org/10.1016/j.cimid.2020.101447
32. Oueslati, W.; Rjeibi, M.R.; Mhadhbi, M.; Jbeli, M.; Zrelli, S.; Ettriqui, A. Prevalence, virulence and antibiotic susceptibility of *Salmonella* spp. strains, isolated from beef in Greater Tunis (Tunisia). *Meat Sci.* **2016**. *119*, 154-159. https://doi.org/10.1016/j.meatsci.2016.04.037
33. Shaibu, A.O.; Okolocha, E.C.; Maikai, B.V.; Olufemi, O.T. Isolation and antibiogram of Salmonella species from slaughtered cattle and the processing environment in Abuja abattoirs, Nigeria. *Food Control*, **2021**, *125*, 107972. https://doi.org/10.1016/j.foodcont.2021.107972
34. Shilangale, R.; Kaaya, G.; Chimwamurombe, P. Antimicrobial resistance patterns of Salmonella strains isolated from beef in Namibia. *British Microbiol. Res. J.* **2016**, *12*(1), 1-6. https://doi.org/10.9734/BMRJ/2016/19775
35. Sibhat, B.; Molla Zewde, B.; Zerihun, A.; Muckle, A.; Cole, L.; Boerlin, P.; ... Gebreyes, W.A. Salmonella serovars and antimicrobial resistance profiles in beef cattle, slaughterhouse

personnel and slaughterhouse environment in Ethiopia. *Zoo. Pub. Health*, **2011**, *58*(2), 102-109. https://doi.org/10.1111/j.1863-2378.2009.01305.x
36. Stevens, A.; Kaboré, Y.; Perrier-Gros-Claude, J.D.; Millemann, Y.; Brisabois, A.; Catteau, M.; ... Dufour, B. Prevalence and antibiotic-resistance of Salmonella isolated from beef sampled from the slaughterhouse and from retailers in Dakar (Senegal). *Int. J. Food Microbiol.* **2006**, *110*(2), 178-186. https://doi.org/10.1016/j.ijfoodmicro.2006.04.018
37. Takele, S.; Woldemichael, K.; Gashaw, M.; Tassew, H.; Yohannes, M.; Abdissa, A. Prevalence and drug susceptibility pattern of Salmonella isolates from apparently healthy slaughter cattle and personnel working at the Jimma municipal abattoir, south-West Ethiopia. *Trop. Dis. Travel Med. Vacc.* **2018**, *4*, 1-7. https://doi.org/10.1186/s40794-018-0072-6
38. Varela-Guerrero, J.A.; Talavera-Rojas, M.; Gutiérrez-Castillo, A.D.C.; Reyes-Rodríguez, N.E.; Vázquez-Guadarrama, J. Phenotypic–genotypic resistance in Salmonella spp. isolated from cattle carcasses from the north central zone of the State of Mexico. *Trop. An. Health Prod.* **2013**, *45*, 995-1000. https://doi.org/10.1007/s11250-012-0323-x
39. Wabeto, W.; Abraham, Y.; Anjulo, A.A. Detection and identification of antimicrobial-resistant Salmonella in raw beef at Wolaita Sodo municipal abattoir, Southern Ethi. *J. Health Pop. Nut.* **2017**, *36*(1), 1-7. https://doi.org/10.1186/s41043-017-0131-z
40. Wang, J.; Xue, K.; Yi, P.; Zhu, X.; Peng, Q.; Wang, Z.; ... Aleri, J.W. An abattoir-based study on the prevalence of Salmonella fecal carriage and ESBL related antimicrobial resistance from culled adult dairy cows in Wuhan, China. *Pathogens*, **2020**, *9*(10), 853. https://doi.org/10.3390/pathogens9100853
41. Webb, H.E.; Brichta-Harhay, D.M.; Brashears, M.M.; Nightingale, K.K.; Arthur, T.M.; Bosilevac, J.M.; ... Loneragan, G.H. Salmonella in peripheral lymph nodes of healthy cattle at slaughter. *Front. Microbiol.* **2017**, *8*, 2214. https://doi.org/10.3389/fmicb.2017.02214
42. Wieczorek, K.; Osek, J. Prevalence and characterization of Salmonella in slaughtered cattle and beef in Poland. *J. Vet. Res.* **2013**, *57*(4), 607-611. https://doi.org/10.2478/bvip-2013-0103
43. Wottlin, L.R.; Edrington, T.S.; Anderson, R.C. Salmonella carriage in peripheral lymph nodes and feces of cattle at slaughter is affected by cattle type, region, and season. *Front. An. Sci.* **2022**, *3*, 859800. https://doi.org/10.3389/fanim.2022.859800
44. [(2)]Zare, P., Ghorbani, C.H.; Jaberi, S.; Razzaghi, S.; Mirzaei, M.; Mafuni, K. Occurrence and antimicrobial resistance of *Salmonella* spp. and *Escherichia coli* isolates in apparently healthy slaughtered cattle, sheep and goats in East Azarbaijan province. Int. J. Entric. Pathog. 2014, 2(1), e15451. https://doi.org/10.17795/ijep15451

(1) The research paper Jajere et al. (2015) has been included twice for meta-analysis as it includes data on the prevalence of antimicrobial resistance of *Salmonella* spp. in cattle and goats.

(2) The research paper Zare et al. (2014) has been reviewed three times for meta-analysis as it includes data on the prevalence of antimicrobial resistance of Salmonella spp. in cattle, goats, and sheep

Supplementary Material S2. PRISMA check list 2020

Section and topic	Item	Checklist item
**TITLE**		
Title	1	**Identify the report as a systematic review.**<br>The term "systematic review" is indicated in the title.
**ABSTRACT**		
Abstract	2	**See the PRISMA 2020 for Abstracts checklist.**<br>Checklist for abstract was added as supplementary material.
**INTRODUCTION**		
Rationale	3	**Describe the rationale for the review in the context of existing knowledge.**<br>The aim of the study was to verify the prevalence of *Salmonella* spp. resistance to different antibiotics isolated from the carcasses and/or organs of cattle, sheep, and goats. Additionally, the study analyzes, from a One Health perspective, the issue of *Salmonella* spp. resistance both in humans and animals, discussing the degree of resistance of this bacterium, to antibiotics used solely in human medicine, solely in veterinary medicine, or to those antibiotics commonly used in both animals and humans.
Objectives	4	**Provide an explicit statement of the objective(s) or question(s) the review addresses.**<br>The present work evaluates if antimicrobial resistance of Salmonella spp. isolated from cattle, sheep and/or goat carcass and/or organs at slaughterhouse represent a public health threat. This information is provided at the final part of the introduction section as follows: "*given that livestock production has been considered the main cause of antimicrobial resistance whose transmission occurs through the ingestion of contaminated meat, the objective of this study is to verify, through meta-analysis, the prevalence of Salmonella spp. in carcasses and/or organs of cattle, sheep, and goats as well as their AMR. Furthermore, the role of Salmonella spp. (isolated in slaughterhouses) in the increase of the AMR microbial resistance of Salmonella spp. in humans is discussed in the text.*"
**METHODS**		
Eligibility criteria	5	**Specify the inclusion and exclusion criteria for the review and how studies were grouped for the syntheses.**<br>Eligibility (inclusion and exclusion) criteria is described in heading 4.2. as follow: "*To conduct the meta-analysis, all research articles reporting the AMR of Salmonella spp. in slaughtered cattle, goats and/or sheep were selected. All selected publications were evaluated by all authors to determine their suitability for inclusion in the analysis. The inclusion criteria for the articles involved verifying the resistance to any antimicrobial by Salmonella spp. isolated from any part of the carcass and/or organ of cattle, goats, and/or sheep during slaughter. Publications related to systematic reviews and meta-analyses were not considered for the study. Articles studying the AMR of Salmonella spp. in the slaughterhouse environment, handlers, and/or slaughterhouse wastewater were also excluded. Those*

		scientific papers related about AMR of Salmonella spp. isolated from farms were not considered for meta-analysis."
Information sources	6	**Specify all databases, registers, websites, organisations, reference lists and other sources searched or consulted to identify studies. Specify thedate when each source was last searched or consulted.**<br>Information about scientific sources is described in heading 4.1. as follow: *"Databases such as Google Scholar, Scopus, and PubMed were utilized to retrieve research papers in both English and Spanish."*
Search strategy	7	**Present the full search strategies for all databases, registers and websites, including any filters and limits used.**<br>Scientific strategy is presented in heading 4.1. as follow: *"The article search commenced in September 2022 and concluded in April 2023. Scientific articles should include information on the presence/absence of Salmonella spp. isolated from beef, sheep, and goat carcasses and/or organs, as well as indicate the resistance against various antimicrobials. The search for scientific articles was carried out using boolean terms in EBSCOhost and directly in browser bar of the aforementioned databases. The boolean terms used to identify the relevant scientific articles were "antimicrobial" AND "resistance" AND "slaughterhouse" OR "abattoir" OR "slaughter" AND "bovine" OR "sheep" OR "goat" AND "Salmonella"."*
Selection process	8	**Specify the methods used to decide whether a study met the inclusion criteria of the review, including how many reviewers screened each recordand each report retrieved, whether they worked independently, and if applicable, details of automation tools used in the process.**<br>Innformation of selection process is indicated in heading 4.3 as follow: *"For each reference, the following variables were systematically extracted and entered into a summary table: (1) author, (2) year of publication, (3) specie, (4) country of publication, (5) total samples, (6) total Salmonella spp. positive samples, (7) location of samples, (8) sampling location, (9) name of the antimicrobial substance tested and (10) number of Salmonella spp. samples resistant to each specific antimicrobial. All manuscript included in the meta-analysis are presented in* ***supplementary material****."*
Data collection process	9	**Specify the methods used to collect data from reports, including how many reviewers collected data from each report, whether they worked independently, any processes for obtaining or confirming data from study investigators, and if applicable, details of automation tools used in theprocess.**<br>Information about steps of data collection is presented in heading 4.3.
Data items	10a	**List and define all outcomes for which data were sought. Specify whether all results that were compatible with each outcome domain in each study were sought (e.g. for all measures, time points, analyses), and if not, the methods used to decide which results to collect.**<br>See point 8.
	10b	**List and define all other variables for which data were sought (e.g. participant and intervention characteristics, funding sources). Describe any assumptions made about any missing or unclear information.**<br>See point 8.
Study risk of bias	11	**Specify the methods used to assess risk of bias in the included studies, including details of the tool(s) used, how many reviewers assessed each study and whether they worked independently, and if applicable, details of automation tools used in the process.**

assessment		Information about the assessing of the risk of bias in the included studies was based on a compliance chart of the points 1 to 9 previously described. All studies were carefully screened. If an article not provided clear information of any point, then the scientific study was rejected for inclusion for meta-analysis.
Effect measures	12	**Specify for each outcome the effect measure(s) (e.g. risk ratio, mean difference) used in the synthesis or presentation of results.**<br>The main effect measure is prevalence of *Salmonella* spp. in carcass and/or organs of cattle, sheep and goats in slaughterhouses. The second effect is the prevalence of resistance or susceptibility of Salmonella spp. against specific antimicrobials.
Syntheis methods	13a	**Describe the processes used to decide which studies were eligible for each synthesis (e.g. tabulating the study intervention characteristics andcomparing against the planned groups for each synthesis (item #5)).**<br>PRISMA flowchart for the selection process of eligible studies is presented in table 1.
	13b	**Describe any methods required to prepare the data for presentation or synthesis, such as handling of missing summary statistics, or dataconversions.**<br>data presentation is clearly indicated in the manuscript as follows: *"For the statistical treatment, prevalence regarding antimicrobial resistance of Salmonella spp. extracted from each study was subjected to a Logit transformation to have a more symmetrical distribution that asymptotically approaches the normal distribution. Then, reported results of the occurrence of the antimicrobial resistance of Salmonella spp. and those indicated in the forest plot were transformed back to occurrence values [92]."*
	13c	**Describe any methods used to tabulate or visually display results of individual studies and syntheses.**<br>N/A
	13d	**Describe any methods used to synthesize results and provide a rationale for the choice(s). If meta-analysis was performed, describe the model(s), method(s) to identify the presence and extent of statistical heterogeneity, and software package(s) used.**<br>Model description is indicated in the text as follow: *"Heterogeneity between studies was evaluated using the Q statistic as a test of heterogeneity, and the I^2 statistic [93]. For the pooled estimate, the values were weighted by the inverse of their variances. Random effects models were assumed instead of the fixed-effect model [94]. Random effects models are generally preferred because they are more conservative and allow generalizing conclusions beyond the specific set of studies analyzed [95]. The specific variance (τ^2) was estimated using the restricted maximum likelihood method. All statistical analyses were performed through the metafor R package® [96]."*
	13e	**Describe any methods used to explore possible causes of heterogeneity among study results (e.g. subgroup analysis, meta-regression).**<br>N/A
	13f	**Describe any sensitivity analyses conducted to assess robustness of the synthesized results.**<br>N/A

Reporting bias assessment	14	**Describe any methods used to assess risk of bias due to missing results in a synthesis (arising from reporting biases).**<br>See item 11.
Certainty assessment	15	**Describe any methods used to assess certainty (or confidence) in the body of evidence for an outcome.**<br>See item 11.
**RESULTS**		
Study selection	16a	**Describe the results of the search and selection process, from the number of records identified in the search to the number of studies included inthe review, ideally using a flow diagram.**<br>Results are clearly present in heading 5.
	16b	**Cite studies that might appear to meet the inclusion criteria, but which were excluded, and explain why they were excluded.**<br>Rstudies that did not present all data required were excluded.
Study characteristics	17	**Cite each included study and present its characteristics.**<br>Studies included in the metanalysis was added as supplementary material.
Risk of bias in studies	18	**Present assessments of risk of bias for each included study.**<br>See point 11.
Results of individual studies	19	**For all outcomes, present, for each study: (a) summary statistics for each group (where appropriate) and (b) an effect estimate and its precision(e.g. confidence/credible interval), ideally using structured tables or plots.**<br>Not evaluated.
Results of syntheses	20a	**For each synthesis, briefly summarize the characteristics and risk of bias among contributing studies.**<br>See point 11.
	20b	**Present results of all statistical syntheses conducted. If meta-analysis was done, present for each the summary estimate and its precision (e.g.confidence/credible interval) and measures of statistical heterogeneity. If comparing groups, describe the direction of the effect.**<br>Results of the meta-analysis are clearly present in heading 5.

	20c	**Present results of all investigations of possible causes of heterogeneity among study results.**<br>N/A
	20d	**Present results of all sensitivity analyses conducted to assess the robustness of the synthesized results.**<br>**N/A**
Reporting biases	21	**Present assessments of risk of bias due to missing results (arising from reporting biases) for each synthesis assessed.**<br>See item #11
Certainty of evidence	22	**Present assessments of certainty (or confidence) in the body of evidence for each outcome assessed.**<br>N/A
**DISCUSSION**		
Discussion	23a	**Provide a general interpretation of the results in the context of other evidence.**<br>Appropriate discussion is presented in heading 6.
	23b	**Discuss any limitations of the evidence included in the review.**<br>Limitations of the meta-analysis and systematic review are clearly indicated in the conclusion section as follow: *"However, it is important to highlight that research literature available regarding the antimicrobial resistance of Salmonella spp. isolated in animals to different antibiotics are very limited, mainly for sheep and goats. Furthermore, most of the available scientific articles are outdated and are carried out in developing countries. Therefore, more research is needed to monitor the emergence and/or increase of the resistance of Salmonella spp. to different antibiotics as well as their potential impact on public health within the one health contact."*
	23c	**Discuss any limitations of the review processes used.**<br>See item #23b.
	23d	**Discuss implications of the results for practice, policy, and future research.**<br>Veterinary and public health implications of the results observed in the meta-analysis are clearly discussed and resumed in the conclusion.
**OTHER INFORMATION**		
Registration and protocol	24a	**Provide registration information for the review, including register name and registration number, or state that the review was not registered.** :<br>Information about the absence of registration was added to the text.
	24b	**Indicate where the review protocol can be accessed, or state that a protocol was not prepared.**

		Indication of not prepared protocol" was added to the text.
	24c	**Describe and explain any amendments to information provided at registration or in the protocol.**<br>N/A.
Support	25	**Describe sources of financial or non-financial support for the review, and the role of the funders or sponsors in the review.**<br>Funding is clearly indicated in the manuscript.
Competing interests	26	**Declare any competing interests of review authors.**<br>Any authors declare competing interest. Information is clearly indicated in the manuscript.
Availability of data, code and other materials	27	**Report which of the following are publicly available and where they can be found: template data collection forms; data extracted from includedstudies; data used for all analyses; analytic code; any other materials used in the review.**<br>All information is provided in the manuscript and supplementary material.

Supplementary Material S3. Data about *Salmonella* spp. prevalence from studies analyzed for meta-analysis

Author	TS	PS	Prevalence	Carcass	Lymph	Feces	Liver	Lungs	Rumen	Intestines	Kidney	Spleen	Tonsils	Gallbladd	Specie	YoP	Country	Continent	Protocol for detection of antimicrobial susceptibility	Antimicrobial standard
Abrahan_2022	1001	184	18.38	-	-	184	-	-	-	-	-	-	-	-	Cattle	2022	Autralia	Oceania	Broth microdilution	CLSI[22]
Alemu_2022	150	14	9.33	10	-	-	-	-	-	-	-	-	-	-	Goat	2022	Ethiophia	Africa	Disk difussion	CLSI[24]
Alemu_2021	744	28	3.76	9	6	11	2	-	-	-	-	-	-	-	Cattle	2021	Ethiophia	Africa	Disk diffusion	CLSI[20]
Al-Zubaid_2013	400	11	2.75	-	3	-	3	-	-	-	-	1	-	-	Cattle	2013	Iraq	Asia	Disk diffusion	Bauer et al., 1966
Amera_2017	384	19	4.94	9	-	-	-	-	-	-	-	-	-	-	Cattle	2017	Ethiophia	Africa	Disk diffusion	CLSI[7]
Atlawn_2022	1128	368	32.62	6	-	89	-	-	-	45	-	-	-	-	Sheep	2022	USA	America	Broth microdilution	CLSI[27]<br>NARMS[28]
Bacon_2002	640	53	8.28	53	-	-	-	-	-	-	-	-	-	-	Cattle	2002	USA	America	Disk diffusion	NCCLS*
Barlow_2015	181	106	58.56	-	-	106	-	-	-	-	-	-	-	-	Cattle	2015	Autralia	Oceania	Broth microdilution	CLSI*<br>EUCAST*<br>NARMS*

Bier_2018	270	6	2.22	6	-	-	-	-	-	-	-	-	-	-	Cattle	2018	Brazil	America	Disk diffusion	CLSI[14]
Cetin_2018	1400	8	0.57	2	4	2	0	-	-	-	0	0	-	0	Cattle	2018	Turkey	Europa	Disk diffusion	EUCAST[16,17]
Cetin_2020	1400	10	0.71	0	3	4	0	-	-	-	3	0	-	0	Sheep	2020	Turkey	Europe	Disk diffusion	CLSI[18,19]
Edrington_2009	51	0	0.00	0	0	0	0	0	0	0	0	0	-	-	Sheep	2009	Mexico	USA	Broth microdilution	NCCLS*
Ekli_2019	100	31	31.00	15	-	-	16	-	-	-	-	-	-	-	Cattle	2019	Ghana	Africa	Disk diffusion	CLSI*
Ferede_2015	249	44	17.67	44	-	-	-	-	-	-	-	-	-	-	Goat	2015	Ethiophia	Africa	Disk diffusion	NCCLS*
Gabanan_2022	170	13	7.64	13	-	-	-	-	-	-	-	-	-	-	Cattle	2022	Brazil	America	Disk diffusion	CLSI[23]
Geresu_2021	70	10	14.28	10	-	-	-	-	-	-	-	-	-	-	Cattle	2021	Ethiophia	Africa	Disk diffusion	CLSI[21]
Gragg_2013	171	91	53.21	17	56	18	-	-	-	-	-	-	-	-	Cattle	2013	USA	America	Broth microdilution	CLSI*<br>NAMRS[4,5]
Guteman_2021	350	16	4.57	11	-	5	-	-	-	-	-	-	-	-	Cattle	2021	Ethiophia	Africa	Broth microdilution	EUCAST*
Iglesias_2017	200	16	8.00	16	-	-	-	-	-	-	-	-	-	-	Cattle	2017	Brazil	America	Disk diffusion	CLSI[9]
Kore_K_2017	611	10	1.63	2	4	2	-	-	-	-	-	0	-	-	Cattle	2017	Ethiophia	Africa	Broth microdilution	CLSI[10,11]
Lynne_2009	550	18	3.27	18	-	-	-	-	-	-	-	-	-	-	Cattle	2009	USA	America	Disk diffusion	NCCLS[1,2]
Jajere_2015	54	33	61.11	-	33	-	-	-	-	-	-	-	-	-	Cattle	2015	Nigeria	Africa	Disk diffusion	CLSI[6]
Jajere_2015	66	16	24.24	-	16	-	-	-	-	--	-	-	-	-	Goat	2015	Nigeria	Africa	Disk diffusion	CLSI[6]
Jajere_2015	12	4	33.33	-	4	-	-	-	-	-	-	-	-	-	Sheep	2015	Nigeria	Africa	Disk diffusion	CLSI[6]
Jitjak_2016	120	1	0.83	-	-	-	-	-	-	-	-	-	-	-	Cattle	2016	Thailand	Asia	Disk diffusion	CLSI[7]
Kebede_2016	140	8	5.71	4	-	-	2	2	-	-	-	-	-	-	Cattle	2016	Ethiophia	Africa	Disk diffusion	CLSI[6]
Ketema_2018	726	27	3.71	4	-	23	-	-	-	-	-	-	-	-	Cattle	2018	Ethiophia	Africa	Disk diffusion	CLSI[15]
Madoroba_2016	629	92	14.62	167	-	400	-	-	-	62	-	-	-	-	Cattle	2016	South Africa	Africa	Disk diffusion	CLSI*
Mathole_2016	474	2	0.42	2	-	-	-	-	-	-	-	-	-	-	Goat	2016	South Africa	Africa	Disk difussion	CLSI[7]
McEvoy_2003	750	29	3.86	19	-	5	-	-	5	-	-	-	-	-	Cattle	2003	Ireland	Europe	Disk difussion	Bauer et al., 1966
Morar_2015	108	26	24.07	19	-	-	-	-	-	-	-	-	-	-	Cattle	2015	Romania	Europe	Broth microdilution	*
Mouichn_2018	826	84	10.16	441	-	385	-	-	-	-	-	-	-	-	Cattle	2018	Algeira	Africa	Disk diffusion	CLSI[13]
Mustafa_2018	300	17	5.66	-	10	7	-	-	-	-	-	-	-	-	Cattle	2018	Ethiophia	Africa	Disk diffusion	CLSI[15]
Obaidat_2020	1036	287	27.70	-	-	144	-	-	-	143	-	-	-	-	Cattle	2020	Jordan	Asia	Disk diffusion	CLSI*

Oueslati_2016	300	17	5.66	17	-	-	-	-	-	-	-	-	-	-	Cattle	2016	Tunisia	Africa	Disk diffusion	CLSI[8]
Shaibu_2021	315	11	3.49	4	-	1	-	-	-	-	-	-	-	-	Cattle	2021	Nigeria	Africa	Disk diffusion	Magiorakos et al., 2011
Stevens_2006	236	99	41.95	99	-	-	-	-	-	-	-	-	-	-	Cattle	2006	Senegal	Africa	Broth microdilution	n/a
Sibhatn_2009	800	87	10.88	45	8	6	0	-	19	-	-	-	-	-	Cattle	2009	Ethiophia	Africa	Disk diffusion	NCCLS*
Shilangale_2015	9508	45	0.47	45	-	-	-	-	-	-	-	-	-	-	Cattle	2015	Namibia	Africa	Disk diffusion	CLSI[1]
Takelen_2018	440	42	9.54	22	-	11	-	-	-	-	-	-	-	-	Cattle	2018	Ethiophia	Africa	Disk diffusion	CLSI[15]
Varela_Guerrero_2013	327	27	8.25	27	-	-	-	-	-	-	-	-	-	-	Cattle	2013	Mexico	America	Broth microdilution	NCCLS[3]
Wabeto_2017	448	56	12.50	56	-	-	-	-	-	-	-	-	-	-	Cattle	2017	Ethiophia	Africa	Disk diffusion	EUCAST[12]
Wang_2020	138	41	29.71	41	-	-	-	-	-	-	-	-	-	-	Cattle	2020	China	Asia	Broth microdilution	CLSI[18,19]
Webb_2017	5450	376	6.89	X	376	-	-	-	-	-	-	-	-	-	Cattle	2017	USA	America	Broth microdilution	CLSI*
Wieczorek_2013	812	9	1.10	9	-	-	-	-	-	-	-	-	-	-	Cattle	2013	Poland	Europe	Broth microdilution	Sensititre® custom susceptibility plates,
Wottlin_2022	212	21	9.90	-	17	4	-	-	-	-	-	-	-	-	Cattle	2022	USA	America	Broth microdilution	CLSI[25] NARMS[26]
Zare_2014	80	14	17.50	-	-	5	-	-	-	-	-	-	-	2	Cattle	2014	Azarbaijan	Asia	Disk diffusion	CLSI[3]
Zare_2014	80	4	5.00	-	-	4	-	-	-	-	-	-	-	4	Goat	2014	Azarbaijan	Asia	Disk diffusion	CLSI[3]
Zare_2014	80	3	3.75	-	-	2	-	-	-	-	-	-	-	1	Sheep	2014	Azarbaijan	Asia	Disk diffusion	CLSI[3]

TS: total samples; PS: *Salmonella* spp. positive samples.

*: not reference is provided in the manuscript. CLSI: Clinical and laboratory standards institute, NCCLS: National committee for clinical laboratory standards; NARMS: National antimicrobial resistance monotoring system.

[1]NCCLS **(2002)**. National Committee for Clinical Laboratory Standards. Performance standards for antimicrobial susceptibility testing. Twelfth informational supplement (M100-S12), (M100-S12) ed., Wayne, PA.

[2]NCCLS **(2002).** National Committee for Clinical Laboratory Standards. Performance standards for antimicrobial disk and diffusion susceptibility testing for bacteria isolated from animals; approved standard-second edition (M31-A2). Wayne, PA.

[3]NCLS **(2009).** National Committee for Clinical Laboratory Standards. Performance standards for antimicrobial susceptibility testing; Nineteenth Informational Supplement. 10 ed. vol. 29, no. 3. Approved standard M100-S19. National Committee for Clinical Laboratory Standards, Wayne, P.A.

[4]CDC **(2012)**. National Antimicrobial Monitoring System for enteric bacteria (NARMS): human isolates final report, 2010. US Department of Health and Human Services, Atlanta, GA.8.

[5]CDC **(2012)**. National Antimicrobial Resistance Monitoring System for enteric bacteria (NARMS): manual of laboratory methods. US Department of Health and Human Services, Atlanta, Georgia.

[6]CLSI **(2010)** Clinical and Laboratory Standards Institute. Performance standards for antimicrobial susceptibility testing; twenty-second informational supplement. Wayne, PA: Clinical and Laboratory Standards Institute; 2010. [Online] Available from: http://antimicrobianos.com.ar/ATB/wpcontent/uploads/2012/11/M100S22E.pdf

[7]CLSI. **(2014)**. Clinical and Laboratory Standards Institute (CLSI). (2014). Performance standards for antimicrobial susceptibility testing; twenty-fourth informational supplement. Wayne, PA: Clinical and Laboratory Standards Institute. CLSI document M100-S24.

[8]CLSI **(2008)**. Clinical and Laboratory of Standards Institute Performance standards for antimicrobial susceptibility testing (2008) Documents M100-S18, Wayne, PA19087 USA.

[9]CLSI **(2015)**. Clinical and Laboratory Standards Institute (CLSI) (2015). M100eS25 Performance Standards for Antimicrobial.

[10]CLSI **(2013)**. Performance Standards for Antimicrobial Disk and Dilution Susceptibility Tests for Bacteria Isolated from Animals, 2nd Informational Supplement VET01-S2.

[11]CLSI **(2015)**. Performance standards for antimicrobial susceptibility testing: 25th informational supplement. *CLSI document M100-S25. Clinical and Laboratory Standards Institute*.

[12]EUCAST, European Committee on Antimicrobial Susceptibility Testing.

[13]CLSI **(2008)**. Clinical Laboratory Standard Institute, 2008). Performance Standards for Antimicrobial Disk and Dilution Susceptibility Tests for Bacteria Isolated from Animals; Approved standard –Third Edition M31-A3 .Vol.28 N° 8. Replaces M31-A2. Vol.22 N°06. February 2008.

[14]CLSI **(2013)**. Performance standards for antimicrobial susceptibility testing; twenty-third informational SupplementM100-S23, vol. 33, 2013.

[15]CLSI **(2012)**. Clinical and Laboratory Standards Institute (CLSI). Performance for antimicrobial disk susceptibility tests; approved standard Wayne, USA , Volume 32(1). 11th edition CLSI document M02-A11. 2012. p. 1 –76.

[16]EUCAST **(2015a)**. Breakpoint tables for Interpretation of MICs and zone diameters. Version 5.0. Basel, Switzerland.

[17]EUCAST **(2015b)**. Antimicrobial susceptibility testing EUCAST disk diffusion method. Version 5.0. Basel, Switzerland.

[18]CLSI **(2008)**. Performance Standards for Antimicrobial Disk and Dilution Susceptibility Tests for Bacteria Isolated from Animals: Informational Supplement, M31-A3; Clinical and Laboratory Standards Institute: Wayne, PA, USA.

[19]CLSI **(2012).** Performance Standards for Antimicrobial Disk and Dilution Susceptibility Tests for Bacteria Isolated from Animals: Twenty-Second Informational Supplement M100-S22; Clinical and Laboratory Standards Institute: Wayne, PA, USA.

[20]NCCLS **(1997)**. Performance standards for antimicrobial disc and dilution susceptibility tests for bacteria isolated from animals and human. Approved standard, NCCLS Document M31-A, NCCLS, Villanova, PA.

[21]CLSI **(2015)**. Clinical and Laboratory Standards Institute. Performance standards for antimicrobial susceptibility testing; 24th informational supplement. M100– S24. Wayne, PA: Clinical and Laboratory Standards Institute.

Magiorakos, P., Srinivasan, A., Carey, R.B., Carmeli, Y., Falagas, M.E., Giske, C.G., Monnet, D.L. Multidrug-resistant, extensively drug-resistant and pandrug-resistant bacteria: An international expert proposal for interim standard definitions for acquired resistance. *Clin. Microbiol. Infect*. **2011**, 18, 268-281. http://doi.org/10.1111/j.1469-0691.2011.03570.x

[22]CLSI **(2015)**. Clinical and Laboratory Standards Institute. Methods for Dilution Antimicrobial Susceptibility Tests for Bacteria That Grow Aerobicaly; Approved Standard (5TH Ed. Wayne, PA, document M07-A10

[23]CLSI **(2020)**. Clinical and Laboratory Standards Institute. Performance standards for antimicrobial susceptibility testing, 30th ed

[24]CLSI **(2018)**. Performance Standard for Antimicrobial Susceptibility Testing. 28th ed. Clinical and Laboratory Standards Institute (CLSI) supplement M100; 2018:1–60.

[25]CLSI **(2017)**. Performance Standards for Antimicrobial Susceptibility Testing, 27th Edn. CLSIsupplement M100. Wayne, PA: Clinical and Laboratory Standards Institute.

[26]NARMS **(2019)**. Antibiotics Tested by NARMS. National Antimicrobial Resistance Monitoring System. Available online at: https://www.cdc.gov/narms/antibiotics-tested.html

[27]CLSI **(2017)**. Standards for antimicrobial susceptibility testing. Performance Standards for Antimicrobial Susceptibility Testing (27th ed.), CLSI supplement M100, Clinical and Laboratory Standards Institute, Wayne, PA.

[28]NARMS **(2020)**. Antibiotics Tested by NARMS. https://doi:10.1089=fpd.2010.0615. https://www.cdc.gov/narms/antibiotics-tested.html

Bauer, A.W., Kirby, W.M. M., Sherris, J.C., Turck, M. Antibiotic susceptibility testing by a standardized single disk method. *Am. J. Clin. Pathol*. **1955**, 36, 493-496.

a)

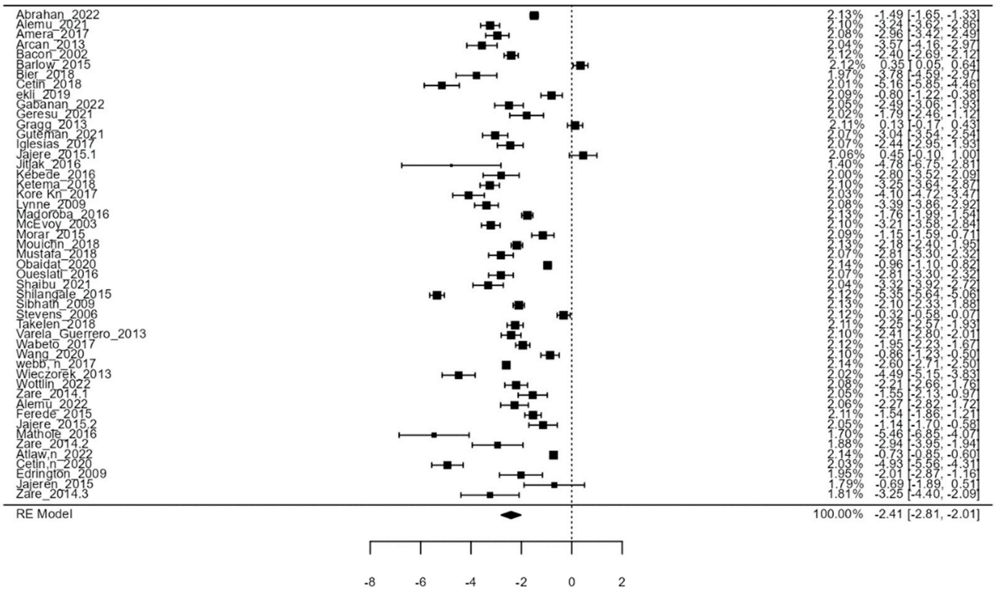

b)

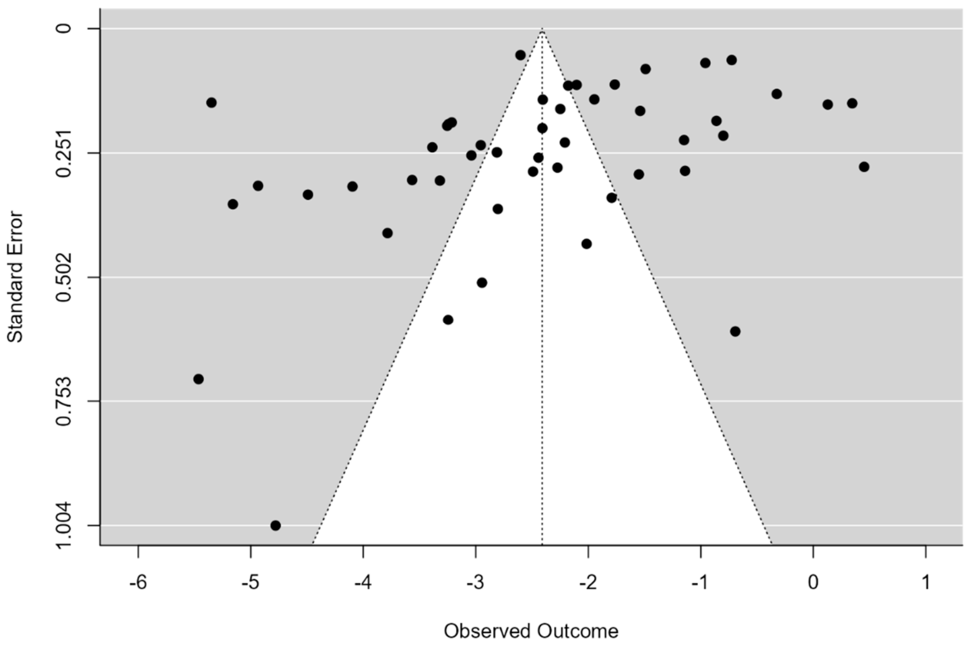

Supplementary Material S4. Forest plot *a)* and funnel plot *b)* of *Salmonella* spp. prevalence in slaughtered large and small ruminants

a)

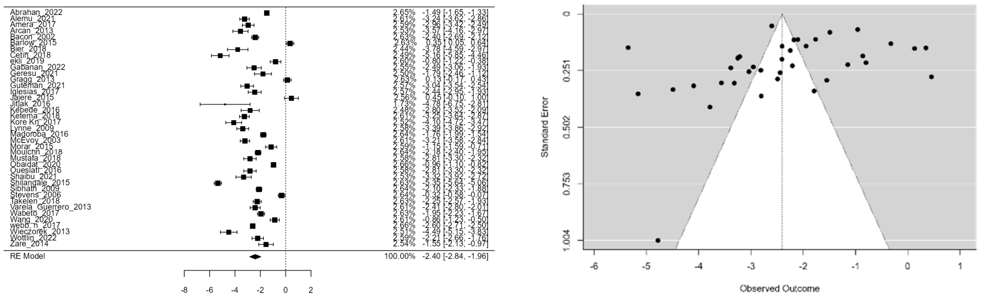

b)

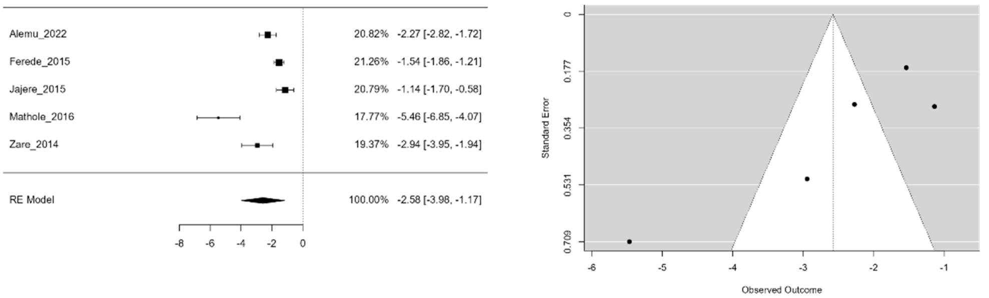

c)

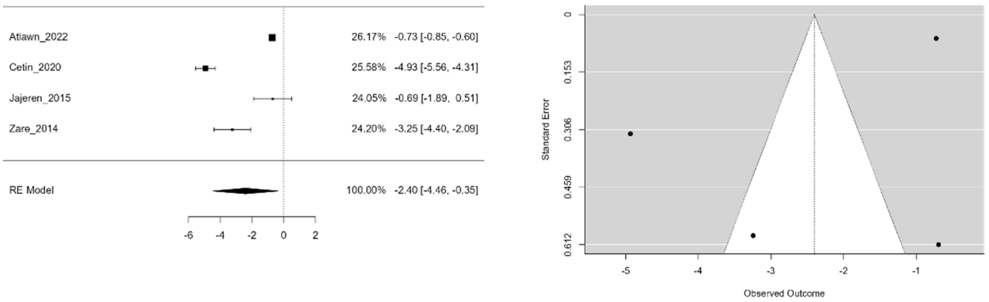

Supplementary Material S5. Forest and funnel plots of prevalence of *Salmonella* spp. by specie (a: cattle, b: goat, c: sheep)

a)

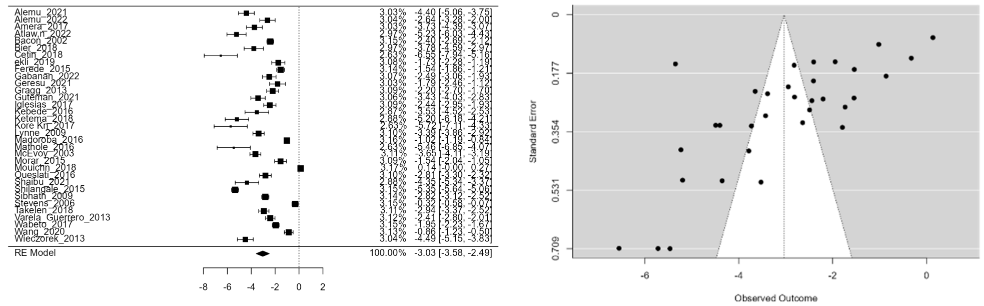

b)

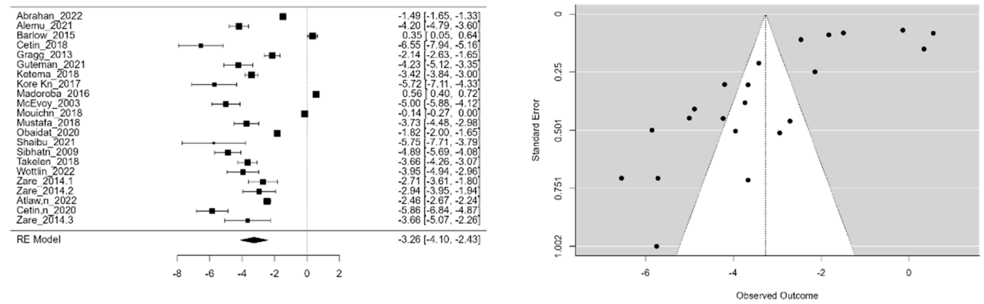

c)

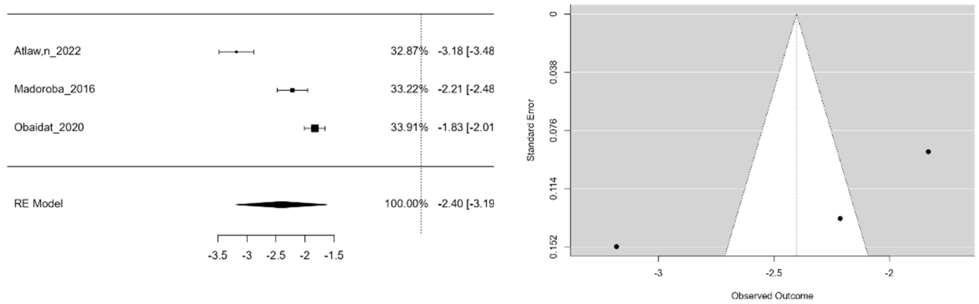

d)

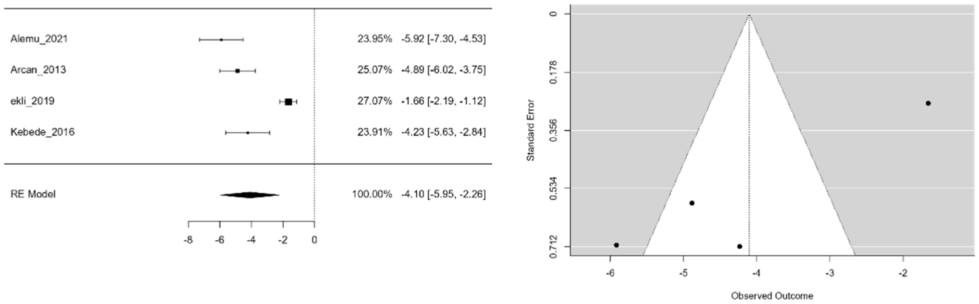

e)

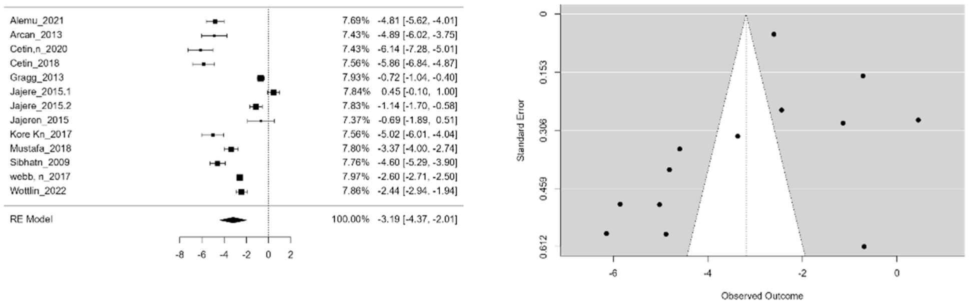

Supplementary Material S6. Forest and funnel plots of prevalence of *Salmonella* spp. by sample location. a): carcass, b): feces, c): intestinal mucosa, d):liver, e): lymph nodes

Amikacin

a)

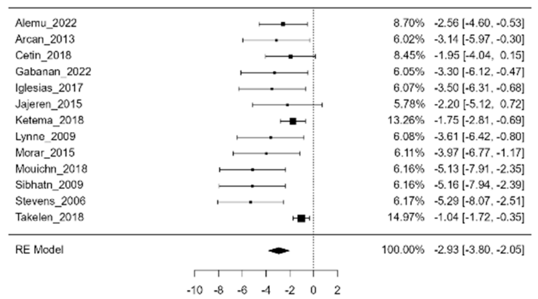

b)

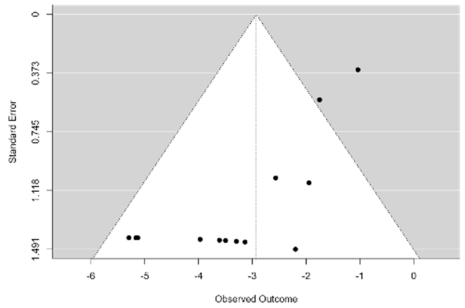

Gentamicin

a)

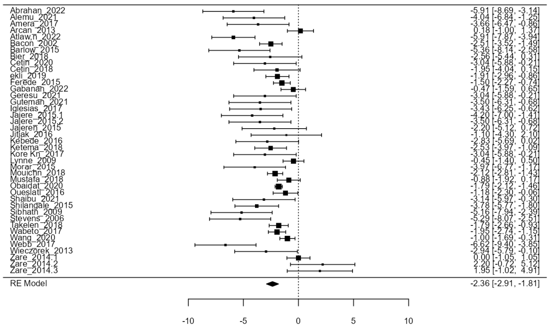

b)

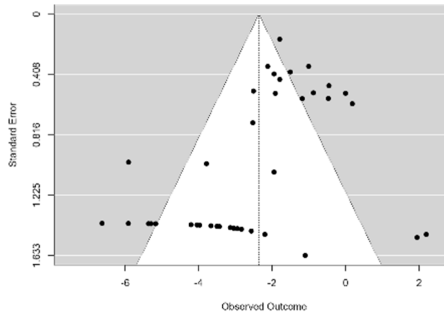

Kanamycin

a)

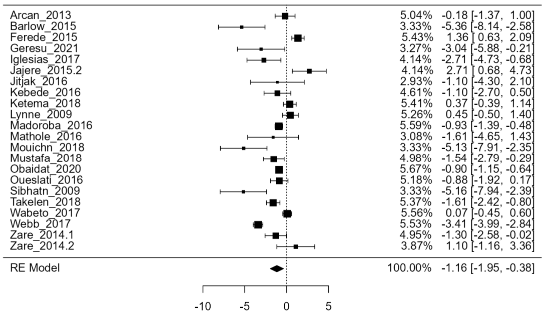

b)

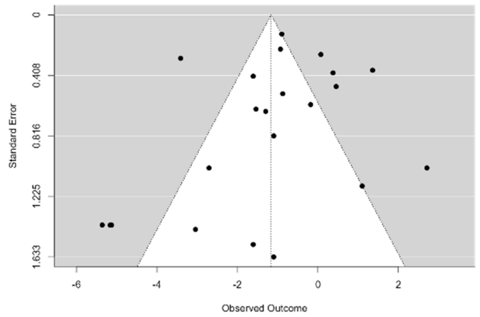

Neomycin

a)

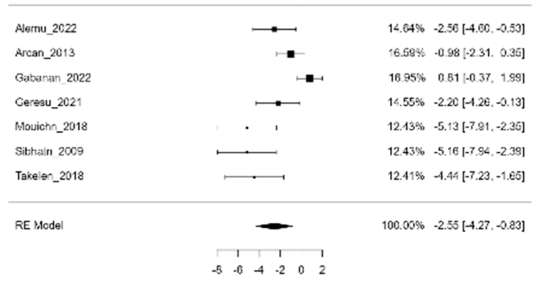

b)

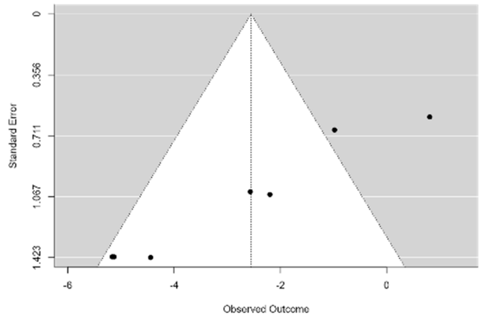

Tobramycin

a)

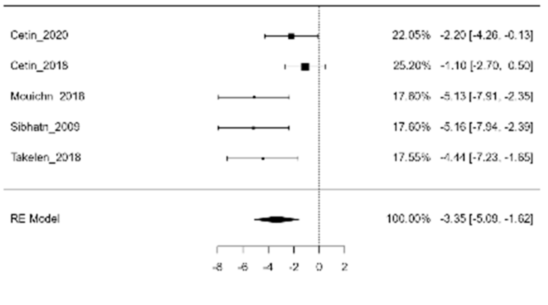

b)

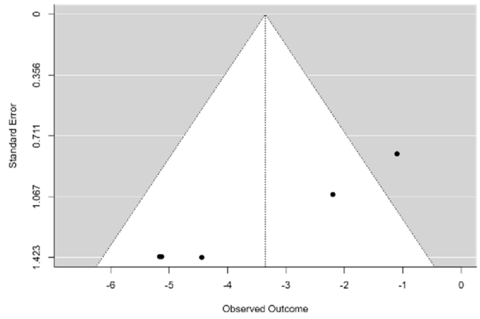

Supplementary Material S7. Forest plots *a)* and funnel plots *b)* of antimicrobial resistance of *Salmonella* spp. by antimicrobial.

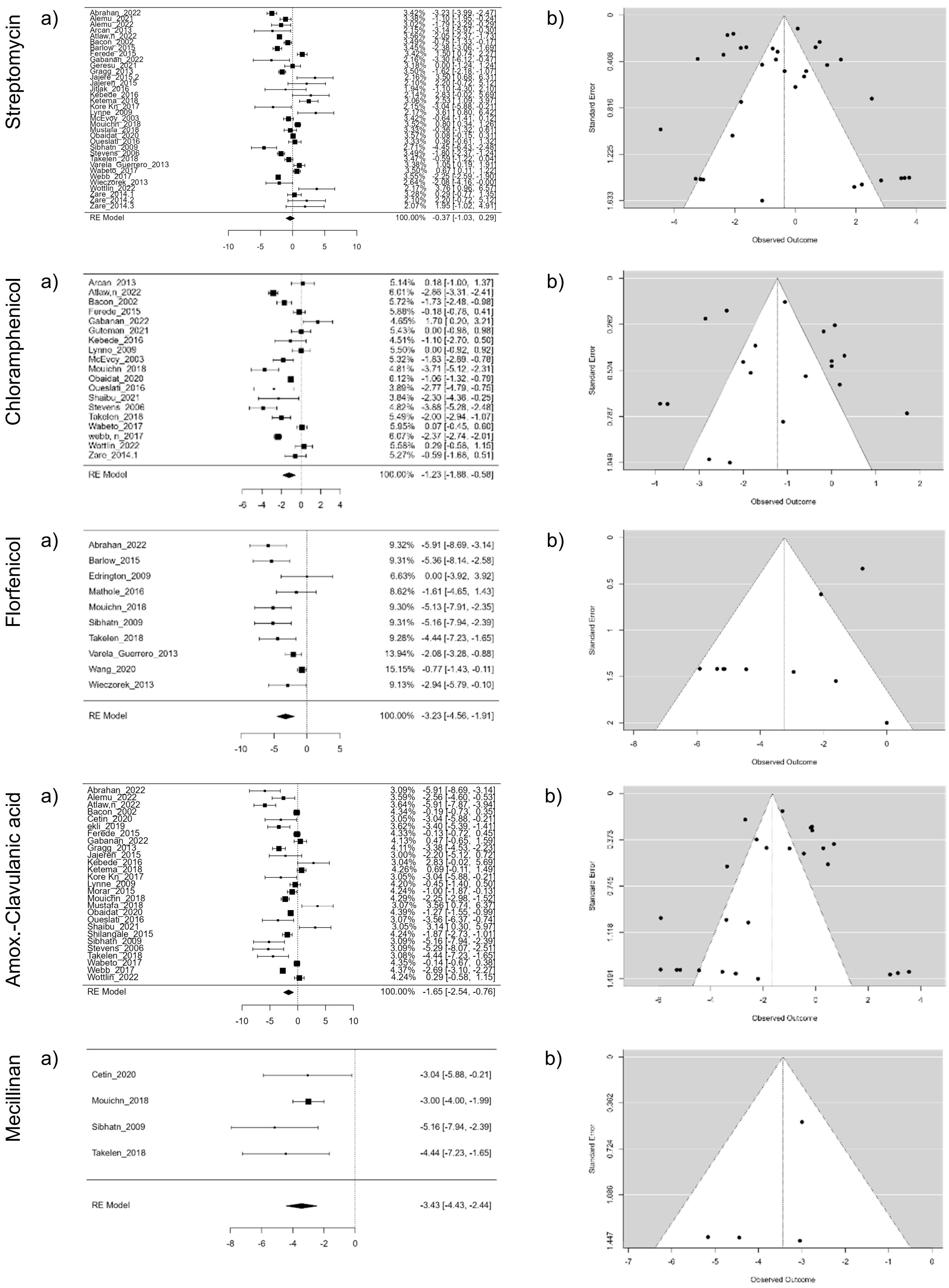

Supplementary material 7. *(Continued)*. Forest plots *a)* and funnel plots *b)* of antimicrobial resistance of *Salmonella* spp. by antimicrobial.

Ticarcillin

a)

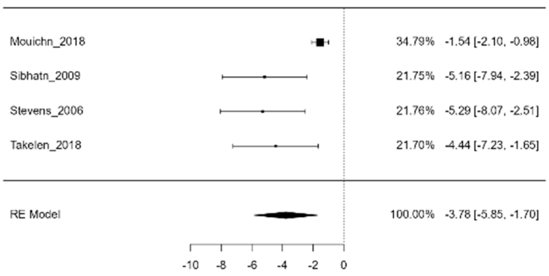

b)

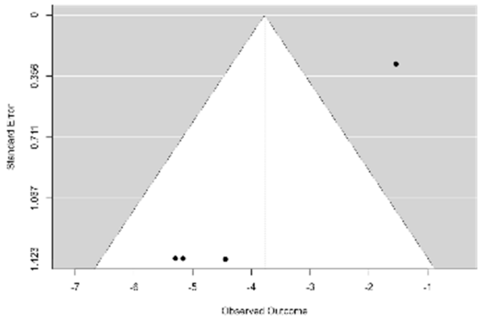

Imipenen

a)

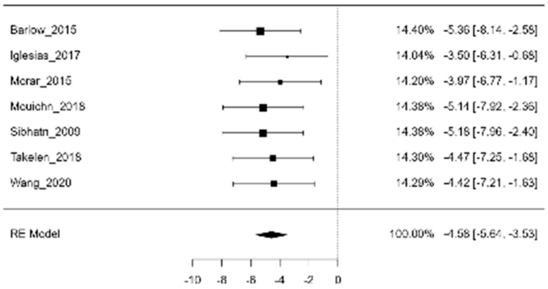

b)

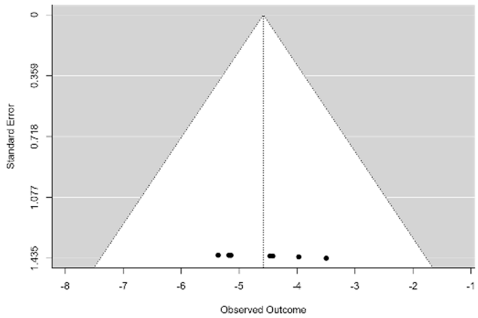

Cephalotin

a)

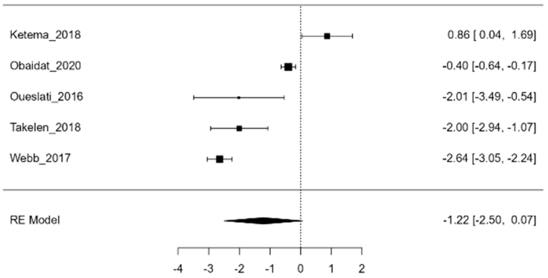

b)

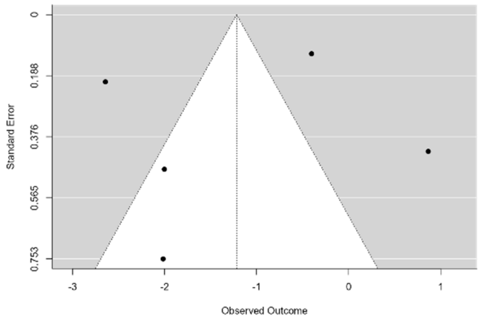

Cafazolie

a)

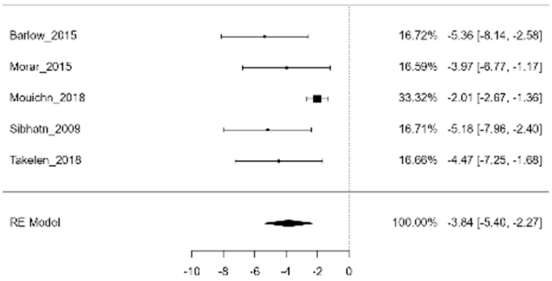

b)

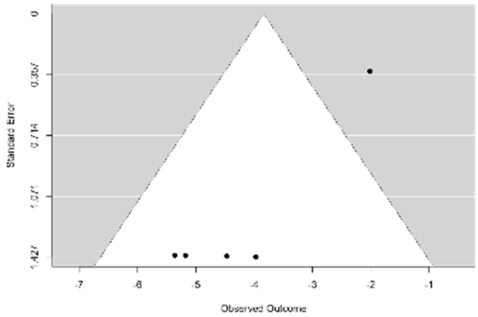

Cefexime

a)

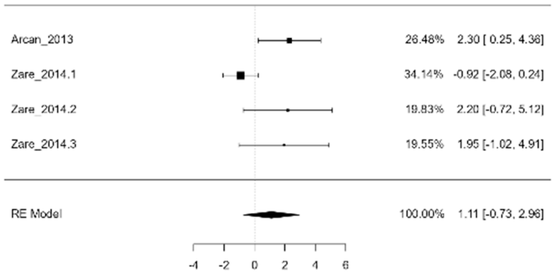

b)

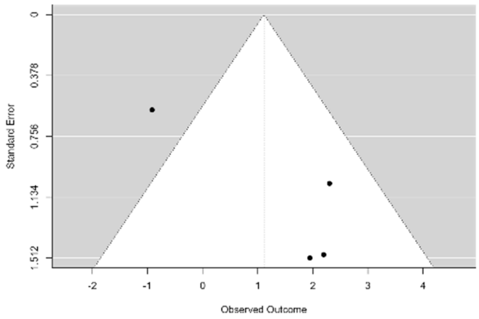

Supplementary Material S7. *(Continued)*. Forest plots *a)* and funnel plots *b)* of antimicrobial resistance of *Salmonella* spp. by antimicrobial.

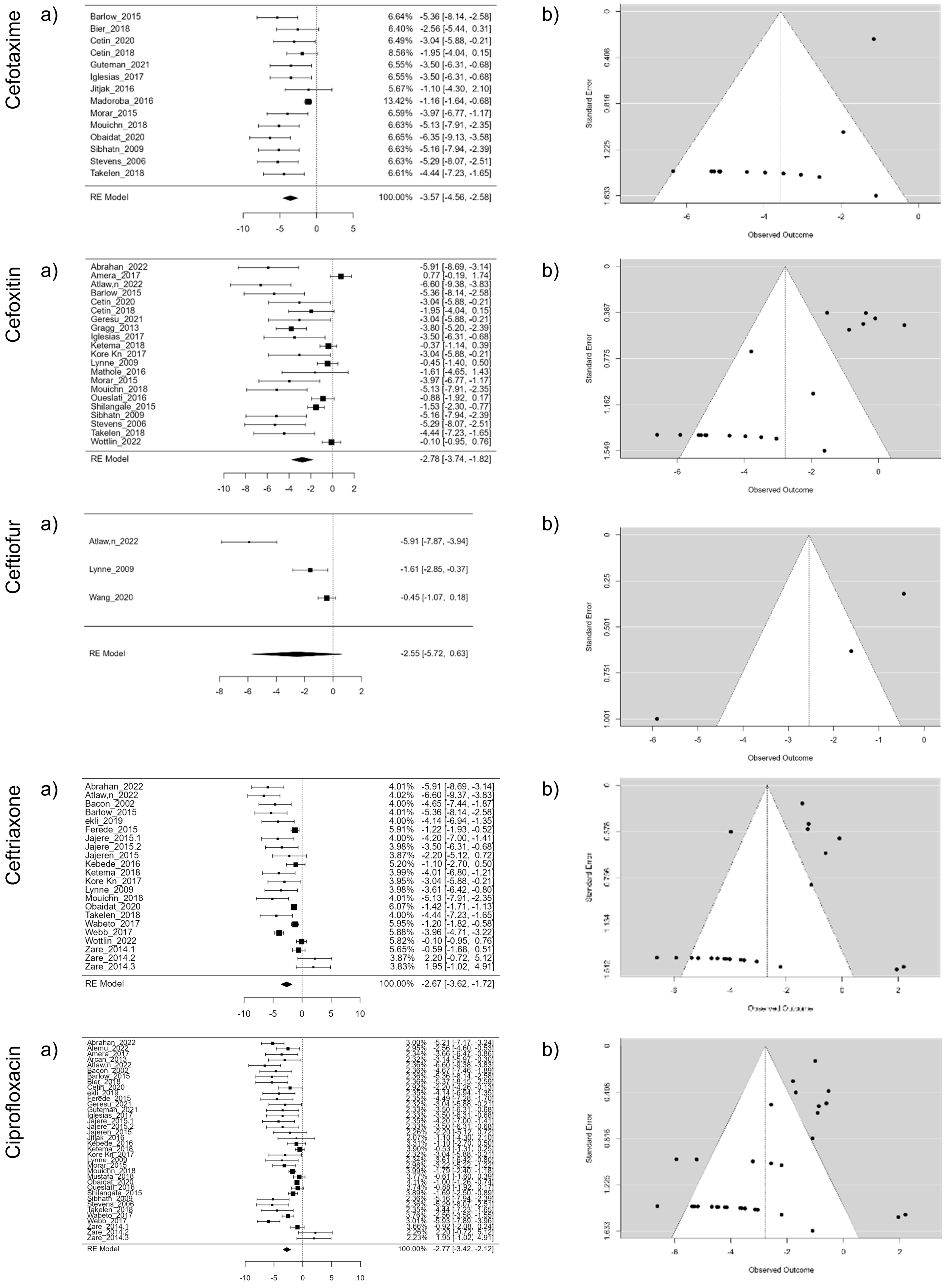

Supplementary material 7. *(Continued)*. Forest plots *a)* and funnel plots *b)* of antimicrobial resistance of *Salmonella* spp. by antimicrobial.

Enrofloxacin

a)

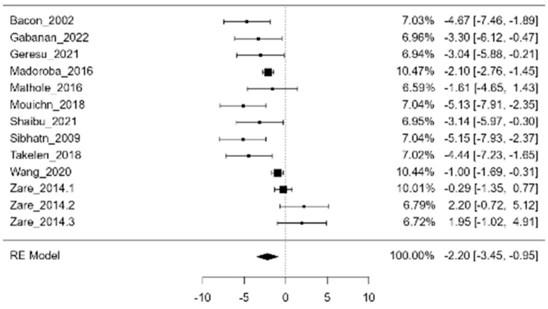

b)

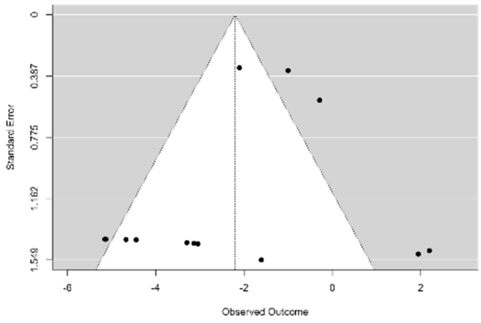

Nalidixic acid

a)

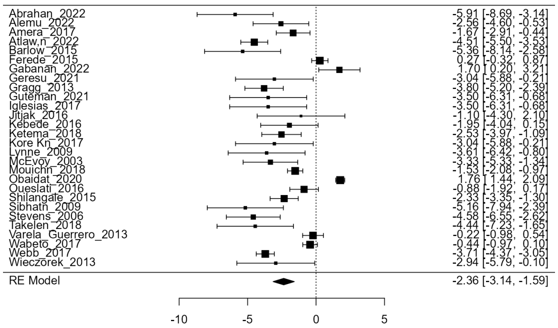

b)

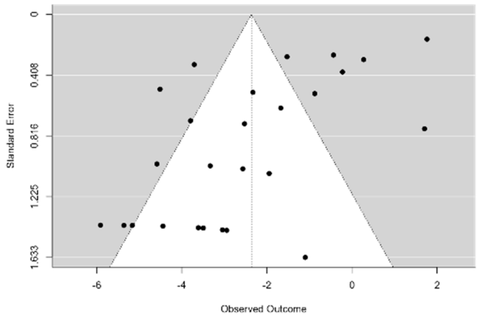

Norfloxacin

a)

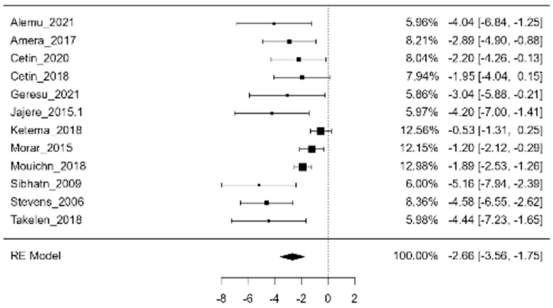

b)

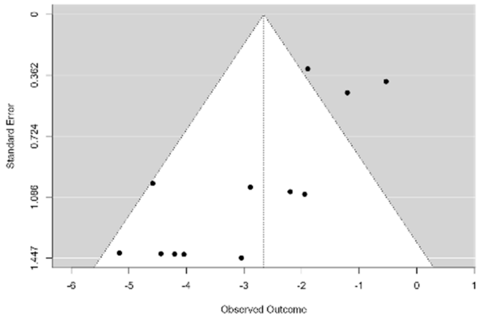

Clindamycin

a)

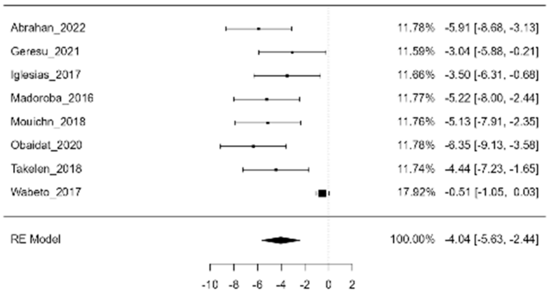

b)

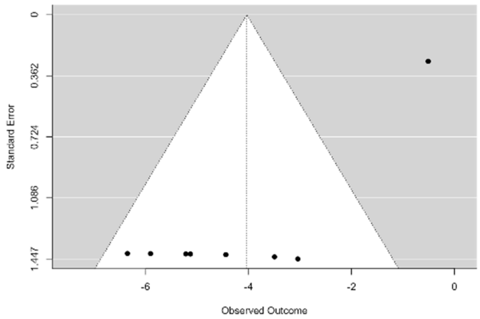

Azithromycin

a)

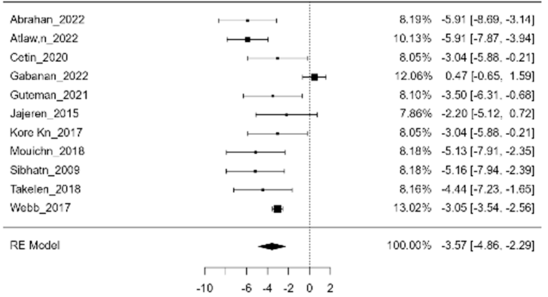

b)

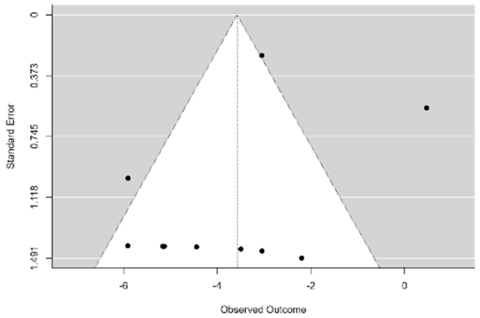

Supplementary Material S7. *(Continued)*. Forest plots *a)* and funnel plots *b)* of antimicrobial resistance of *Salmonella* spp. by antimicrobial.

Erythromycin

a)

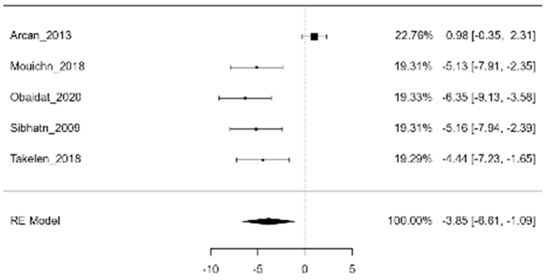

b)

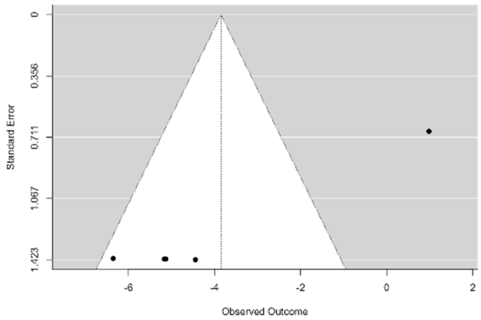

Colistin

a)

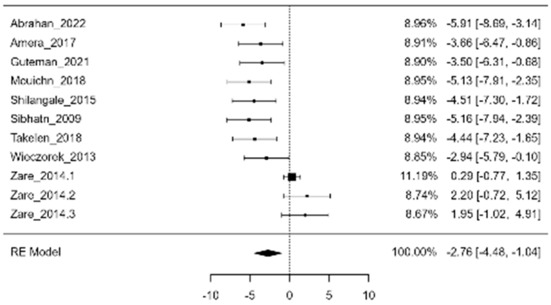

b)

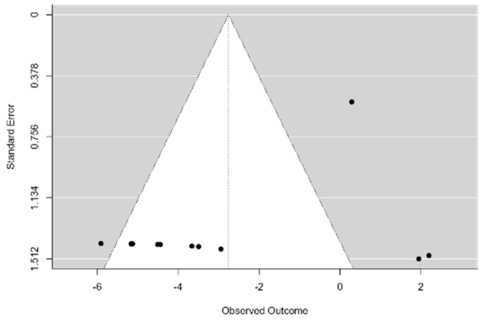

Amoxicilin

a)

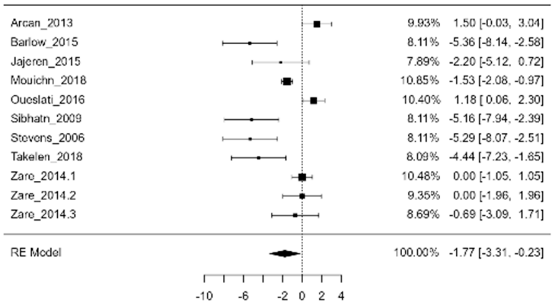

b)

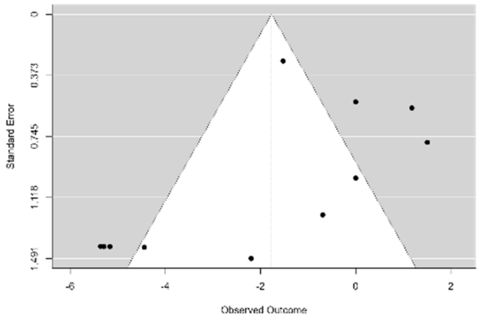

Ampicillin

a)

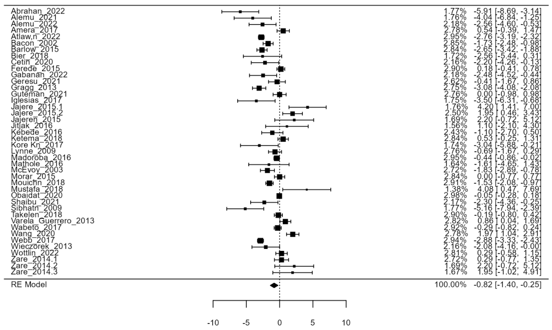

b)

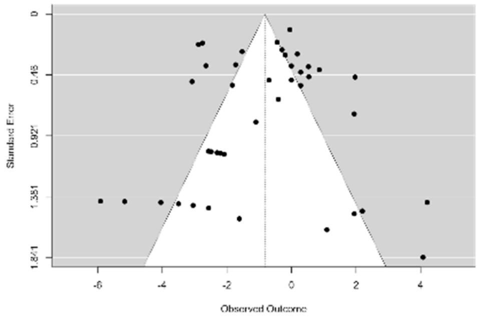

Penicillin

a)

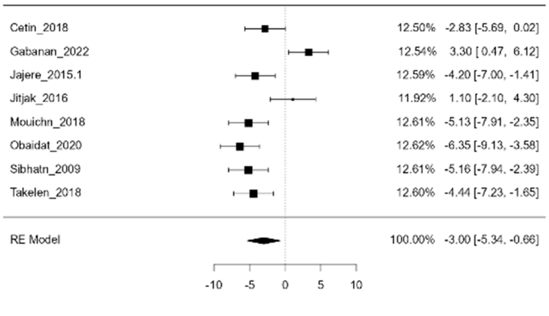

b)

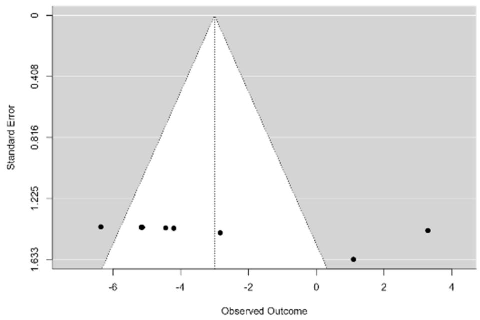

Supplementary Material S7. *(Continued)*. Forest plots *a)* and funnel plots *b)* of antimicrobial resistance of *Salmonella* spp. by antimicrobial.

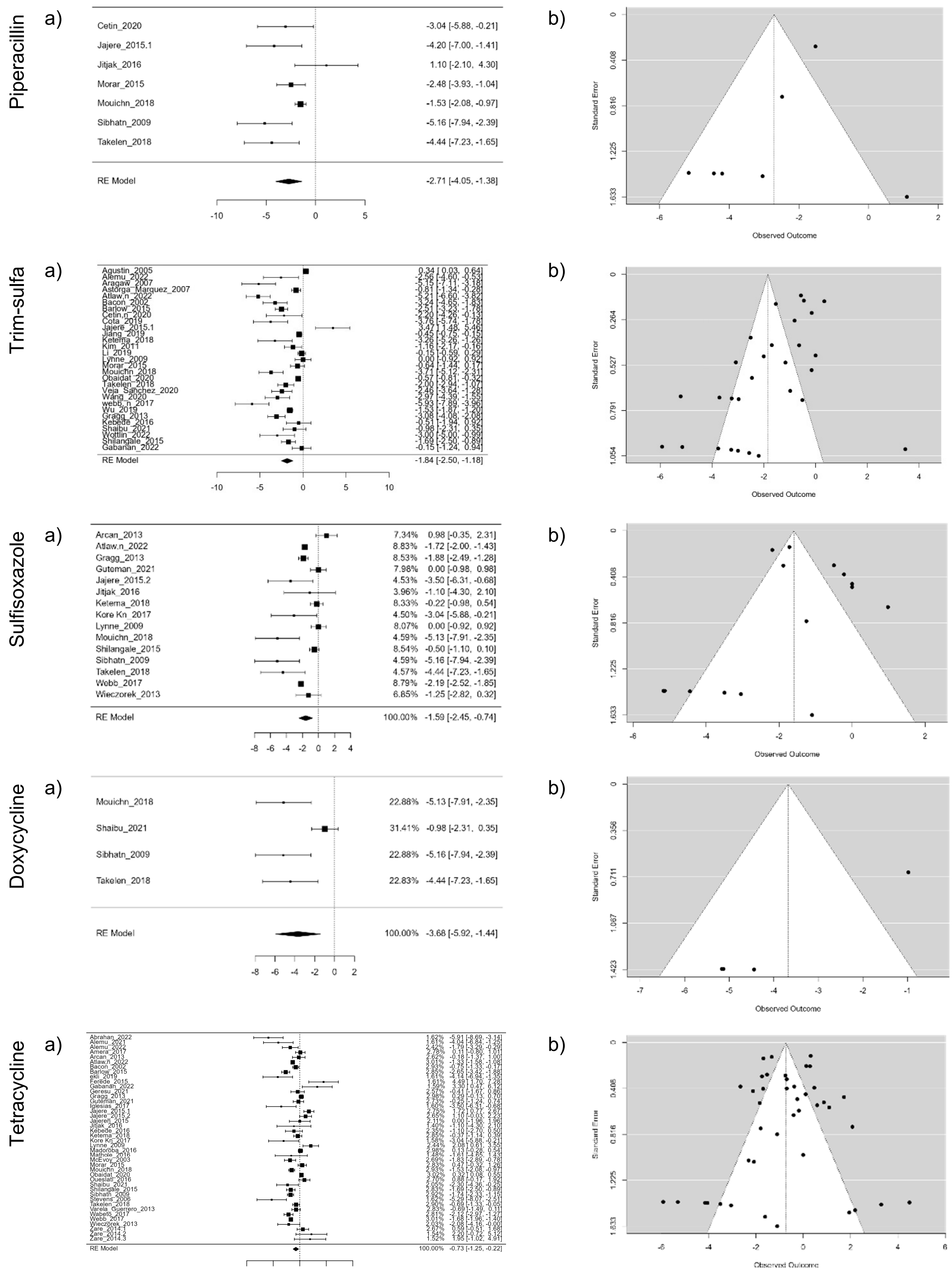

Supplementary Material S7. *(Continued)*. Forest plots *a)* and funnel plots *b)* of antimicrobial resistance of *Salmonella* spp. by antimicrobial.

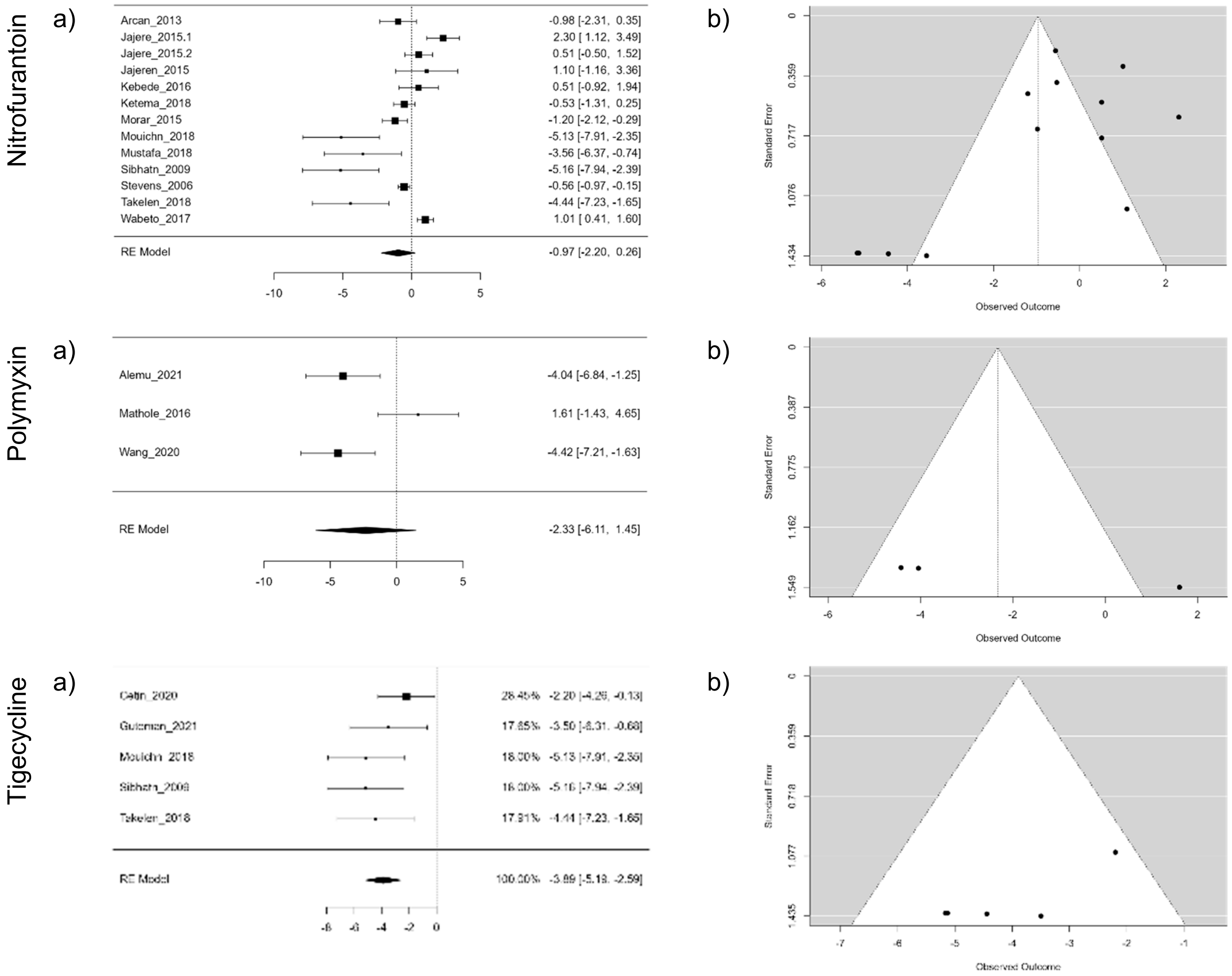

Supplementary Mateial S7. *(Continued)*. Forest plots *a)* and funnel plots *b)* of antimicrobial resistance of *Salmonella* spp. by antimicrobial.

Supplementary Material S8. Prevalence of antimicrobial resistance os *Salmonella* spp. by antimicrobial substance

Author	PS (n)	Amikacin		Gentamicin		Kanamicin		Amoxicylin		Amox-Clav.		Ampicylin		Apramycin		Azitromycin		Aztreonan	
		n	p	n	p	n	p	n	p	n	p	n	p	n	p	n	p	n	p
Abrahan_2022	184	-	-	0	0.00	-	-	-	-	0	0.00	0	0.00	-	-	0	0.00	-	-
Alemu_2021	28	-	-	0	0.00	-	-	-	-	-	-	0	0.00	-	-	-	-	-	-
Alemu_2022	14	1	7.14	-	-	-	-	-	-	1	7.14	1	7.14	-	-	-	-	-	-
Al-Zubaid_2013	11	0	0.00	6	54.55	5	45.45	9	81.82	-	-	-	-	-	-	-	-	-	-
Amera_2017	19	-	-	0	0.00	-	-	-	-	-	-	12	63.16	-	-	-	-	-	-
Atlaw_2022	368	-	-	1	0.27	-	-	-	-	1	0.27	22	5.98	-	-	1	0.27	-	-
Bacon_2002	53	-	-	4	7.55	-	-	-	-	24	45.28	8	15.09	-	-	-	-	-	-
Barlow_2015	106	-	-	0	0.00	0	0.00	0	0.00	-	-	7	6.60	-	-	-	-	-	-
Bier_2018	6	-	-	0	0.00	-	-	-	-	-	-	0	0.00	-	-	-	-	-	-
Cetin_2020	10	0	0.00	0	0.00	-	-	-	-	0	0.00	1	10.00	-	-	0	0.00	-	-
Cetin_2018	8	1	12.50	1	12.50	-	-	-	-	-	-	-	-	-	-	-	-	-	0.00
Edrington_2009	0	-	-	-	-	-	-	-	-	-	-	-	-	-	-	-	-	-	-
Ekli_2019	31	-	-	4	12.90	-	-	-	-	1	3.23	-	-	-	-	-	-	-	-
Ferede_2015	44	-	-	8	18.18	35	79.55	-	-	20	45.45	24	54.55	-	-	-	-	-	-
Gabanan_2022	13	0	0.00	5	38.46	-	-	-	-	8	61.54	1	7.69	-	-	8	61.54	-	-
Geresu_2021	10	-	-	0	0.00	0	0.00	-	-	-	-	4	40.00	-	-	-	-	-	-
Gragg_2013	91	-	-	-	-	-	-	-	-	3	3.30	4	4.40	-	-	-	-	-	-
Guteman_2021	16	-	-	0	0.00	-	-	-	-	-	-	8	50.00	-	-	0	0.00	-	-
Iglesias_2017	16	0	0.00	1	6.25	1	6.25	-	-	-	-	0	0.00	-	-	-	-	-	-
Jajere_2015	33	-	-	0	0.00	33	100.00	-	-	-	-	33	100.00	-	-	-	-	0	0.00
Jajere_2015	16	-	-	0	0.00	15	93.75	-	-	-	-	14	87.50	-	-	-	-	-	-
Jajeren_2015	4	0	0.00	0	0.00	4	100.00	0	0.00	0	0.00	4	100.00	-	-	0	0.00	-	-
Jitjak_2016	1	-	-	0	0.00	0	0.00	-	-	-	-	1	100.00	-	-	-	-	-	-
Kebede_2016	8	-	-	0	0.00	2	25.00	-	-	8	100.00	2	25.00	-	-	-	-	-	-
Ketema_2018	27	4	14.81	2	7.41	16	59.26	-	-	18	66.67	17	62.96	-	-	-	-	-	-
Kore_2017	10	-	-	0	0.00	-	-	-	-	0	0.00	0	0.00	-	-	0	0.00	-	-
Lynne_2009	18	0	0.00	7	38.89	11	61.11	-	-	7	38.89	6	33.33	-	-	-	-	-	-
Madoroba_2016	92	-	-	-	-	26	28.26	-	-	-	-	36	39.13	-	-	-	-	-	-
Mathole_2016	2	-	-	-	-	0	0.00	-	-	-	-	0	0.00	-	-	-	-	-	-
McEvoy_2003	29	-	-	-	-	-	-	-	-	-	-	4	13.79	-	-	-	-	-	-
Morar_2015	26	0	0.00	0	0.00	-	-	-	-	7	26.92	13	50.00	-	-	-	-	-	-

Mouichn_2018	84	0	0.00	9	10.71	0	0.00	15	17.86	8	9.52	15	17.86	-	-	0	0.00	0	0.00
Mustafa_2018	17	-	-	5	29.41	3	17.65	-	-	17	100.00	17	100.00	-	-	-	-	-	-
Obaidat_2020	287	-	-	41	14.29	83	28.92	-	-	63	21.95	140	48.78	-	-	-	-	0	0.00
Oueslati_2016	17	-	-	4	23.53	5	29.41	13	76.47	0	0.00	-	-	-	-	-	-	-	-
Shaibu_2021	11	-	-	0	0.00	-	-	-	-	11	100.00	1	9.09	-	-	-	-	-	-
Shilangale_2015	45	-	-	1	2.22	-	-	-	-	6	13.33	-	-	-	-	-	-	-	-
Sibhatn_2009	87	0	0.00	0	0.00	0	0.00	0	0.00	0	0.00	0	0.00	-	-	0	0.00	0	0.00
Stevens_2006	99	0	0.00	0	0.00	-	-	0	0.00	0	0.00	-	-	-	-	-	-	-	-
Takelen_2018	42	11	26.19	6	14.29	7	16.67	0	0.00	0	0.00	19	45.24	-	-	0	0.00	0	0.00
Varela_Guerrero_2013	27	-	-	-	-	-	-	-	-	-	-	19	70.37	-	-	-	-	-	-
Wabeto_2017	56	-	-	7	12.50	29	51.79	-	-	26	46.43	24	42.86	-	-	-	-	-	-
Wang_2020	41	-	-	11	26.83	-	-	-	-	-	-	36	87.80	-	-	-	-	-	-
Webb_2017	376	-	-	0	0.00	12	3.19	-	-	24	6.38	20	5.32	-	-	17	4.52	-	-
Wieczorek_2013	9	-	-	0	0.00	-	-	-	-	-	-	1	11.11	-	-	-	-	-	-
Wottlin_2022	21	-	-	-	-	-	-	-	-	12	57.14	12	57.14	-	-	-	-	-	-
Zare_2014	14	-	-	7	50.00	3	21.43	7	50.00	-	-	8	57.14	-	-	-	-	0	0.00
Zare_2014	3	-	-	3	100.00	3	100.00	3	100.00	-	-	3	100.00	-	-	-	-	0	0.00
Zare_2014	4	-	-	4	100.00	3	75.00	4	100.00	-	-	4	100.00	-	-	-	-	0	0.00

Supplementary Material S8. Prevalence of antimicrobial resistance of *Salmonella* spp. by antimicrobial substance (continued)

Author	Pos (n)	Carbadox		Carbanecillin		Ciprofloxacin		Cefaclor		Cefepime		Cephalexin		Cephalotin		Cefazoline		Cefixime		Cefotaxime		Cefoxatin	
		n	p	n	p	n	p	n	p	n	p	n	p	n	p	n	p	n	p	n	p	n	p
Abrahan_2022	184	-	-	-	-	1	0.54	-	-	-	-	-	-	-	-	-	-	-	-	-	-	0	0.00
Alemu_2021	28	-	-	-	-	-	-	-	-	-	-	-	-	-	-	-	-	-	-	-	-	-	-
Alemu_2022	14	-	-	-	-	1	7.14	-	-	-	-	-	-	-	-	-	-	-	-	-	-	-	-
Al-Zubaid_2013	11	-	-	-	-	0	0.00	-	-	-	-	-	-	-	-	-	-	10	90.91	-	-	-	-
Amera_2017	19	-	-	-	-	0	0.00	-	-	-	-	-	-	-	-	-	-	-	-	-	-	-	-
Atlaw_2022	368	-	-	-	-	0	0.00	-	-	-	-	-	-	-	-	-	-	-	-	-	-	-	-
Bacon_2002	53	-	-	-	-	0	0.00	-	-	-	-	-	-	-	-	-	-	-	-	-	-	-	-
Barlow_2015	106	-	-	-	-	0	0.00	-	-	-	-	-	-	-	-	0	0.00	-	-	0	0.00	-	-
Biasi_2011	18	-	-	-	-	14	77.78	-	-	-	-	-	-	18	100.00	-	-	-	-	-	-	-	-
Bier_2018	6	-	-	-	-	0	0.00	-	-	-	-	-	-	-	-	-	-	-	-	0	0.00	-	-
Cetin_2020	10	-	-	-	-	1	10.00	-	-	1	10.00	-	-	-	-	-	-	-	-	0	0.00	-	-
Cetin_2018	8	-	-	-	-	-	-	-	-	-	-	-	-	-	-	-	-	-	-	1	12.50	-	-
Edrington_2009	0	-	-	-	-	-	-	-	-	-	-	-	-	3	-	-	-	-	-	-	-	-	-

Ekli_2019	31	-	-	-	-	0	0.00	-	-	-	-	-	-	-	-	-	-	-	-	-	-	-	-
Ferede_2015	44	-	-	-	-	0	0.00	-	-	-	-	-	-	-	-	-	-	-	-	-	-	-	-
Gabanan_2022	13	-	-	-	-	-	-	8	61.54	-	-	-	-	-	-	-	-	-	-	-	-	-	-
Geresu_2021	10	-	-	-	-	0	0.00	-	-	-	-	-	-	-	-	-	-	-	-	-	-	-	-
Gragg_2013	91	-	-	-	-	-	-	-	-	-	-	-	-	-	-	-	-	-	-	-	-	-	-
Guteman_2021	16	-	-	-	-	0	0.00	-	-	-	-	-	-	-	-	-	-	-	-	0	0.00	-	-
Jajere_2015	33	-	-	-	-	0	0.00	-	-	-	-	-	-	-	-	-	-	-	-	-	-	-	-
Jajere_2015	16	-	-	-	-	0	0.00	-	-	-	-	-	-	-	-	-	-	-	-	-	-	-	-
Jajeren_2015	4	-	-	-	-	0	0.00	-	-	-	-	-	-	-	-	-	-	-	-	-	-	-	-
Jitjak_2016	1	-	-	-	-	0	0.00	-	-	-	-	-	-	-	-	-	-	-	-	0	0.00	-	-
Kebede_2016	8	-	-	-	-	2	25.00	-	-	-	-	-	-	-	-	-	-	-	-	-	-	-	-
Ketema_2018	27	-	-	-	-	10	37.04	-	-	-	-	-	-	19	70.37	-	-	-	-	-	-	-	-
Kore_2017	10	-	-	-	-	0	0.00	-	-	-	-	-	-	-	-	-	-	-	-	-	-	-	-
Li_2019	80	-	-	-	-	6	7.50	-	-	-	-	-	-	-	-	-	-	-	-	-	-	-	-
Lynne_2009	18	-	-	-	-	0	0.00	-	-	-	-	-	-	-	-	-	-	-	-	-	-	-	-
Madoroba_2016	92	-	-	-	-	-	-	-	-	-	-	-	-	-	-	-	-	-	-	22	23.91	-	-
Mathole_2016	2	-	-	-	-	-	-	-	-	-	-	-	-	-	-	-	-	-	-	-	-	0	0.00
McEvoy_2003	29	-	-	-	-	-	-	-	-	-	-	-	-	-	-	-	-	-	-	-	-	-	-
Morar_2015	26	-	-	-	-	1	3.85	-	-	0	0.00	-	-	-	-	0	0.00	-	-	0	0.00	-	-
Mouichn_2018	84	-	-	-	-	12	14.29	-	-	0	0.00	-	-	0	0.00	10	11.90	-	-	0	0.00	-	-
Mustafa_2018	17	-	-	-	-	6	35.29	-	-	-	-	-	-	-	-	-	-	-	-	-	-	-	-
Obaidat_2020	287	-	-	-	-	77	26.83	-	-	-	-	-	-	0	40.07	-	-	-	-	0	0.00	-	-
Oueslati_2016	17	-	-	-	-	5	29.41	-	-	-	-	-	-	2	11.76	-	-	-	-	-	-	-	-
Shaibu_2021	11	-	-	-	-	-	-	-	-	0	0.00	-	-	-	-	-	-	-	-	-	-	-	-
Shilangale_2015	45	-	-	-	-	7	15.56	-	-	-	-	-	-	0	0.00	-	-	-	-	-	-	-	-
Sibhatn_2009	87	-	-	-	-	0	0.00	-	-	0	0.00	-	-	0	0.00	0	0.00	-	-	0	0.00	-	-
Stevens_2006	99	-	-	-	-	0	0.00	-	-	-	-	-	-	0	0.00	-	-	-	-	0	0.00	-	-
Takelen_2018	42	-	-	-	-	0	0.00	-	-	0	0.00	-	-	5	11.90	0	0.00	-	-	0	0.00	-	-
Varela_Guerrero_2013	27	-	-	-	-	-	-	-	-	-	-	-	-	-	-	-	-	-	-	-	-	-	-
Wabeto_2017	56	-	-	-	-	4	7.14	-	-	-	-	-	-	-	-	-	-	-	-	-	-	-	-
Wang_2020	41	-	-	-	-	-	-	-	-	-	-	-	-	-	-	-	-	-	-	-	-	-	-
Webb_2017	376	-	-	-	-	1	0.27	-	-	-	-	-	-	25	6.65	-	-	-	-	-	-	-	-
Wieczorek_2013	9	-	-	-	-	-	-	-	-	-	-	-	-	-	-	-	-	-	-	-	-	-	-
Wottlin_2022	21	-	-	-	-	-	-	-	-	-	-	-	-	-	-	-	-	-	-	-	-	-	-
Zare_2014	14	-	-	-	-	4	28.57	-	-	-	-	-	-	-	-	-	-	4	28.57	-	-	-	-

Zare_2014	3	-	-	-	-	3	100.0	-	-	-	-	-	-	-	-	-	-	3	100.0	-	-	-	-
Zare_2014	4	-	-	-	-	4	100.0	-	-	-	-	-	-	-	-	-	-	4	100.0	-	-	-	-

Supplementary Material S8. Prevalence of antimicrobial resistance of *Salmonella* spp. by antimicrobial substance (continued)

Author	PS (n)	Cefoxitin		Ceftiofur		Ceftriaxone		Ceftazidime		Cefuroxime		Clindamycin		Colistin		Cloranphenicol		Cloxacillin		Cotrimoxazole	
		n	p	n	p	n	p	n	p	n	p	n	p	n	p	n	p	n	p	n	p
Abrahan_2022	184	0	0.00	0	0.00	0	0.00	0	0.00	-	-	0	0.00	0	0.00	0	0.00	-	-	-	-
Alemu_2021	28	-	-	-	-	-	-	-	-	-	-	-	-	-	-	0	0.00	-	-	-	-
Alemu_2022	14	-	-	-	-	-	-	-	-	-	-	-	-	-	-	-	-	-	-	-	-
Al-Zubaid_2013	11	-	-	-	-	-	-	-	-	-	-	-	-	-	-	6	54.55	11	100.00	-	-
Amera_2017	19	13	68.42	-	-	-	-	-	-	-	-	-	-	0	0.00	0	0.00	-	-	0	0.00
Atlaw_2022	368	0	0.00	1	0.27	0	0.00	-	-	-	-	-	-	-	-	20	5.43	-	-	-	-
Bacon_2002	53	-	-	-	-	0	0.00	-	-	-	-	-	-	-	-	8	15.09	-	-	-	-
Barlow_2015	106	0	0.00	0	0.00	0	0.00	-	-	-	-	-	-	-	-	0	0.00	-	-	0	0.00
Bier_2018	6	-	-	-	-	-	-	-	-	-	-	-	-	-	-	0	0.00	-	-	-	-
Cetin_2020	10	0	0.00	-	-	-	-	-	-	-	-	-	-	-	-	0	0.00	-	-	-	-
Cetin_2018	8	1	12.50	-	-	-	-	-	-	-	-	-	-	-	-	-	-	-	-	-	-
Edrington_2009	0	-	-	-	-	-	-	-	-	-	-	-	-	-	-	-	-	-	-	-	-
Ekli_2019	31	-	-	-	-	0	0.00	-	-	-	-	-	-	-	-	0	0.00	-	-	-	-
Ferede_2015	44	-	-	-	-	10	22.73	-	-	-	-	-	-	-	-	20	45.45	-	-	-	-
Gabanan_2022	13	-	-	0	0.00	-	-	-	-	-	-	-	-	-	-	11	84.62	-	-	-	-
Geresu_2021	10	0	0.00	-	-	-	-	-	-	-	-	0	0.00	-	-	0	0.00	-	-	-	-
Gragg_2013	91	2	2.20	-	-	-	-	-	-	-	-	-	-	-	-	0	0.00	-	-	-	-
Guteman_2021	16	-	-	-	-	-	-	0	0.00	-	-	-	-	0	0.00	8	50.00	-	-	-	-
Jajere_2015	33	-	-	-	-	0	0.00	-	-	-	-	-	-	-	-	0	0.00	-	-	32	96.97
Jajere_2015	16	-	-	-	-	0	0.00	-	-	-	-	-	-	-	-	0	0.00	-	-	0	0.00
Jajere_2015	4	-	-	-	-	0	0.00	-	-	-	-	-	-	-	-	0	0.00	-	-	0	0.00
Jitjak_2016	1	-	-	-	-	-	-	-	-	-	-	-	-	-	-	0	0.00	-	-	0	0.00
Kebede_2016	8	-	-	-	-	2	25.00	-	-	-	-	-	-	-	-	2	25.00	-	-	-	-
Ketema_2018	27	11	40.74	-	-	0	0.00	-	-	-	-	-	-	-	-	0	0.00	-	-	0	0.00
Kore_2017	10	0	0.00	0	0.00	0	0.00	-	-	-	-	-	-	-	-	0	0.00	-	-	-	-
Lynne_2009	18	7	38.89	3	16.67	0	0.00	-	-	-	-	-	-	-	-	9	50.00	-	-	-	-
Madoroba_2016	92	-	-	-	-	-	-	-	-	-	-	0	0.00	-	-	-	-	-	-	-	-
Mathole_2016	2	-	-	-	-	-	-	-	-	-	-	-	-	-	-	-	-	-	-	-	-
McEvoy_2003	29	-	-	-	-	-	-	-	-	-	-	-	-	-	-	4	13.79	-	-	-	-

Morar_2015	26	0	0.00	-	-	-	-	0	0.00	-	-	-	-	-	-	-	-	-	-	-	-
Mouichn_2018	84	0	0.00	0	0.00	0	0.00	0	0.00	-	-	0	0.00	0	0.00	2	2.38	-	-	-	-
Mustafa_2018	17	-	-	-	-	-	-	-	-	1	5.88	-	-	-	-	0	0.00	-	-	-	-
Obaidat_2020	287	-	-	-	-	55	19.16	-	-	-	-	0	0.00	-	-	74	25.78	-	-	-	-
Oueslati_2016	17	5	29.41	-	-	-	-	0	0.00	-	-	-	-	-	-	1	5.88	-	-	-	-
Shaibu_2021	11	-	-	-	-	-	-	-	-	-	-	-	-	-	-	1	9.09	-	-	-	-
Sibhatn_2009	87	0	0.00	0	0.00	-	-	-	-	-	-	-	-	0	0.00	-	-	-	-	-	-
Stevens_2006	99	0	0.00	-	-	-	-	0	0.00	-	-	-	-	-	-	2	2.02	-	-	-	-
Takelen_2018	42	0	0.00	0	0.00	0	0.00	0	0.00	-	-	0	0.00	0	0.00	5	11.90	-	-	-	-
Varela_Guerrero_2013	27	-	-	-	-	-	-	-	-	-	-	-	-	-	-	-	-	-	-	-	-
Wabeto_2017	56	-	-	-	-	13	23.21	-	-	-	-	21	37.50	-	-	29	51.79	-	-	-	-
Wang_2020	41	-	-	16	39.02	-	-	-	-	-	-	-	-	-	-	-	-	-	-	-	-
Webb_2017	376	-	-	-	-	7	1.86	-	-	-	-	-	-	-	-	32	8.51	-	-	-	-
Wieczorek_2013	9	-	-	-	-	-	-	-	-	-	-	-	-	0	0.00	0	0.00	-	-	-	-
Wottlin_2022	21	10	47.62	-	-	10	47.62	-	-	-	-	-	-	-	-	12	57.14	-	-	-	-
Zare_2014	14	-	-	-	-	5	35.71	-	-	-	-	-	-	8	57.14	5	35.71	-	-	-	-
Zare_2014	3	-	-	-	-	3	100.00	-	-	-	-	-	-	3	100.00	3	100.00	-	-	-	-
Zare_2014	4	-	-	-	-	4	100.00	-	-	-	-	-	-	4	100.00	4	100.00	-	-	-	-

Supplementary Material S8. Prevalence of antimicrobial resistance of *Salmonella* spp. by antimicrobial substance (continued)

Author	PS (n)	Dalfopristin		Doxicilin		Ertapenem		Erytromicine		Enfloxacin		Florfenicol		Flumequine		Fosfomicyn		Furazolidone		Gentamicyn	
		n	p	n	p	n	p	n	p	n	p	n	p	n	p	n	p	n	p	n	p
Abrahan_2022	184	-	-	-	-	-	-	-	-	-	-	0	0.00	-	-	-	-	-	-	0	0.00
Alemu_2021	28	-	-	-	-	-	-	-	-	-	-	-	-	-	-	-	-	-	-	0	0.00
Alemu_2022	14	-	-	-	-	-	-	-	-	-	-	-	-	-	-	-	-	-	-	-	-
Al-Zubaid_2013	11	-	-	-	-	-	-	-	-	-	-	-	-	-	-	-	-	-	-	6	54.55
Amera_2017	19	-	-	-	-	-	-	-	-	-	-	-	-	-	-	-	-	-	-	0	0.00
Atlaw_2022	368	-	-	-	-	-	-	-	-	-	-	-	-	-	-	-	-	-	-	1	0.27
Bacon_2002	53	-	-	-	-	-	-	-	-	0	0.00	-	-	-	-	-	-	-	-	4	7.55
Barlow_2015	106	-	-	-	-	-	-	-	-	-	-	0	0.00	-	-	-	-	-	-	0	0.00
Bier_2018	6	-	-	-	-	-	-	-	-	-	-	-	-	-	-	-	-	-	-	0	0.00
Cetin_2020	10	-	-	-	-	0	0.00	-	-	-	-	-	-	-	-	-	-	-	-	0	0.00
Cetin_2018	8	-	-	-	-	-	-	-	-	-	-	-	-	-	-	-	-	-	-	1	12.50
Edrington_2009	0	-	-	-	-	-	-	3	-	-	-	0	-	-	-	-	-	-	-	-	-

Ekli_2019	31	-	-	-	-	-	-	-	-	-	-	-	-	-	-	-	-	-	-	4	12.90
Ferede_2015	44	-	-	-	-	-	-	-	-	-	-	-	-	-	-	-	-	-	-	8	18.18
Gabanan_2022	13	-	-	-	-	-	-	-	-	0	0.00	-	-	-	-	-	-	-	-	5	38.46
Geresu_2021	10	-	-	-	-	-	-	-	-	0	0.00	-	-	-	-	-	-	-	-	0	0.00
Gragg_2013	91	-	-	-	-	-	-	-	-	-	-	-	-	-	-	-	-	-	-	-	-
Guteman_2021	16	-	-	-	-	-	-	-	-	-	-	-	-	-	-	-	-	-	-	0	0.00
Iglesias_2017	16	-	-	-	-	-	-	0	0.00	-	-	-	-	-	-	-	-	-	-	1	6.25
Jajere_2015	33	-	-	-	-	-	-	-	-	-	-	-	-	-	-	-	-	-	-	0	0.00
Jajere_2015	16	-	-	-	-	-	-	-	-	-	-	-	-	-	-	-	-	-	-	0	0.00
Jajere_2015	4	-	-	-	-	-	-	-	-	-	-	-	-	-	-	-	-	-	-	0	0.00
Jitjak_2016	1	-	-	-	-	-	-	-	-	-	-	-	-	-	-	-	-	-	-	0	0.00
Kebede_2016	8	-	-	-	-	-	-	-	-	-	-	-	-	-	-	-	-	-	-	0	0.00
Ketema_2018	27	-	-	-	-	-	-	19	70.37	-	-	-	-	-	-	-	-	-	-	2	7.41
Kore_2017	10	-	-	-	-	-	-	-	-	-	-	-	-	-	-	-	-	-	-	0	0.00
Lynne_2009	18	-	-	-	-	-	-	-	-	-	-	-	-	-	-	-	-	-	-	7	38.89
Madoroba_2016	92	-	-	-	-	-	-	-	-	10	10.87	-	-	-	-	-	-	-	-	-	-
Mathole_2016	2	-	-	-	-	-	-	-	-	0	0.00	0	0.00	-	-	-	-	-	-	-	-
McEvoy_2003	29	-	-	-	-	-	-	-	-	-	-	-	-	-	-	-	-	-	-	-	-
Morar_2015	26	-	-	-	-	-	-	-	-	-	-	-	-	-	-	-	-	-	-	0	0.00
Mouichn_2018	84	-	-	0	0.00	0	0.00	0	0.00	0	0.00	0	0.00	-	-	0.00	0.00	0	0.00	9	10.71
Mustafa_2018	17	-	-	-	-	-	-	-	-	-	-	-	-	-	-	-	-	-	-	5	29.41
Obaidat_2020	287	0.00	0.00	-	-	-	-	115	40.07	-	-	-	-	-	-	-	-	-	-	41	14.29
Oueslati_2016	17	-	-	-	-	-	-	2	11.76	-	-	-	-	-	-	-	-	-	-	4	23.53
Shaibu_2021	11	-	-	3	27.27	-	-	-	-	0	0.00	-	-	-	-	-	-	-	-	0	0.00
Shilangale_2015	45	-	-	-	-	-	-	0	0.00	-	-	-	-	-	-	-	-	-	-	1	2.22
Sibhatn_2009	87	-	-	0	0.00	0	0.00	0	0.00	0	0.00	0	0.00	-	-	-	-	0	0.00	0	0.00
Stevens_2006	99	-	-	-	-	-	-	0	0.00	-	-	-	-	-	-	-	-	-	-	0	0.00
Takelen_2018	42	-	-	0	0.00	0	0.00	5	11.90	0	0.00	0	0.00	-	-	-	-	0	0.00	6	14.29
Varela_Guerrero_2013	27	-	-	-	-	-	-	-	-	-	-	3	11.11	-	-	-	-	-	-	-	-
Wabeto_2017	56	-	-	-	-	-	-	-	-	-	-	-	-	-	-	-	-	-	-	7	12.50
Wang_2020	41	-	-	-	-	-	-	-	-	11	26.83	13	31.71	-	-	-	-	-	-	11	26.83
Webb_2017	376	-	-	-	-	-	-	25	6.65	-	-	-	-	-	-	-	-	-	-	0	0.00
Wieczorek_2013	9	-	-	-	-	-	-	-	-	-	-	0	0.00	-	-	-	-	-	-	0	0.00
Wottlin_2022	21	-	-	-	-	-	-	-	-	-	-	-	-	-	-	-	-	-	-	-	-
Zare_2014	14	-	-	-	-	-	-	-	-	6	42.86	-	-	-	-	-	-	-	-	7	50.00

Zare_2014	3	-	-	-	-	-	-	-	-	3	100.00	-	-	-	-	-	-	-	-	3	100.0
Zare_2014	4	-	-	-	-	-	-	-	-	4	100.00	-	-	-	-	-	-	-	-	4	100.0

Supplementary Material S8. Prevalence of antimicrobial resistance of *Salmonella* spp. by antimicrobial substance (continued)

Author	PS (n)	Imipenem		Kanamicin		Levofloxacin		Lincomycin		Linezolid		Mecillinan		Meropenen		Mezlocillin		Nalidixic acid		Norfloxacin		Neomicin	
		n	p	n	p	n	p	n	p	n	p	n	p	n	p	n	p	n	p	n	p	n	p
Abrahan_2022	184	-	-	-	-	-	-	-	-	-	-	-	-	0	0.00	-	-	0	0.00	-	-	-	-
Alemu_2021	28	-	-	-	-	-	-	-	-	-	-	-	-	-	-	-	-	-	-	0	0.00	-	-
Alemu_2022	14	-	-	-	-	-	-	-	-	-	-	-	-	-	-	-	-	1	7.14	-	-	1	7.14
Al-Zubaid_2013	11	-	-	5	45.45	-	-	-	-	-	-	-	-	-	-	-	-	-	-	-	-	3	27.27
Amera_2017	19	-	-	-	-	-	-	-	-	-	-	-	-	-	-	-	-	3	15.79	1	5.26	-	-
Atlaw_2022	368	-	-	-	-	-	-	-	-	-	-	-	-	-	-	-	-	4	1.09	-	-	-	-
Bacon_2002	53	-	-	-	-	0	0.00	-	-	-	-	-	-	-	-	-	-	-	-	-	-	-	-
Barlow_2015	106	0	0.00	0	0.00	-	-	-	-	-	-	-	-	0	0.00	-	-	0	0.00	-	-	-	-
Bier_2018	6	-	-	-	-	-	-	-	-	-	-	-	-	-	-	-	-	-	-	-	-	-	-
Cetin_2020	10	-	-	-	-	-	-	-	-	-	-	0	0.00	-	-	-	-	-	-	1	10.00	-	-
Cetin_2018	8	-	-	-	-	-	-	-	-	-	-	-	-	-	-	-	-	-	-	1	12.50	-	-
Edrington_2009	0	-	-	1	-	-	-	-	-	-	-	-	-	-	-	-	-	-	-	-	-	0	-
Ekli_2019	31	-	-	-	-	-	-	-	-	-	-	-	-	-	-	-	-	-	-	-	-	-	-
Ferede_2015	44	-	-	35	79.55	-	-	-	-	-	-	-	-	-	-	-	-	25	56.82	-	-	-	-
Gabanan_2022	13	13	100.00	-	-	-	-	13	100.00	-	-	-	-	-	-	-	-	11	84.62	-	-	9	69.23
Geresu_2021	10	-	-	0	0.00	-	-	-	-	-	-	-	-	-	-	-	-	0	0.00	0	0.00	1	10.00
Gragg_2013	91	-	-	-	-	-	-	-	-	-	-	-	-	-	-	-	-	2	2.20	-	-	-	-
Guteman_2021	16	-	-	-	-	-	-	-	-	-	-	-	-	0	0.00	-	-	0	0.00	-	-	-	-
Iglesias_2017	16	0	0.00	1	6.25	-	-	-	-	-	-	-	-	-	-	-	-	0	0.00	-	-	-	-
Jajere_2015	33	-	-	33	100.00	-	-	-	-	-	-	-	-	-	-	-	-	-	-	0	0.00	-	-
Jajere_2015	16	-	-	15	93.75	-	-	-	-	-	-	-	-	-	-	-	-	-	-	-	-	-	-
Jajere_2015	4	-	-	4	100.00	-	-	-	-	-	-	-	-	-	-	-	-	-	-	-	-	-	-
Jitjak_2016	1	-	-	0	0.00	-	-	-	-	-	-	-	-	-	-	-	-	0	0.00	-	-	-	-
Kebede_2016	8	-	-	2	25.00	-	-	-	-	-	-	-	-	-	-	-	-	1	12.50	-	-	-	-
Ketema_2018	27	-	-	16	59.26	-	-	-	-	-	-	-	-	-	-	-	-	2	7.41	10	37.04	-	-
Kore_2017	10	-	-	-	-	-	-	-	-	-	-	-	-	-	-	-	-	0	0.00	-	-	-	-
Lynne_2009	18	-	-	11	61.11	-	-	-	-	-	-	-	-	-	-	-	-	0	0.00	-	-	-	-
Madoroba_2016	92	-	-	26	28.26	-	-	-	-	-	-	-	-	-	-	-	-	-	-	-	-	-	-
Mathole_2016	2	-	-	0	0.00	-	-	-	-	-	-	-	-	-	-	-	-	-	-	-	-	-	-

McEvoy_2003	29	-	-	-	-	-	-	-	-	-	-	-	-	-	-	-	-	1	3.45	-	-	-	-
Morar_2015	26	0	0.00	-	-	-	-	-	-	-	-	-	-	-	-	-	-	-	-	6	23.08	-	-
Mouichn_2018	84	0	0.00	0	0.00	-	-	-	-	0	0.00	4	4.76	-	-	0	0.00	15	17.86	11	13.10	0	0.00
Mustafa_2018	17	-	-	3	17.65	-	-	-	-	-	-	-	-	-	-	-	-	-	-	-	-	-	-
Obaidat_2020	287	-	-	83	28.92	-	-	-	-	0	0.00	-	-	-	-	-	-	245	85.37	-	-	-	-
Oueslati_2016	17	-	-	5	29.41	-	-	-	-	-	-	-	-	-	-	-	-	5	29.41	-	-	-	-
Shaibu_2021	11	-	-	-	-	-	-	-	-	-	-	-	-	-	-	-	-	-	-	-	-	-	-
Shilangale_2015	45	-	-	-	-	-	-	-	-	-	-	-	-	-	-	-	-	4	8.89	-	-	-	-
Sibhatn_2009	87	0	0.00	0	0.00	-	-	-	-	0	0.00	0	0.00	-	-	0	0.00	0	0.00	0	0.00	0	0.00
Stevens_2006	99	-	-	-	-	-	-	-	-	-	-	-	-	-	-	-	-	1	1.01	1	1.01	-	-
Takelen_2018	42	0	0.00	7	16.67	-	-	-	-	0	0.00	0	0.00	-	.	0	0.00	0	0.00	0	0.00	0	0.00
Varela_Guerrero_2013	27	-	-	-	-	-	-	-	-	-	-	-	-	-	-	-	-	12	44.44	-	-	-	-
Wabeto_2017	56	-	-	29	51.79	-	-	-	-	-	-	-	-	-	-	-	-	22	39.29	-	-	-	-
Wang_2020	41	0	0.00	-	-	-	-	-	-	-	-	-	-	-	-	-	-	-	-	-	-	-	-
Webb_2017	376	-	-	12	3.19	-	-	-	-	-	-	-	-	-	-	-	-	9	2.39	-	-	-	-
Wieczorek_2013	9	-	-	-	-	-	-	-	-	-	-	-	-	-	-	-	-	0	0.00	-	-	-	-
Wottlin_2022	21	-	-	-	-	-	-	-	-	-	-	-	-	-	-	-	-	-	-	-	-	-	-
Zare_2014	14	-	-	3	21.43	-	-	-	-	-	-	-	-	-	-	-	-	-	-	-	-	-	-
Zare_2014	3	-	-	3	100.00	-	-	-	-	-	-	-	-	-	-	-	-	-	-	-	-	-	-
Zare_2014	4	-	-	3	75.00	-	-	-	-	-	-	-	-	-	-	-	-	-	-	-	-	-	-

Supplementary Material S8. Prevalence of antimicrobial resistance of *Salmonella* spp. by antimicrobial substance (continued)

Author	PS (n)	Netilmicin		Nitrofurantoin		Orfloxacin		Pefloxacin		Penicilin		Piperacillin		Polimixin		Quinupristin		Rifampicin		Teicoplanin		Tri-sulfa	
		n	p	n	p	n	p	n	p	n	p	n	p	n	p	n	p	n	p	n	p	n	p
Abrahan_2022	184	-	-	-	-	-	-	-	-	-	-	0	0.00	-	-	-	-	-	-	0	0.00	-	-
Alemu_2021	28	-	-	-	-	-	-	-	-	-	-	-	-	-	-	0	0.00	-	-	0	0.00	-	-
Alemu_2022	14	-	-	-	-	-	-	-	-	-	-	1	7.14	-	-	-	-	-	-	-	-	-	-
Al-Zubaid_2013	11	-	-	3	27.27	-	-	-	-	-	-	-	-	-	-	-	-	-	-	-	-	-	-
Amera_2017	19	-	-	-	-	-	-	-	-	-	-	-	-	-	-	-	-	-	-	-	-	-	-
Atlaw_2022	368	-	-	-	-	-	-	-	-	-	-	2	0.54	-	-	-	-	-	-	0	0.00	-	-
Bacon_2002	53	-	-	-	-	-	-	-	-	-	-	2	3.77	-	-	-	-	-	-	-	-	-	-
Barlow_2015	106	-	-	-	-	-	-	-	-	-	-	8	7.55	-	-	-	-	-	-	0	0.00	-	-
Bier_2018	6	-	-	-	-	-	-	-	-	-	-	-	-	-	-	-	-	-	-	-	-	-	-
Cetin_2020	10	-	-	-	-	-	-	0	0.00	-	-	1	10.00	0	0.00	-	-	-	-	-	-	-	-

Cetin_2018	8	-	-	-	-	-	-	1	12.50	0	0.00	-	-	-	-	-	-	-	-	0	0.00	-	-
Edrington_2009	0	-	-	-	-	-	-	-	-	-	-	0	0.00	-	-	-	-	-	-	0	-	-	-
Ekli_2019	31	-	-	-	-	-	-	-	-	-	-	0	0.00	-	-	-	-	-	-	-	-	30	96.77
Ferede_2015	44	44	100.00	-	-	-	-	-	-	-	-	-	-	-	-	-	-	-	-	-	-	-	-
Gabanan_2022	13	-	-	-	-	13	100.00	-	-	13	100.00	6	46.15	-	-	-	-	-	-	-	-	-	-
Geresu_2021	10	-	-	-	-	-	-	-	-	-	-	0	0.00	-	-	-	-	-	-	0	0.00	-	-
Gragg_2013	91	-	-	-	-	-	-	-	-	-	-	4	4.40	-	-	-	-	-	-	-	-	-	-
Guteman_2021	16	-	-	-	-	-	-	-	-	-	-	-	-	-	-	-	-	-	-	-	-	-	-
Iglesias_2017	16	-	-	-	-	-	-	-	-	-	-	0	0.00	-	-	-	-	-	-	0	0.00	-	-
Jajere_2015	33	-	-	30	90.91	-	-	0	0.00	0	0.00	0	0.00	0	0.00	-	-	-	-	0	0.00	-	-
Jajere_2015	16	-	-	10	62.50	-	-	-	-	-	-	-	-	-	-	-	-	-	-	0	0.00	-	-
Jajere_2015	4	-	-	3	75.00	-	-	-	-	-	-	0	0.00	-	-	-	-	-	-	0	0.00	-	-
Jitjak_2016	1	-	-	-	-	-	-	-	-	1	100.00	-	-	1	100.00	-	-	-	-	-	-	-	-
Kebede_2016	8	-	-	5	62.50	-	-	-	-	-	-	3	37.50	-	-	-	-	-	-	-	-	-	-
Ketema_2018	27	-	-	10	37.04	-	-	-	-	-	-	1	3.70	-	-	-	-	-	-	0	0.00	-	-
Kore_2017	10	-	-	-	-	-	-	-	-	-	-	0	0.00	-	-	-	-	-	-	-	-	-	-
Lynne_2009	18	-	-	-	-	-	-	-	-	-	-	9	50.00	-	-	-	-	-	-	-	-	-	-
Madoroba_2016	92	-	-	-	-	-	-	-	-	-	-	-	-	-	-	-	-	-	-	0	0.00	-	-
Mathole_2016	2	-	-	-	-	-	-	-	-	-	-	-	-	-	-	2	100.00	-	-	-	-	-	-
McEvoy_2003	29	-	-	-	-	-	-	-	-	-	-	-	-	-	-	-	-	-	-	-	-	-	-
Morar_2015	26	-	-	6	23.08	-	-	-	-	-	-	9	34.62	2	7.69	-	-	-	-	-	-	-	-
Mouichn_2018	84	0	0.00	0	0.00	-	-	0	0.00	0	0.00	2	2.38	15	17.86	-	-	-	-	0	0.00	-	-
Mustafa_2018	17	-	-	0	0.00	-	-	-	-	-	-	-	-	-	-	-	-	-	-	-	-	-	-
Obaidat_2020	287	-	-	-	-	-	-	-	-	0	0.00	104	36.24	-	-	-	-	0	0.00	0	0.00	-	-
Oueslati_2016	17	-	-	-	-	-	-	-	-	-	-	-	-	-	-	-	-	-	-	-	-	-	-
Shaibu_2021	11	-	-	-	-	-	-	-	-	-	-	3	27.27	-	-	-	-	-	-	-	-	-	-
Shilangale_2015	45	-	-	-	-	-	-	-	-	-	-	7	15.56	-	-	-	-	-	-	-	-	-	-
Sibhatn_2009	87	-	-	0	0.00	-	-	0	0.00	0	0.00	0	0.00	0	0.00	-	-	-	-	0	0.00	-	-
Stevens_2006	99	-	-	36	36.36	-	-	1	1.01	-	-	0	0.00	-	-	-	-	-	-	-	-	-	-
Takelen_2018	42	-	-	0	0.00	-	-	0	0.00	0	0.00	5	11.90	0	0.00	-	-	-	-	-	-	-	-
Varela_Guerrero_2013	27	-	-	-	-	-	-	-	-	-	-	-	-	-	-	-	-	-	-	-	-	-	-
Wabeto_2017	56	-	-	41	73.21	-	-	-	-	-	-	-	-	-	-	-	-	-	-	-	-	-	-
Wang_2020	41	-	-	-	-	-	-	-	-	-	-	2	4.88	-	-	0	0.00	-	-	-	-	-	-
Webb_2017	376	-	-	-	-	-	-	-	-	-	-	1	0.27	-	-	-	-	-	-	-	-	-	-
Wieczorek_2013	9	-	-	-	-	-	-	-	-	-	-	-	-	-	-	-	-	-	-	0	0.00	-	-

Wottlin_2022	21	-	-	-	-	-	-	-	-	-	-	1	4.76	-	-	-	-	-	-	-	-	-	-
Zare_2014	14	-	-	-	-	-	-	-	-	-	-	-	-	-	-	-	-	-	-	-	-	-	-
Zare_2014	3	-	-	-	-	-	-	-	-	-	-	-	-	-	-	-	-	-	-	-	-	-	-
Zare_2014	4	-	-	-	-	-	-	-	-	-	-	-	-	-	-	-	-	-	-	-	-	-	-

Supplementary Material S8. Prevalence of antimicrobial resistance of *Salmonella* spp. by antimicrobial substance (continued)

Author	PS (n)	Trimethoprim		Tigecycline		Ticarcillin		Tobramycin		Tetracycline		Tilmicosyn		Spectinomicyn		Streptomicyn		Sulfisoxazole		Sulfadiazine	
		N	p	n	p	n	p	n	p	n	p	n	p	n	p	n	p	n	p	n	p
Abrahan_2022	184	-	-	-	-	-	-	-	-	0	0.00	-	-	-	-	7	3.80	-	-	-	-
Alemu_2021	28	0	0.00	-	-	-	-	-	-	0	0.00	-	-	-	-	7	25.00	-	-	-	-
Alemu_2022	14	-	-	-	-	-	-	-	-	2	14.29	-	-	-	-	2	14.29	-	-	-	-
Al-Zubaid_2013	11	-	-	-	-	-	-	-	-	5	45.45	-	-	-	-	0	0.00	8	72.73	-	-
Amera_2017	19	-	-	-	-	-	-	-	-	10	52.63	-	-	-	-	-	-	-	-	-	-
Atlaw_2022	368	-	-	-	-	-	-	-	-	77	20.92	-	-	-	-	42	11.41	56	15.22	-	-
Bacon_2002	53	-	-	-	-	-	-	-	-	17	32.08	-	-	-	-	17	32.08	-	-	-	-
Barlow_2015	106	-	-	-	-	-	-	-	-	7	6.60	-	-	-	-	9	8.49	-	-	-	-
Bier_2018	6	-	-	-	-	-	-	-	-	0	0.00	-	-	-	-	-	-	-	-	-	-
Cetin_2020	10	-	-	1	10.00	-	-	1	10.00	-	-	-	-	-	-	-	-	-	-	-	-
Cetin_2018	8	-	-	-	-	-	-	2	25.00	-	-	-	-	-	-	-	-	-	-	-	-
Edrington_2009	0	-	-	-	-	-	-	-	-	-	-	-	-	-	-	-	-	-	-	-	-
Ekli_2019	31	-	-	-	-	-	-	-	-	0	0.00	-	-	-	-	-	-	-	-	-	-
Ferede_2015	44	-	-	-	-	-	-	-	-	44	100.00	-	-	-	-	36	81.82	-	-	-	-
Gabanan_2022	13	-	-	-	-	-	-	-	-	13	100.00	-	-	-	-	0	0.00	13	100.00	-	-
Geresu_2021	10	-	-	-	-	-	-	-	-	4	40.00	-	-	-	-	5	50.00	-	-	-	-
Gragg_2013	91	-	-	-	-	-	-	-	-	52	57.14	-	-	-	-	15	16.48	12	13.19	-	-
Guteman_2021	16	0	0.00	0	0.00	-	-	-	-	7	43.75	-	-	-	-	-	-	8	50.00	-	-
Iglesias_2017	16	-	-	-	-	-	-	-	-	0	0.00	-	-	-	-	-	-	-	-	-	-
Jajere_2015	33	0	0.00	-	-	-	-	-	-	28	84.85	-	-	-	-	-	-	-	-	-	-
Jajere_2015	16	-	-	-	-	-	-	-	-	12	75.00	-	-	-	-	16	100.00	0	0.00	-	-
Jajere_2015	4	-	-	-	-	-	-	-	-	2	50.00	-	-	-	-	4	100.00	-	-	-	-
Jitjak_2016	1	-	-	-	-	-	-	-	-	0	0.00	-	-	-	-	0	0.00	0	0.00	-	-
Kebede_2016	8	-	-	-	-	-	-	-	-	2	25.00	-	-	-	-	8	100.00	-	-	-	-
Ketema_2018	27	-	-	-	-	-	-	-	-	11	40.74	-	-	-	-	25	92.59	12	44.44	-	-
Kore_2017	10	-	-	-	-	-	-	-	-	0	0.00	-	-	-	-	0	0.00	0	0.00	-	-
Lynne_2009	18	-	-	-	-	-	-	-	-	16	88.89	-	-	-	-	18	100.00	9	50.00	-	-

Madoroba_2016	92	-	-	-	-	-	-	-	-	49	53.26	-	-	-	-	-	-	-	-	-	-
Mathole_2016	2	0	0.00	-	-	-	-	-	-	0	0.00	-	-	-	-	-	-	-	-	-	-
McEvoy_2003	29	-	-	-	-	-	-	-	-	4	13.79	-	-	-	-	10	34.48	-	-	-	-
Morar_2015	26	-	-	-	-	-	-	-	-	16	61.54	-	-	-	-	-	-	-	-	-	-
Mouichn_2018	84	0	0.00	0	0.00	15	17.86	0	0.00	15	17.86	0	0.00	0	0.00	58	69.05	0	0.00	-	-
Mustafa_2018	17	-	-	-	-	-	-	-	-	-	-	-	-	-	-	7	41.18	-	-	-	-
Obaidat_2020	287	-	-	-	-	-	-	-	-	166	57.84	-	-	-	-	149	51.92	-	-	-	-
Oueslati_2016	17	-	-	-	-	-	-	-	-	12	70.59	-	-	-	-	10	58.82	-	-	-	-
Shaibu_2021	11	-	-	-	-	-	-	-	-	1	9.09	-	-	-	-	-	-	-	-	-	-
Shilangale_2015	45	-	-	-	-	-	-	-	-	7	15.56	-	-	-	-	-	-	17	37.78	-	-
Sibhatn_2009	87	0	0.00	0	0.00	0	0.00	0	0.00	13	14.94	0	0.00	0	0.00	1	1.15	0	0.00	-	-
Stevens_2006	99	0	0.00	-	-	0	0.00	-	-	0	0.00	-	-	-	-	14	14.14	-	-	-	-
Takelen_2018	42	0	0.00	0	0.00	0	0.00	0	0.00	14	33.33	0	0.00	0	0.00	15	35.71	0	0.00	-	-
Varela_Guerrero_2013	27	-	-	-	-	-	-	-	-	9	33.33	-	-	-	-	20	74.07	27	100.00	-	-
Wabeto_2017	56	-	-	-	-	-	-	-	-	6	10.71	-	-	-	-	37	66.07	-	-	-	-
Wang_2020	41	-	-	-	-	-	-	-	-	-	-	-	-	-	-	-	-	-	-	-	-
Webb_2017	376	-	-	-	-	-	-	-	-	59	15.69	-	-	-	-	36	9.57	38	10.11	-	-
Wieczorek_2013	9	-	-	-	-	-	-	-	-	1	11.11	-	-	-	-	1	11.11	2	22.22	-	-
Wottlin_2022	21	-	-	-	-	-	-	-	-	21	100.00	-	-	-	-	21	100.00	-	-	-	-
Zare_2014	14	-	-	-	-	-	-	-	-	9	64.29	-	-	-	-	8	57.14	-	-	-	-
Zare_2014	3	-	-	-	-	-	-	-	-	3	100.00	-	-	-	-	3	100.00	-	-	-	-
Zare_2014	4	-	-	-	-	-	-	-	-	4	100.00	-	-	-	-	4	100.00	-	-	-	-

Supplementary Material S8. Prevalence of antimicrobial resistance of *Salmonella* spp. by antimicrobial substance (continued)

Author	PS (n)	Sulphafurazole		Sulphametoxazol		Sulphonamide		Vancomicyn	
		n	p	n	p	n	p	n	p
Abrahan_2022	184	-	-	-	-	-	-	-	-
Alemu_2021	28	-	-	-	-	-	-	-	-
Alemu_2022	14	-	-	-	-	-	-	-	-
Al-Zubaid_2013	11	0	0.00	-	-	-	-	-	-
Amera_2017	19	-	-	-	-	-	-	-	-
Atlaw_2022	368	-	-	-	-	-	-	-	-
Bacon_2002	53	-	-	-	-	-	-	-	-

Barlow_2015	106	-	-	-	-	-	-	-	-
Bier_2018	6	-	-	-	-	-	-	-	-
Cetin_2020	10	-	-	-	-	-	-	-	-
Cetin_2018	8	-	-	-	-	-	-	-	-
Edrington_2009	0	-	-	-	-	-	-	-	-
Ekli_2019	31	-	-	-	-	-	-	-	-
Ferede_2015	44	-	-	-	-	19	43.18	-	-
Gabanan_2022	13	-	-	-	-	-	-	-	-
Geresu_2021	10	-	-	-	-	-	-	-	-
Gragg_2013	91	-	-	-	-	-	-	-	-
Guteman_2021	16	-	-	-	-	-	-	-	-
Iglesias_2017	16	-	-	-	-	-	-	0	0.00
Jajere_2015	33	-	-	-	-	-	-	0	0.00
Jajere_2015	16	-	-	-	-	-	-	-	-
Jajere_2015	4	-	-	-	-	-	-	0	0.00
Jitjak_2016	1	-	-	-	-	-	-	-	-
Kebede_2016	8	-	-	-	-	-	-	-	-
Ketema_2018	27	-	-	-	-	-	-	-	-
Kore_2017	10	-	-	-	-	-	-	-	-
Lynne_2009	18		0.00	-	-	-	-	-	-
Madoroba_2016	92	-	-	-	-	-	-	-	-
Mathole_2016	2	-	-	-	-	-	-	-	-
McEvoy_2003	29	4	13.79	-	-	-	-	-	-
Morar_2015	26	-	-	-	-	-	-	-	-
Mouichn_2018	84	-	-	-	-	-	-	-	-
Mustafa_2018	17	-	-	-	-	-	-	-	-
Obaidat_2020	287	-	-	-	-	-	-	0	0.00
Oueslati_2016	17	-	-	-	-	-	-	-	-
Shaibu_2021	11	-	-	-	-	--	-	-	-
Shilangale_2015	45	-	-	-	-	-	-	-	-
Sibhatn_2009	87	-	-	-	-	-	-	-	-
Stevens_2006	99	-	-	21	21.21	-	-	-	-
Takelen_2018	42	-	-	-	-	-	-	-	-
Varela_Guerrero_2013	27	-	-	-	-	-	-	-	-
Wabeto_2017	56	-	-	-	-	-	-	-	-

Wang_2020	41	-	-	-	-	-	-	-	-
Webb_2017	376	-	-	-	-	-	-	-	-
Wieczorek_2013	9	-	-	-	-	-	-	-	-
Wottlin_2022	21	-	-	-	-	-	-	-	-
Zare_2014	14	-	-	-	-	-	-	-	-
Zare_2014	3	-	-	-	-	-	-	-	-
Zare_2014	4	-	-	-	-	-	-	-	-
